# Differences in cortisol levels between preterm and term infants: a systematic review and meta-analysis combined with Mendelian randomization study

**DOI:** 10.3389/fped.2026.1790465

**Published:** 2026-06-02

**Authors:** Yun Li, Xiaohui Liang, Limin Cao, Yujing Yang, Shujing Wei, Yong Ji

**Affiliations:** 1 Department of Neonatal Intensive Care Unit (NICU), Shanxi Children's Hospital(Maternal and Child Health Hospital of Shanxi Province, Maternity Hospital of Shanxi Province), Taiyuan, China; 2 Department of Pediatrics, Shanxi Medical University, Taiyuan, China

**Keywords:** cortisol levels, hypothalamic-pituitary-adrenal axis, medelian randomization, meta-analysis, preterm infant

## Abstract

**Background:**

Preterm infants have an immaturity hypothalamic-pituitary-adrenal (HPA) axis, whether cortisol levels differ from those in term infants remains inconsistent. This study employed a meta-analysis to compare cortisol levels between preterm and term infants (PROSPERO: CRD42024606328, https://www.crd.york.ac.uk/PROSPERO/). We then applied Mendelian randomization (MR) to assess a potential causal relationship, with preterm birth as the exposure and cortisone (a key cortisol precursor) as the outcome.

**Methods:**

We systematically searched 10 databases from inception to August 2024 for studies reporting cortisol in preterm and term infants. Subgroup analyses and meta-regression were conducted by the type of specimens, measurement methods, measurement ages, gestational ages, measurement times, and the usage of steroid hormones. Random effects models were used for analysis, and data were reported using 95% confidence intervals. Publication bias was assessed using Eggers test, and a leave-one-out sensitivity analysis was performed. MR analysis was conducted to explore the causal relationship between preterm birth and cortisone.

**Results:**

A total of 74 studies were included, consisting of 18 umbilical cord blood (UCB) studies, 28 peripheral blood studies, and 31 saliva studies. Preterm infants had lower cortisol levels in UCB (SMD: −0.45; 95% CI: −0.77 to −0.12, *P* < 0.05) and the peripheral blood on the first day after birth (SMD: −0.46; −0.91 to −0.02, *P* < 0.05). Peripheral blood cortisol levels became higher in preterms after two weeks. Salivary cortisol did not differ. MR indicated a negative causal association between preterm birth and cortisone (*β* = −0.011, *P* = 0.035).

**Conclusion:**

Cortisol levels in preterm infants exhibit a trend of being initially lower, then higher, but finally comparable to those of term infants in the long term. MR supports a causal link between preterm birth and cortisone. These findings may guide the clinical management of preterm infants and individualized glucocorticoid strategies. Future high-quality clinical studies are required to further validate and optimize glucocorticoid protocols.

**Systematic Review Registration:**

https://www.crd.york.ac.uk/PROSPERO/. PROSPERO: CRD42024606328

## 1 Introduction

Preterm birth is the largest direct cause of neonatal death. Studies have reported that 14.9 million babies were born preterm worldwide in 2010 (1). In recent years, with the continuous improvement of the survival rate of premature infants (PI), clinical attention has shifted toward long-term outcomes and physiological stability, including circulatory management. Circulatory support is crucial for reducing the morbidity and mortality of PI, especially those who are extremely preterm. However, current circulatory management for PI still faces multiple challenges. Someone suggested using hydrocortisone to minimize complications associated with poor peripheral perfusion when comprehensive circulation management fails to achieve adequate perfusion (2). In addition to providing circulatory support, corticosteroids are commonly administered to prevent and treat pulmonary diseases by promoting lung maturation and enhancing respiratory function (3–6). They even advocated the necessity of supplementing the physiological requirements of cortisol. This is because the hypothalamic-pituitary-adrenal (HPA) axis in PI is immature, leading to lower cortisol levels. Most preterm infants need to receive treatment in the neonatal intensive care unit (NICU), exposing to multiple stressors such as procedures pain, respiratory support, and maternal separation. Such stressors can significantly influence HPA axis function and stimulate cortisol secretion, making the change of cortisol levels in preterm infants more complex. Despite the clinical interest, whether preterm infants have lower, higher, or similar cortisol levels compared to term infants remains debated.

Some studies have reported that cortisol was similar in the preterm and term groups despite the fact that there was a global defect in adrenal steroid synthesis pathways in PI (7). Conversely, other studies have found that PI often have higher cortisol levels due to the increased compensatory cortisol production triggered by greater post-birth stress, which helps maintain internal environmental stability (8). There is not only controversy in the early postnatal period but also different opinions in the long term. Furthermore, the trajectory of cortisol levels over the postnatal period and into childhood is poorly characterized. As a crucial biomarker of the stress response, the regulatory adaptations of cortisol interact in complex ways with stress and health throughout the life course (9). Some scholars argued that early exposure to the extrauterine environment could lead to a blunted cortisol response later in life (10–12). However, there are also reports indicating that the basal cortisol levels in PI are significantly higher than those of term infants at the corrected age of 8 and 18 months (13, 14), suggesting that early environmental stress can permanently reorganize hormonal, physiological and behavioral systems to influence the long-term changes of cortisol levels. In fact, determining the cortisol level is crucial to deciding whether steroid hormone support is necessary for preterms. Resolving these inconsistencies is crucial for guiding decisions on corticosteroid supplementation in preterm neonates with circulatory instability.

Given the current controversy regarding cortisol levels in preterm infants and recognizing that individual studies may not be able to provide sufficient data alone, we therefore conducted a systematic review and meta-analysis to compare cortisol levels between preterm and term infants across different biological samples [umbilical cord blood (UCB), peripheral blood, saliva] and postnatal ages. We also performed subgroup analyses based on measurement methods, gestational ages (GA), measurement times, and the usage of steroid hormones to explore potential sources of heterogeneity. Univariate and multivariable meta-regressions were conducted to identify the potential source of heterogeneity. This approach allowed us to clearly describe the variation patterns of cortisol in preterm infants.

Mendelian randomization (MR) is a well-established tool for inferring causal relationships. Cortisol and cortisone are both important glucocorticoids regulated by the HPA axis, with a close metabolic conversion relationship. 11-beta-hydroxysteroid dehydrogenases (11β-HSDs) catalyse the conversion of cortisol and cortisone (15). This cortisol-cortisone shuttle is crucial for glucocorticoid activity. Although cortisol is the primary bioactive glucocorticoid, large-scale genome-wide association study (GWAS) data for cortisol are currently limited. Previous research has shown that cortisol and its biosynthetic precursors in preterm infants follow synchronized trends after birth (16–18). As a metabolic precursor of cortisol, cortisone exhibits a significant linear correlation with serum cortisol (19, 20). Therefore, we used cortisone as a proxy to investigate whether preterm birth causally influences glucocorticoid metabolism via MR analysis.

## 2 Methods

### 2.1 Meta analysis

This meta-analysis was consistent with the Preferred Reporting Items for Systematic Reviews and Meta-Analyses (PRISMA) Statement (21) and it had been registered at the International Prospective Register of Systematic Reviews (PROSPERO; registration number: CRD42024606328). The completed PRISMA 2020 checklist is provided as Supplementary Table S10.

#### 2.1.1 Search strategy

To search for case-control studies on cortisol levels in preterm infants, we systematically searched several databases. For English databases, we searched Pubmed, EMBASE, Web of Science, Cochrane Library, Scopus, and ScienceDirect. For Chinese databases, we searched the China National Knowledge Infrastructure (CNKI), Chinese Biomedical Literature Database (CBM), Wan Fang database, and Weipu (VIP) database. The retrieval period for each of these databases spanned from their respective inception dates to August 1, 2024. The retrieval strategy adopted the combination of subject terms and free words according to the characteristics of different databases. The detailed search strings for each database are provided in Supplementary Table S1, incorporating search terms like “hydrocortisone”, “cortisol”, “Premature Birth”, and “Infant, Premature”. A manual search of reference lists was also performed to avoid studies that might have been missed. We considered all potentially relevant studies for this meta-analysis, neglecting the purpose and results of the original study.

#### 2.1.2 Inclusion and exclusion criteria

Inclusion criteria of articles in the analysis were as follows: (1) The study types included case-control studies, cohort studies, or retrospective or cross-sectional studies in which the case group and control group were divided and cortisol measurements were conducted. (2) The study subjects were preterm infants (GA < 37 weeks), and the control group consisted of healthy term infants (37 weeks ≤ GA < 42 weeks). (3) Original study, sample type included cord blood, peripheral blood or saliva. (4) Complete data can be obtained or the mean and standard deviation of cortisol levels in preterm and term infants can be calculated. (5) Full text available. No upper limit was set for postnatal age at cortisol measurement; studies with follow-up extending from birth through childhood, adolescence, and adulthood were eligible for inclusion.

Exclusion criteria were as follows: (1) Reviews, guidelines, meta-analyses, editorials, case reports, commentaries, letters to the editor, and other studies that provided no raw data. (2) Studies in animal models, cell culture, or *in vitro*. (3) Studies with duplicate publication of data. (4) Studies with incomplete data.

#### 2.1.3 Data extraction

Two independent investigators reviewed the titles and abstracts, and the studies that satisfied the inclusion criteria were retrieved for a full-text assessment. Data assessment and extraction were conducted independently by two investigators, and disagreements would be discussed with the third investigator. When two or more articles used the same original population data, only the article with the larger sample size was included in this meta-analysis. After identifying all articles that met the inclusion criteria, data were extracted using a standardized form, containing: the article title, the name of the first author, publication date, country, and basic characteristics of the sample, including sample size in the preterm and term groups, cortisol levels in the preterm and term groups, type of specimen, GA, prenatal steroid hormones use, postnatal steroid hormones use, measurement time, age of measurement.

#### 2.1.4 Evidence quality evaluation

We assessed the level of evidence for the selected studies according to the Oxford center for evidence-based medicine 2011 guidelines. (http://www.cebm.net/wp-content/uploads/2014/06/CEBM-Levels-of-Evidence-2.1.pdf.) For cohort and case-control studies, the methodological quality was evaluated using the Newcastle–Ottawa Scale (NOS) by 2 investigators independently (22). It has been recognized as a validated quality assessment instrument for nonrandomized trials. The evaluation was based on three parameters: 1) selection of the study population; 2) comparability between the groups; and 3) ascertainment of the exposure or outcome. In the case of disagreements, a third investigator adjudicated. The detailed scoring criteria are shown in Supplementary Tables S2, 3.

### 2.2 Mendelian randomization analyses

#### 2.2.1 Data sources

Mendelian randomization is an analytical method to assess the causality of associations between observed exposures or risk factors and clinically relevant outcomes (23). The MR study was based on the following assumptions: (1) strongly associated with risk factors; (2) not associated with confounders; and (3) instrumental variables affecting outcomes only through exposure factors rather than direct effects. Exposure factors (GWAS for preterm delivery) in this study were obtained from the Early Growth Genetics Consortium (EGG database, and cortisone levels were obtained from published GWAS studies (Cortisone) (24, 25). Cortisone was used as a proxy for cortisol regulation due to the limited availability of cortisol-specific GWAS data. Detailed data sources are provided in Supplementary Table S4. All data were obtained from public databases, and no ethical issues were involved.

#### 2.2.2 Selection of instrumental variables

Single-nucleotide polymorphisms (SNPs) with *p*-values less than 5 × 10^−8^ were selected as instrumental variables. To ensure that SNPs are independent of each other and to limit the scope of the analysis, the linkage disequilibrium (LD) was set with a threshold of r^2^ < 0.001, and the physical distance was set at 10,000 kb to avoid bias. The F-statistic value of each SNP was more than 10 to maximize the exclusion of weak instrumental variables to ensure strong correlations between instrumental variables and exposure.

### 2.3 Statistical analysis

All meta-analyses and Mendelian randomization (MR) analyses were performed using R software (version 4.4.1). The standardized mean difference (SMD) was selected as the effect size measure for cortisol levels. The significance level for the meta-analysis was set at *α* = 0.05. The outcome (cortisol levels) was analyzed as a continuous variable. Where necessary, standard errors were converted to standard deviations for consistency based on the available data. If a study reported multiple groups, data were combined, and analyses were performed using the combined data. I^2^ testing was used to assess the magnitude of the heterogeneity among studies (26). If there was no significant heterogeneity in the studies (I^2^ ≤ 50%), a fixed-effects model was used. Conversely (I^2^ > 50%), a random-effects model was used (27). Considering the inter-study variations including differences in specimen type, measurement ages, gestational ages, measurement times, the use of steroid hormones and measurement methods, subgroup analyses were performed to determine potential sources of heterogeneity. Forest plots were generated for each meta-analysis to illustrate the effect sizes and confidence intervals of each study and the pooled effect size. To further explore heterogeneity, univariate meta-regression was performed including a single covariate; then, an adjusted multivariable model was performed including factors with *p* < 0.1 in the univariate analysis. Funnel plots and Egger's test were used to estimate publication bias (28). If significant publication bias was detected, a trim-and-fill method was performed to examine whether studies should be imputed to make the results more symmetrical to minimize the effects of publication bias. And then we used MR analysis to investigate the causal effect of gestational ages at birth on cortisone levels. Univariate analyses were mainly performed using the inverse variance weighted (IVW) method (29), with MR Egger regression (30), weighted median (WM), simple mode (SM), and weighted mode methods used as secondary MR analysis methods to explore causal correlations. Heterogeneity was tested using Cochran's *Q* test, and pleiotropy was tested with the MR-Egger test. Sensitivity analyses were performed using the “leave-one-out” method in meta-analyses and MR analyses. This method evaluates the stability of the results by excluding one study each time, and assessing whether the results are strongly influenced by a single study. All MR analyses were performed using the TwoSampleMR and MRPRESSO packages, while meta-analyses were carried out with the meta package.

## 3 Results

### 3.1 Basic characteristics of the included literature

**Figure 1:**
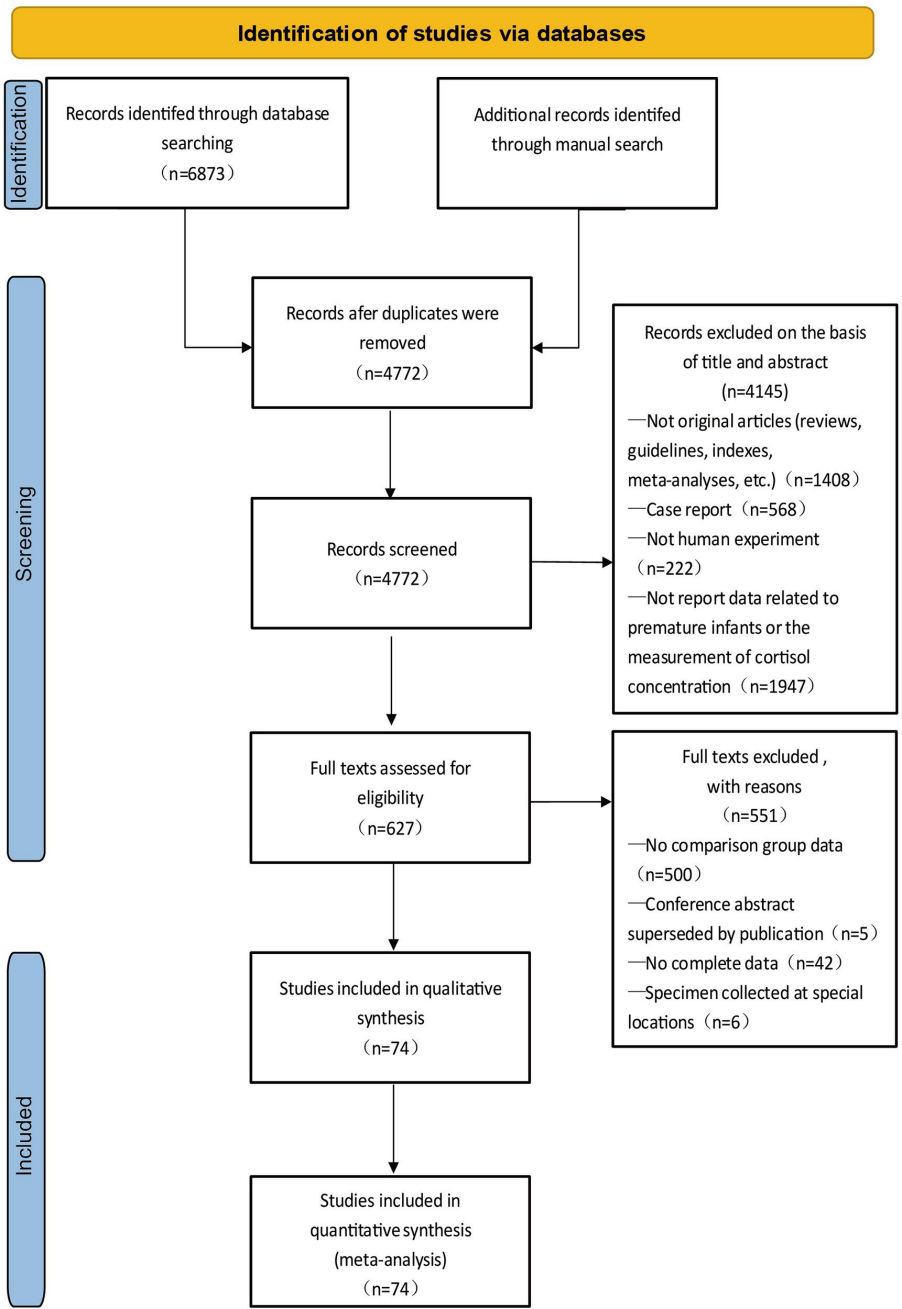
Flow diagram of the selection process of included articles.

According to the search strategy, a total of 6,873 relevant articles were retrieved, including 6,014 articles from English databases (244 articles from Pubmed, 2,864 articles from EMBASE, 571 articles from Web of Science, 199 articles from Cochrane Library, 1,420 articles from Scopus, 716 articles from ScienceDirect) and 859 articles from Chinese databases (407 articles from CNKI, 120 articles from CBM, 212 articles from Wan Fang database, 120 articles from VIP). We used EndNoteX9 software to exclude 2,101 duplicate articles. According to the inclusion and exclusion criteria, 74 studies were finally included after carefully reading the titles, abstracts, and full texts. Six articles collecting samples from skin, hair, and urine were excluded during this process, considering the reasons for the sample collection sites. In the end, 74 articles were included, with samples collected from saliva (31 articles), peripheral blood (28 articles), and UCB (18 articles) [two articles collected peripheral blood and UCB samples (7, 8); one article collected both saliva and peripheral blood samples (31)]. The literature screening process and results are shown in Figure 1. The 74 studies selected were published from 1974 to 2024, and the basic characteristics of the included studies are shown in Supplementary Table S5.

### 3.2 Quality evaluation of included literature

The 74 studies are generally of overall high quality and low risk of bias. According to the Oxford Center for Evidence-Based Medicine 2011 guidelines, this study included 49 low-quality cohort studies, case-control studies, or case series (level 4) and 25 prospective follow-up studies (level 3). The quality of the included literature was evaluated according to the NOS scoring scale (Supplementary Tables S2–4), with a score of 5–8. The NOS scores were divided into three levels: low (< 5 points), medium (5–7 points), and high quality (≥ 8 points). The specific evaluation results are shown in Supplementary Table S6.

### 3.3 Results of meta-analyses

#### 3.3.1 Comparison of cortisol level between preterm and term infants

A meta-analysis of cortisol levels in preterm and term infants was conducted based on the 74 included studies. The results indicated that there was no statistically significant difference between the two groups [standardized mean difference (SMD), −0.07; 95% CI, −0.21 to 0.08, *P* = 0.368] (Supplementary Figure S1). We observed that cortisol levels were measured using different methods across the 74 studies, including radioimmunoassay (RIA), enzyme-Linked Immunosorbent assay (ELISA), fluorescence immunoassay (FIA), and liquid chromatography-tandem mass spectrometry (LC-MS/MS). Therefore, a further subgroup analysis was conducted based on the measurement method, and no significant differences were observed (Supplementary Figure S2). There existed significant heterogeneity (I^2^ = 87.1%, *p* < 0.001) among the included studies. It is speculated that this heterogeneity may arise from differences in specimen collection sites across the 74 studies. Meanwhile, we recognized that there were significant differences in the age of premature infants included in the studies. UCB was collected immediately after cord clamping at birth, while the majority of peripheral blood samples were obtained during the early postnatal stage. Moreover, when it comes to studies that measure salivary cortisol levels, the age span of premature in studies was mostly from 2 months to adulthood. To explore the source of this heterogeneity, we investigated whether there were differences in cortisol levels between the two groups in UCB, peripheral blood, and saliva, following the chronological order of sample collection age.

#### 3.3.2 Comparison of UCB cortisol between groups

**Figure 2:**
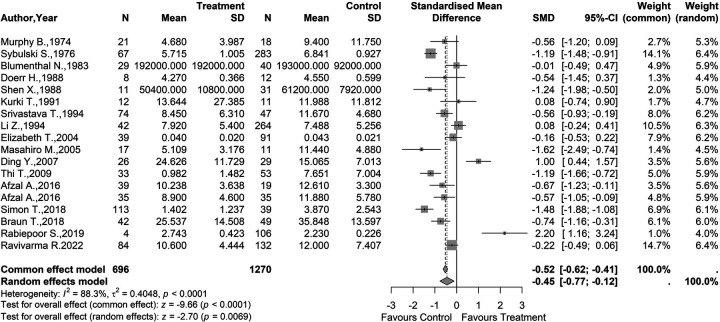
Forest plot for umbilical cord blood (UCB) cortisol levels in preterm compared to the term infants.

We found that 18 studies measured UCB cortisol levels among 74 studies, including 696 preterm infants and 1,270 term infants (7, 8, 32–47). There was heterogeneity (I^2^ = 88.3%, *p* < 0.001) among these studies, so a random effects model was used. The results were as anticipated. Figure 2 shows that UCB cortisol levels in premature infants at birth were significantly lower than those in term infants [SMD, −0.45; 95% CI, −0.77 to −0.12, *P* = 0.007]. To clarify whether gestational age can affect cortisol levels, we divided the enrolled newborns into two groups: very preterm (GA < 32 weeks) and late or moderately preterm (32 weeks ≤ GA ≤ 37weeks) according to gestational age. The meta-analysis showed that UCB cortisol levels were lower in both groups compared with term infants, as we expected (Supplementary Figure S3). Likewise, a similar result was found in subgroups in terms of corticosteroids prenatally and postnatally (Supplementary Figures S4, 5). In the subgroup analysis stratified by measurement method, only LC-MS/MS showed a significant difference (Supplementary Figure S6).

#### 3.3.3 Comparison of peripheral blood cortisol between groups

**Figure 3:**
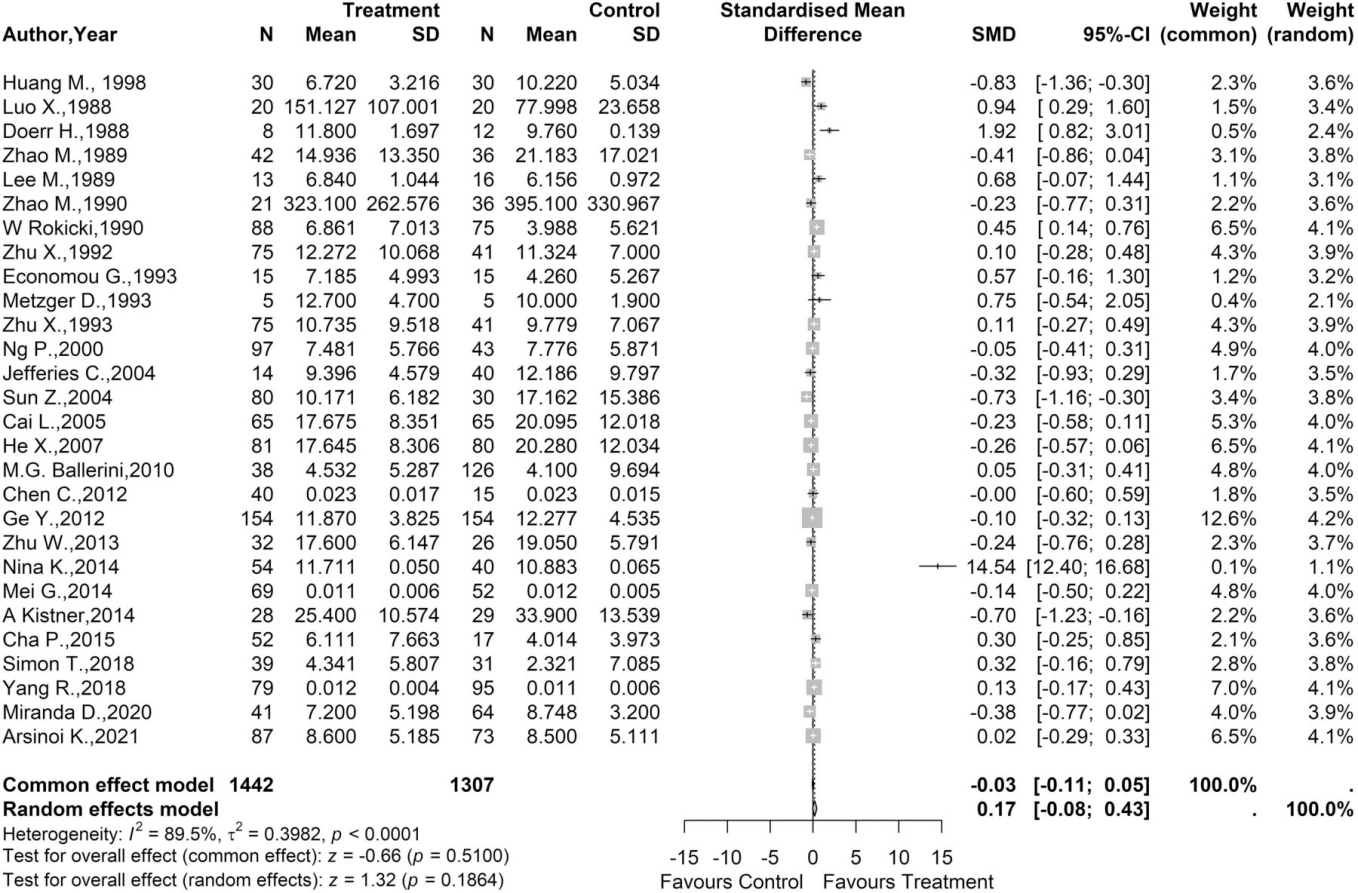
Forest plot for peripheral blood cortisol levels in preterm compared to the term infants.

We performed a meta-analysis of 28 studies with peripheral blood cortisol levels, involving 1,442 premature infants and 1,307 term infants (7, 8, 31, 48–72). According to the results of the heterogeneity test (I^2^ = 89.5%, *p* < 0.001), the random effect model was chosen to estimate the SMD. To our surprise, there were no distinct differences in cortisol levels between premature and term infants [SMD, 0.17; 95% CI, −0.08 to 0.43, *P* = 0.186] (Figure 3).

**Figure 4:**
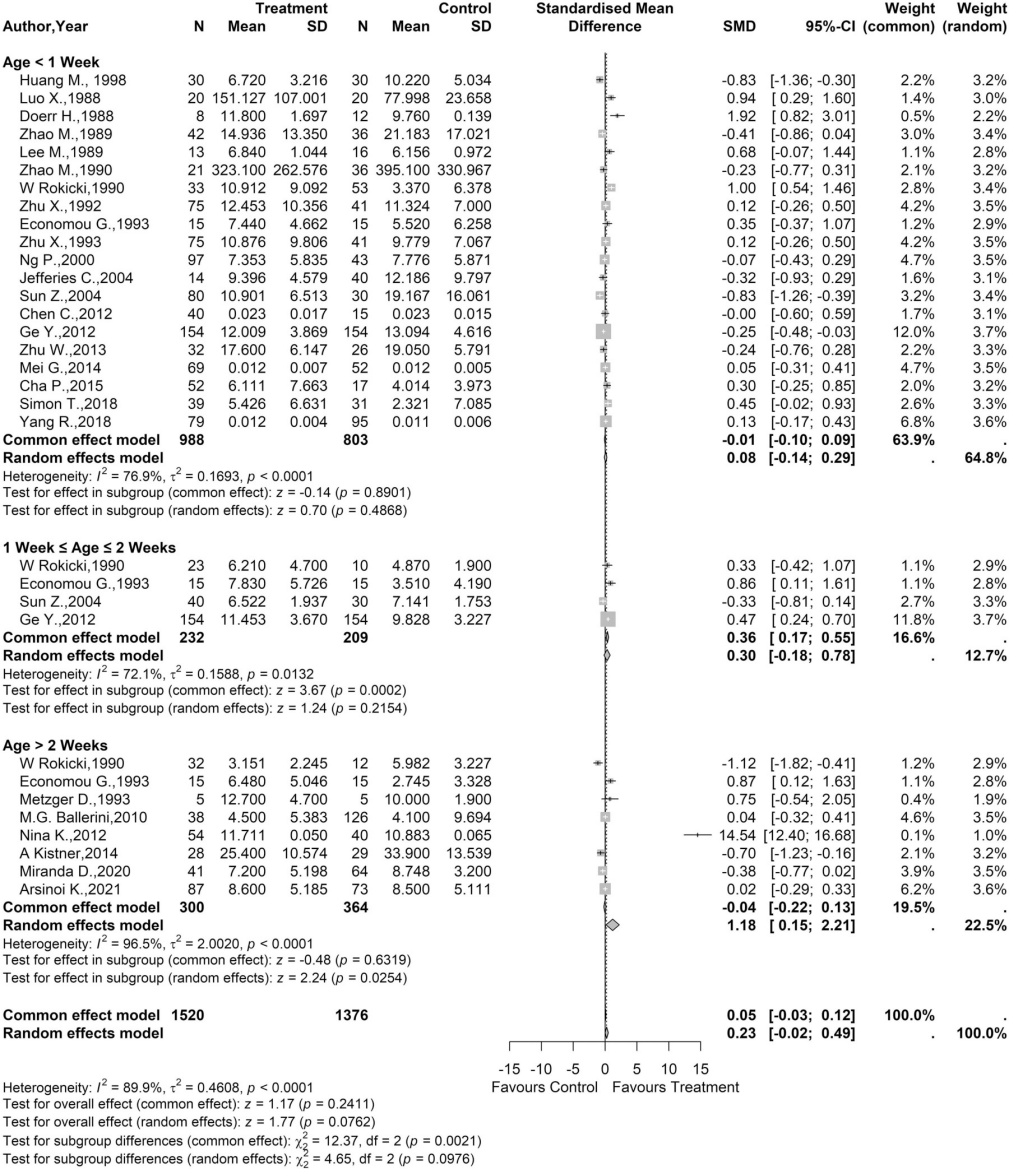
Subgroup meta-analysis of peripheral blood corsticol levels by age.

**Figure 5:**
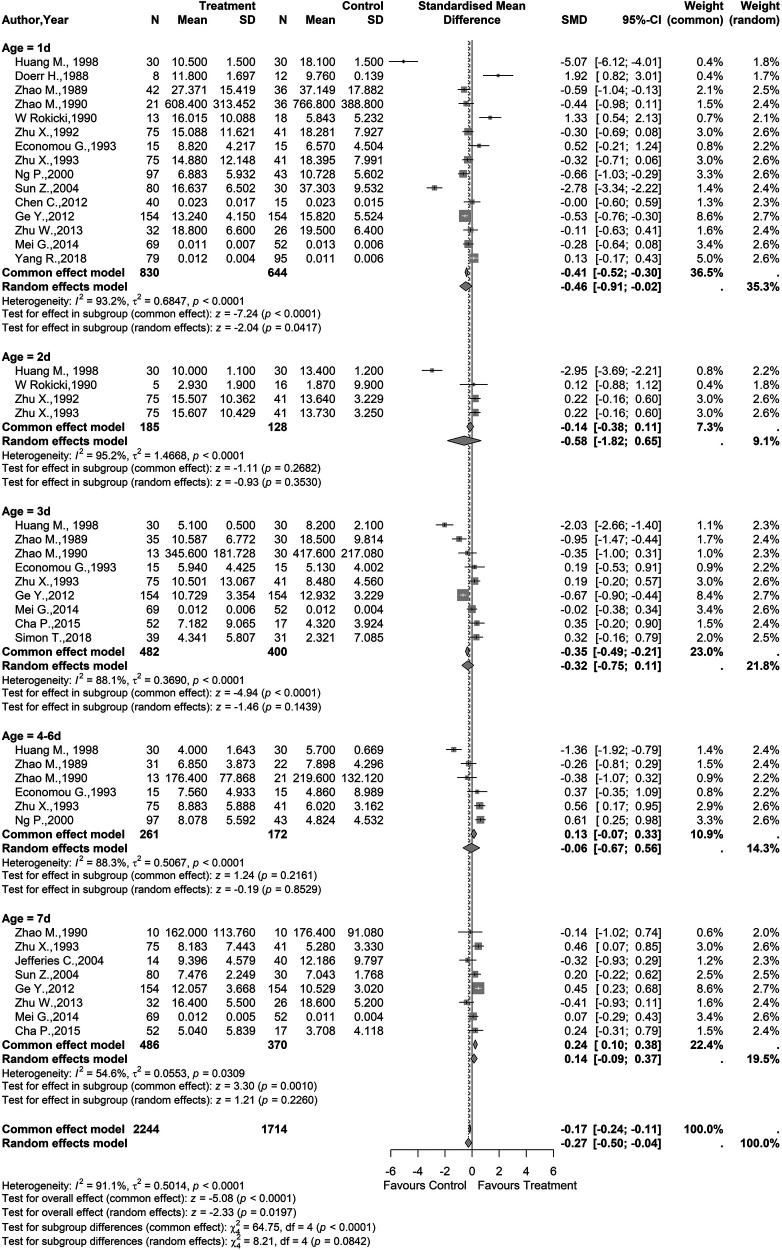
Subgroup analysis of peripheral blood corsticol levels within the first week.

Previous studies have demonstrated that the function of the HPA axis matures gradually with age. Subgroup analysis was conducted based on the age at measurement. There were no statistically significant differences in cortisol levels between the two groups during the first week [SMD, 0.08; 95% CI, −0.14 to 0.29, *P* = 0.487] and the second week of life [SMD, 0.30 95% CI, −0.18 to 0.78, *P* = 0.215] (Figure 4). However, meta-analysis results showed that cortisol levels were higher in premature infants compared to term infants after 2 weeks [SMD, 1.18; 95% CI, 0.15 to 2.21, *P* = 0.025] (Figure 4). Postnatally, if placed under excessive stress, preterm infants may have inadequate adrenal cortisol production due to their immature HPA axis and adrenocortical function, which makes them more prone to developing relative adrenal insufficiency (RAI). It is reported that RAI mostly occurs within the first week of life. We subdivided measurement age into 1d, 2d, 3d, 4–6d, and 7d after birth to further investigate changes in cortisol levels during this period. The meta-analysis revealed that the cortisol level in the peripheral blood of premature infants was lower than that of term infants on the first day of life [SMD, −0.46; 95% CI, −0.91 to −0.02, *P* = 0.042], which is consistent with UCB results (Figure 5). There were no significant statistical differences observed at other ages (Figure 5). However, when combined with meta-analysis results after two weeks, it is encouraging and uplifting that cortisol levels in premature infants showed a trend of being initially lower and then higher compared to term infants. Besides measurement age, the GA, measurement times, the usage of steroid hormones and measurement methods affect cortisol levels. Further subgroup analysis based on these influencing factors did not reveal statistical differences (Supplementary Figures S7–11).

#### 3.3.4 Comparison of salivary cortisol between groups

A total of 31 studies collected saliva samples, including 1,914 premature infants and 1,054 healthy term infants (10–14, 31, 73–97). A random-effects model was used because of significant heterogeneity (I^2^ = 76.4%, *p* < 0.001). There was no statistically significant difference between two groups [SMD, −0.04; 95% CI, −0.21 to 0.13, *P* = 0.628] (Supplementary Figure S12). Subgroup analysis based on measurement age (within 1 year and after 1 year) did not reveal statistical differences (Supplementary Figure S13). Unlike peripheral blood samples, salivary cortisol samples were collected at later time points. Further subdivision of these samples into groups of ≤3 months, 3∼6 months, 6∼8 months, ≥8 months infancy and toddlerhood, preschool and school age, adolescence and adulthood also showed no statistically significant differences (Supplementary Figures S14, 15). Other subgroup analyses also revealed no statistically significant differences (Supplementary Figures S16–20).

### 3.4 Meta-regression

Given the high level of heterogeneity observed in the meta-analysis, univariate and multivariate meta-regression analyses were performed to explore potential variables contributing to the heterogeneity. These analyses included specimen type, measurement methods, prenatal and postnatal corticosteroid use, and ages. The univariate meta-regression results showed that specimen type (QM = 10.102, df = 2, *P* = 0.0064) and prenatal corticosteroid use (QM = 8.517, df = 3, *P* = 0.0365) were statistically significant moderators. They explained 7.60% and 3.75% of the heterogeneity, respectively. To investigate potential moderators of the effect size, variables with *P* < 0.1 in the univariate analyses were included to construct a multivariate meta-regression model. The overall test was significant (QM = 14.87, df = 6, *p* = 0.0213), and the model collectively explained 4.45% of the heterogeneity (R^2^ = 4.45%). However, significant residual heterogeneity remained (QE = 507.86, df = 70, *p* < 0.0001, I^2^ = 86.22%), indicating that other important moderators were not included in the present study (Supplementary Tables S7, 8).

### 3.5 Publication bias analysis and sensitivity analysis

Publication bias was assessed by the funnel plots and Egger's test. The results showed that there was a publication bias in the meta-analysis (t = 2.55, *p* = 0.015, Supplementary Figure S21). The results of Egger's test didn't reveal any evidence of publication bias for UCB cortisol (t = 0.67, *p* = 0.512) and salivary cortisol (t = 1.16, *p* = 0.254). The funnel plots were basically symmetrical (Supplementary Figures S22, 23). However, for peripheral blood cortisol, significant publication bias was detected by Egger's test (t = 2.70, *p* = 0.012). Simultaneously, the asymmetrical pattern of the funnel plot (Supplementary Figure S24) for this outcome also raised suspicion of publication bias. The negative results might be unpublished. The Four studies were imputed using the trim-and-fill method to correct the asymmetry in the funnel plot (Supplementary Figure S25). After imputation the adjusted effect size was −0.08 [95% CI: −0.40 to 0.24, *p* = 0.624, I^2^ = 93.3%]. Trim-and-fill analysis showed that four studies need to be imputed to make the results more symmetrical. After performing the trim-and-fill method, no significant difference in cortisol levels was observed between preterm and term infants, indicating that the stability of our meta-analysis results was not affected. Subsequently, we performed sensitivity analysis using the leave-one-out method by calculating SMD of the remaining data after removal of one single study each time. The results were mostly consistent with the original results (Supplementary Figures S26–29). This indicates that the conclusion of this study are of robustness.

### 3.6 Results of MR analyses

IVW results showed a negative causal effect between preterm labor and cortisone levels (*β* = −0.011, 95% CI, −0.021 to 0.001, *P* = 0.035) (Supplementary Table S9). No significant heterogeneity was detected using Cochran's Q (Supplementary Figure S30). The intercepts of the MR-Egger regressions were all close to 0, indicating that there was no horizontal pleiotropy (Supplementary Figure S31). In the sensitivity analysis, the results of the “leave-one-out” method showed that eliminating any of the SNPs did not significantly affect the estimation of the causal correlation (Supplementary Figure S32), suggesting that the results of the MR analysis were robust.

## 4 Discussion

This systematic review and meta-analysis, complemented by Mendelian randomization, provides a comprehensive comparison of cortisol levels between preterm and term infants. Our main findings indicate that preterm infants have lower cortisol levels at birth and on the first day, but thereafter exhibit a rapid rise, surpassing term infants by two weeks of age (Supplementary Figure S33). In the long term, however, salivary cortisol levels are comparable between groups. MR analysis supports a causal relationship between preterm birth and cortisone, suggesting genetically influenced alterations in cortisol metabolism.

Cortisol is a glucocorticoid produced by the adrenal glands and helps regulate metabolism (98, 99). In early pregnancy, fetal cortisol is derived from progesterone in the placenta. The fetus lacks the key enzyme for cortisol synthesis, 3β-hydroxysteroid dehydrogenase (3β-HSD), which is not produced until 23 weeks of gestation and not significantly synthesised until 30 weeks (100). Additionally, elevated activity of placental 11β-hydroxysteroid dehydrogenase type 2 (11β-HSD2) in early gestation may exert an additional effect of limiting fetal exposure to bioactive cortisol. This placental enzyme exerts a protective role for the developing fetus by oxidizing active cortisol to its inactive metabolite, thereby preventing excessive maternal cortisol from reaching the fetal circulation (101). Notably, 11β-HSD2 is highly expressed during the second trimester and declines sharply in the third trimester to prepare for birth. This could explain why the levels of cortisol in preterm infants are lower than those in term infants at the beginning of life due to a lack of 3β-HSD, high expression of 11β-HSD2, and the immaturity of the preterm infant's HPA axis (102). Maternal antenatal corticosteroid administration is a common clinical intervention to promote fetal lung maturation (103). Synthetic glucocorticoids readily cross the placental barrier and enter the fetal circulation, exerting negative feedback on the fetal HPA axis. This directly reduces the synthesis and release of cortisol from the fetal adrenal cortex, thereby suppressing endogenous cortisol production (104). Subgroup analysis in our study (Supplementary Figure S3) revealed that cortisol levels were significantly lower in preterm infants compared to term infants, regardless of antenatal corticosteroid exposure. However, the magnitude of this reduction was greater in the subgroup of preterm infants exposed to antenatal steroids, suggesting a partial inhibitory effect of antenatal corticosteroids. These findings indicate that while antenatal steroids contribute to lower cortisol levels at birth, the inherent immaturity of the HPA axis remains the predominant factor. Studies suggest antenatal corticosteroids can exert programming effects on the HPA axis (105). They may influence the fetus's long-term stress response. However, the persistence of this effect is inconclusive. In our study, no significant differences in cortisol levels (in saliva and blood samples) were observed, consistent with previous research (106).

The physiological stress response is regulated mainly by the hypothalamus-pituitary-adrenal corte*x* axis (HPA axis). When the body is under stress, CRH is released, leading to the release of ACTH, which then stimulates the adrenal gland to secrete cortisol. Understanding this stress-response axis is crucial for interpreting the dynamic changes of cortisol in preterm infants. P C Ng et al. conducted hCRH testing on premature infants and discovered that the pituitary gland exhibits a strong response to exogenous stress, suggesting that it may already be functionally mature in the early stages of human development (107). In most cases, the adrenal glands largely recover function by the second week after birth (108). In our study, there was no significant difference observed in cortisol levels between premature and term infants as early as postnatal day 2. This suggests that the HPA axis of premature infants may mature earlier in response to stress, indirectly indicating that premature infants face more environmental stressors. Additionally, this study is the first to uncover the dynamic changes in cortisol levels among premature infants. As adrenal function gradually recovers and stress accumulates, cortisol levels in premature infants continue to rise, surpassing those of term infants within two weeks of birth. After birth, loss of placental 11β-HSD2, together with adrenal maturation and repeated stress exposures (e.g., procedural pain, respiratory support), likely drives the rapid increase in cortisol levels seen by two weeks (109). This “stress hypercortisolism” may represent a compensatory adaptation to extrauterine life but could also predispose to altered HPA programming and later metabolic or neurobehavioral outcomes (9). The probable explanation may be attributed to the fetal origins hypothesis: namely, compensatory adaptations that occur after exposure to suboptimal environments result in apparent short-term beneficial effects (110, 111). Another possible cause was exposure to stress over a long period causes the HPA axis to become hyperactive, elevating plasma corticosteroids, including cortisol (13, 112).

In our study, salivary cortisol levels did not differ between preterm and term infants in the long term. As we all know that salivary cortisol can reflect the level of biologically active free cortisol in the blood (113). Measuring cortisol concentrations in saliva is even considered a better method to assess adrenocortical function than in serum (114). The long-term salivary cortisol levels in the two groups tended to converge, which may suggest that the function of the hypothalamic-pituitary-adrenal (HPA) axis in preterm infants ultimately achieved adaptive regulation. Alternatively, it may indicate that the elevated cortisol levels observed in the early postnatal period were merely a transient physiological phenomenon. Furthermore, methodological factors related to the assay, such as sampling conditions and differences in detection reagents, may have attenuated the actual differences in salivary cortisol levels between the groups. Currently, there is no relevant research exploring the long-term changes in cortisol levels among premature infants. Existing meta-analyses primarily focus on cortisol levels in young infants and have not examined the impact of gestational age at birth on the HPA axis (115). Nia Fogelman et al. indicated no significant correlation between early life stress and baseline cortisol. They discovered if blood samples were collected, the early life stress was associated with a blunting effect of cortisol (116). Blood sampling itself is an influencing factor; the different ability of PI and TI to respond to stress may have influenced the results.

The results of the Mendelian randomization analysis in this study further provide genetic evidence for the underlying mechanisms. In our study, we identified a negative causal effect of preterm birth on cortisone levels. This result is consistent with previous reports showing elevated glucocorticoid precursor levels in preterm infants (117). Cortisone, which is the precursor of cortisol, is also a hormone involved in the body's stress response. The 11 beta- HSD2 can play a crucial role in converting hormonally active cortisol to inactive cortisone (15, 118, 119). Preterm infants lack the enzymes required for glucocorticoid synthesis and exhibit impaired 11β-HSD activity, as well as decreased CYP11B1 activity. This may explain the accumulation of cortisol precursors and the limitation of GC production (7). Studies have shown that the conversion between cortisol and cortisone in preterm infants tends to favor cortisone production (120), further supporting this conclusion. This suggests that preterm birth may lead to persistent alterations in the cortisol-cortisone conversion process. Elevation of cortisol precursors reflects adrenal immaturity, suggesting that these infants may have difficulty meeting physiological cortisol demands through the HPA axis, thereby increasing the risk of adrenal crisis (17). Evidence indicates that preterm birth may exert a lasting programming effect on the HPA axis (121). In early life, this effect manifests as an inability to secrete sufficient cortisol levels in response to stress. With increasing age, the axis becomes progressively more active, which further supports the dynamic changes in cortisol levels observed in our meta-analysis. This genetic evidence complements the observational meta-analysis and underscores a biological link between preterm birth and glucocorticoid regulation. Clinically, in addition to cortisol, measuring cortisone levels or the cortisol/cortisone ratio may contribute to a more comprehensive assessment of HPA axis function and glucocorticoid activity.

However, this study has the following limitations. First, this meta-analysis had some publication bias. Second, during subgroup analysis, only a single factor was considered at a time, ignoring the influence of potential confounders. For instance, when exploring the measurement age, we were unable to further stratify the studies by gestational age because of the limited sample size and the small number of relevant research studies. This lack of stratification might have introduced bias, as different GAs could potentially modify the relationship between cortisol levels and the variables of interest. Furthermore, the MR analysis relies on available GWAS data; cortisone was used as a proxy for cortisol due to data availability. Although cortisone and cortisol are structurally similar and both belong to glucocorticoids, they are not biologically equivalent. Therefore, the causal inferences derived from MR analysis can only reflect the impact of preterm birth on cortisone metabolism, rather than a direct effect on cortisol metabolism. Future MR studies using direct cortisol GWAS data are warranted. Additionally, residual confounding in observational studies and pleiotropy in MR cannot be fully excluded. Third, despite performing subgroup analyses and meta-regression, significant heterogeneity remained unexplained. This likely reflects the retrospective design of the included studies, as well as unmeasured or unreported factors, such as diurnal variation in sampling time, acute stress at sampling, and differences in study design or population characteristics. We could not fully include or adjust for all potential moderators that might affect heterogeneity. We could not fully include or adjust for all potential moderators that might affect heterogeneity. Therefore, more rigorous, prospective, large-sample, multicenter studies are still needed to validate these findings and provide evidence for clinical circulation management of preterm infants.

Despite certain limitations, the dynamic pattern of cortisol levels in preterm infants revealed by this study holds profound clinical significance. Decisions regarding hydrocortisone use should remain individualized, considering postnatal age, clinical status, and evidence of adrenal insufficiency, ideally guided by future randomized controlled trials. For preterm infants who have received antenatal glucocorticoid therapy, when they present with stress conditions such as hypotension, shock, or infection and show poor response to conventional treatment, cortisol levels should be assessed to be vigilant for RAI. If the baseline cortisol is too low or the cortisol increment following stress is insufficient, low-dose hydrocortisone replacement therapy may be considered. However, strict adherence to indications is required, and further research is needed to confirm its benefits. In other words, we can adopt a positive attitude while awaiting the maturation of the HPA axis and its stress response in preterm infants. Currently, there is no unified consensus regarding the dosage and duration of postnatal glucocorticoid use in preterm infants. In the future, multicenter prospective cohort studies are needed to dynamically monitor postnatal cortisol levels, clarifying the recovery pattern of HPA axis suppression. Randomized controlled trials should be designed to evaluate the efficacy and safety of cortisol treatment for RAI in preterm infants. Additionally, exploring cortisol levels as a biomarker for predicting the stress response capacity of preterm infants could guide individualized treatment. The implementation of these studies will contribute to optimizing glucocorticoid management strategies in the NICU for preterm infants.

In summary, we firstly found that preterm infants exhibit a distinct cortisol trajectory: initially lower, then higher than term peers, but eventually converging in the long term. Genetic data suggest that preterm birth may causally influence cortisone metabolism. These insights should inform cautious use of hydrocortisone in neonatal practice and stimulate further research into HPA axis development and stress programming in preterm infants.

## Data Availability

The original contributions presented in the study are included in the article/Supplementary Material, further inquiries can be directed to the corresponding author.
