# Supplementary material for: Differences in cortisol levels between preterm and term infants: a systematic review and meta-analysis combined with Mendelian randomization study
Source: Front Pediatr. 2026 Jun 2;14:1790465. doi: 10.3389/fped.2026.1790465 (PMC13269068; doi:10.3389/fped.2026.1790465)
Supplement: Supplementary file 1 [file Supplementaryfile1.docx]

Supplementary Material

# Supplementary Figures

##
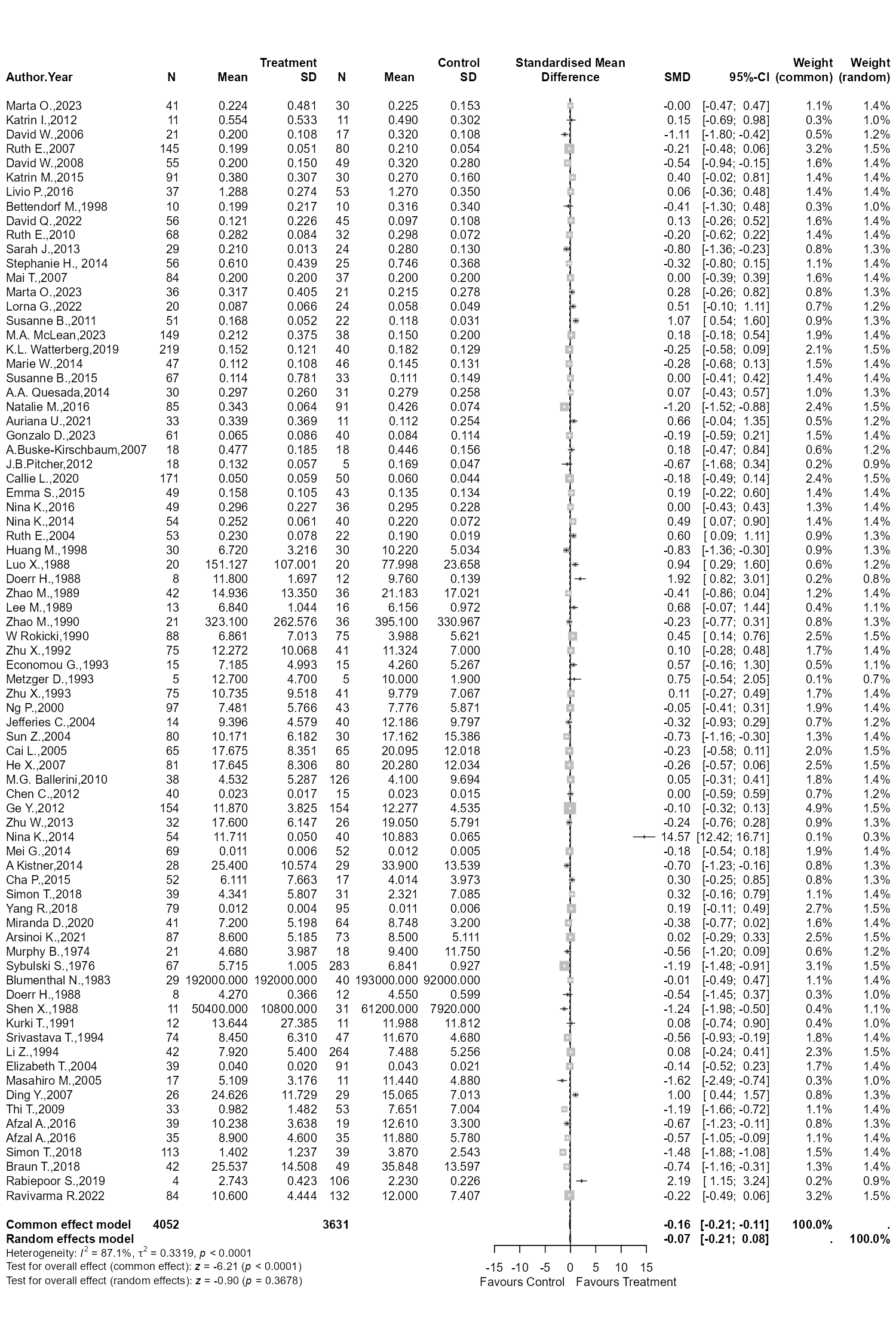


## Supplementary Figure 1. Forest plot comparing cortisol levels in the preterm group to the control group. The random-effects model showed a pooled SMD of -0.07 (95% CI: -0.21 to 0.08, p = 0.368), indicating no significant group difference. High heterogeneity was observed (I 2=87.1%, p < 0.001).


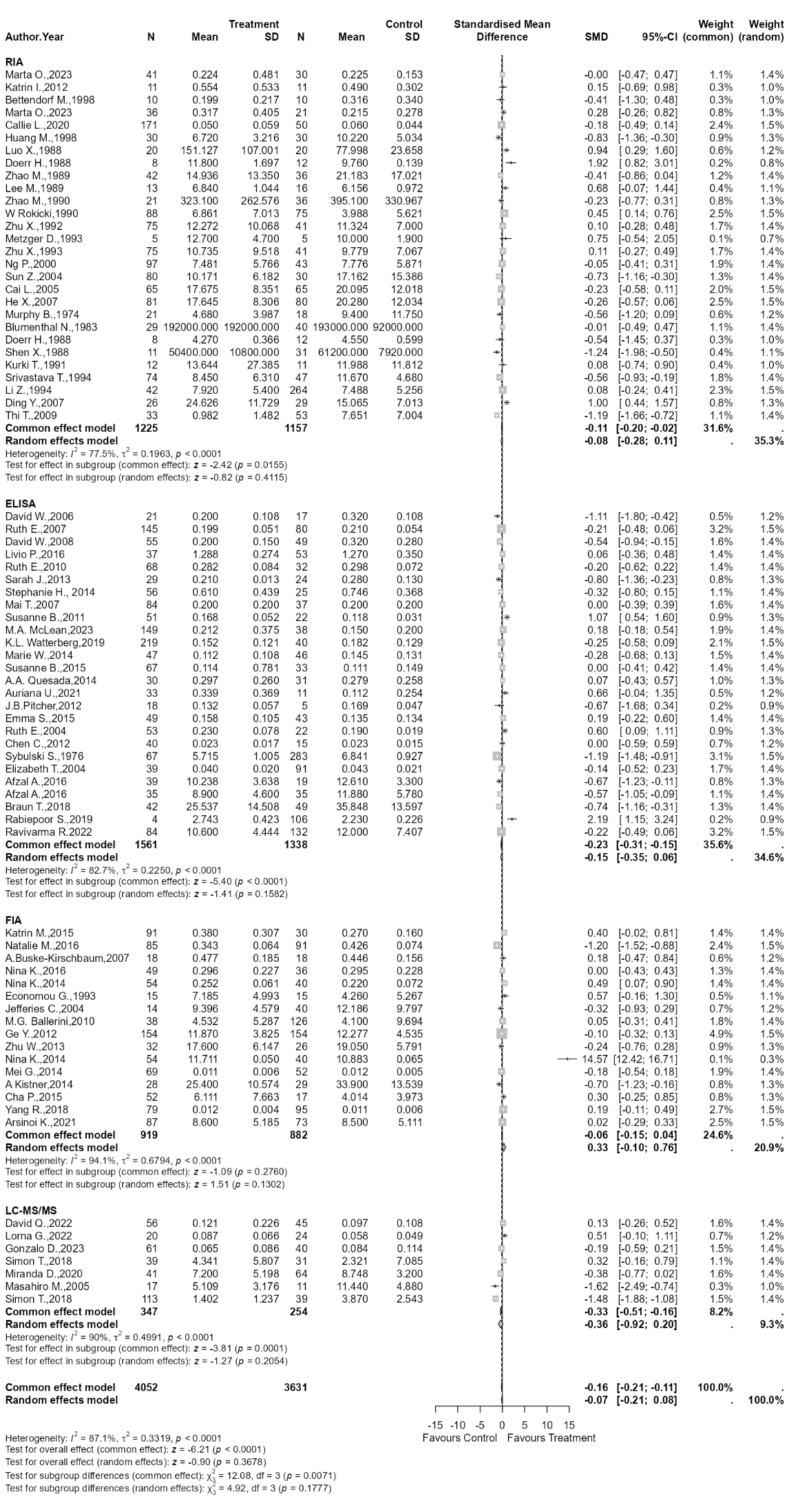


**Supplementary Figure 2.** Subgroup meta-analysis of cortisol levels by measurement methods. RIA subgroup: The random-effects model yielded a pooled SMD of -0.08 (95% CI: -0.28 to 0.11, p =0.412), with heterogeneity I2=77.5%. ELISA subgroup: The random-effects model yielded a pooled SMD of -0.15 (95% CI: -0.35 to 0.06, p =0.158), with heterogeneity I2 =82.7%. FIA subgroup: The random-effects model yielded a pooled SMD of 0.33 (95% CI: -0.10 to 0.76, p =0.130), with heterogeneity I2 =94.1%. LC-MS/MS subgroup: The random-effects model yielded a pooled SMD of -0.36 (95% CI: -0.92 to 0.20, p =0.205), with heterogeneity I2 =90.0%. Overall pooled result: The random-effects model showed a pooled SMD of -0.07 (95% CI: -0.21 to 0.08, p =0.368), with heterogeneity I2=87.1%. Consistently, the cortisol levels of preterm infants across different measurement methods showed no significant difference compared with term infants.


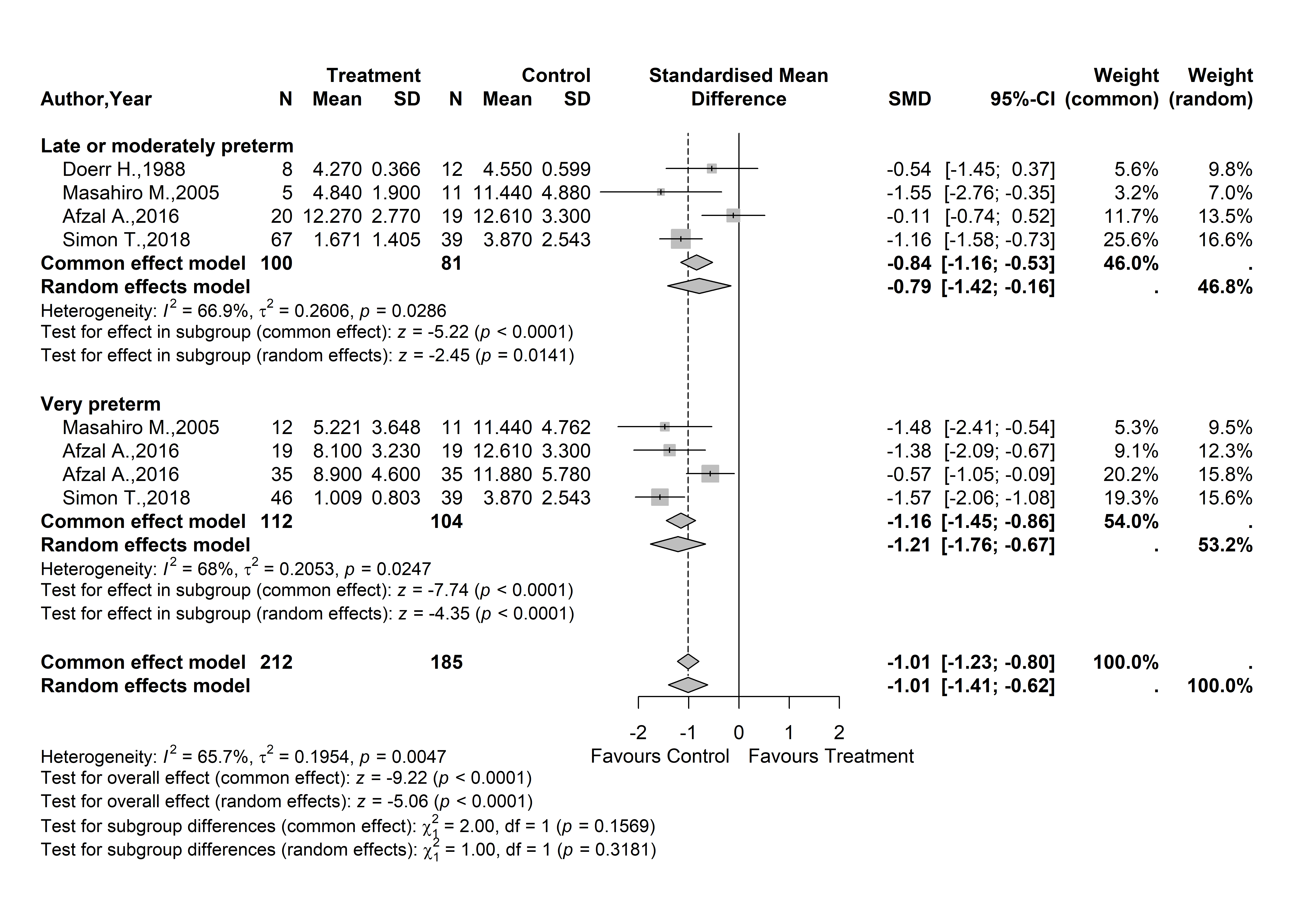


**Supplementary Figure 3.** Subgroup meta-analysis of umbilical cord blood(UCB) cortisol levels by gestational ages(GA). A total of 212 preterm infants and 185 term infants were included. Late or moderately preterm subgroup (n=100): The random-effects model yielded a pooled SMD of -0.79 (95% CI: -1.42 to -0.16, p =0.0141), with heterogeneity I^2^=68.9%. Very preterm subgroup (n=112): The random-effects model yielded a pooled SMD of -1.21 (95% CI: -1.76 to -0.67, p < 0.001), with heterogeneity I2 =68.9%. Overall pooled result: The random-effects model showed a pooled SMD of -1.01 (95% CI: -1.41 to -0.62, p < 0.001), with heterogeneity I^2^=65.7%.Consistently, the UCB cortisol levels of preterm infants across different GA were significantly lower than those of term infants.


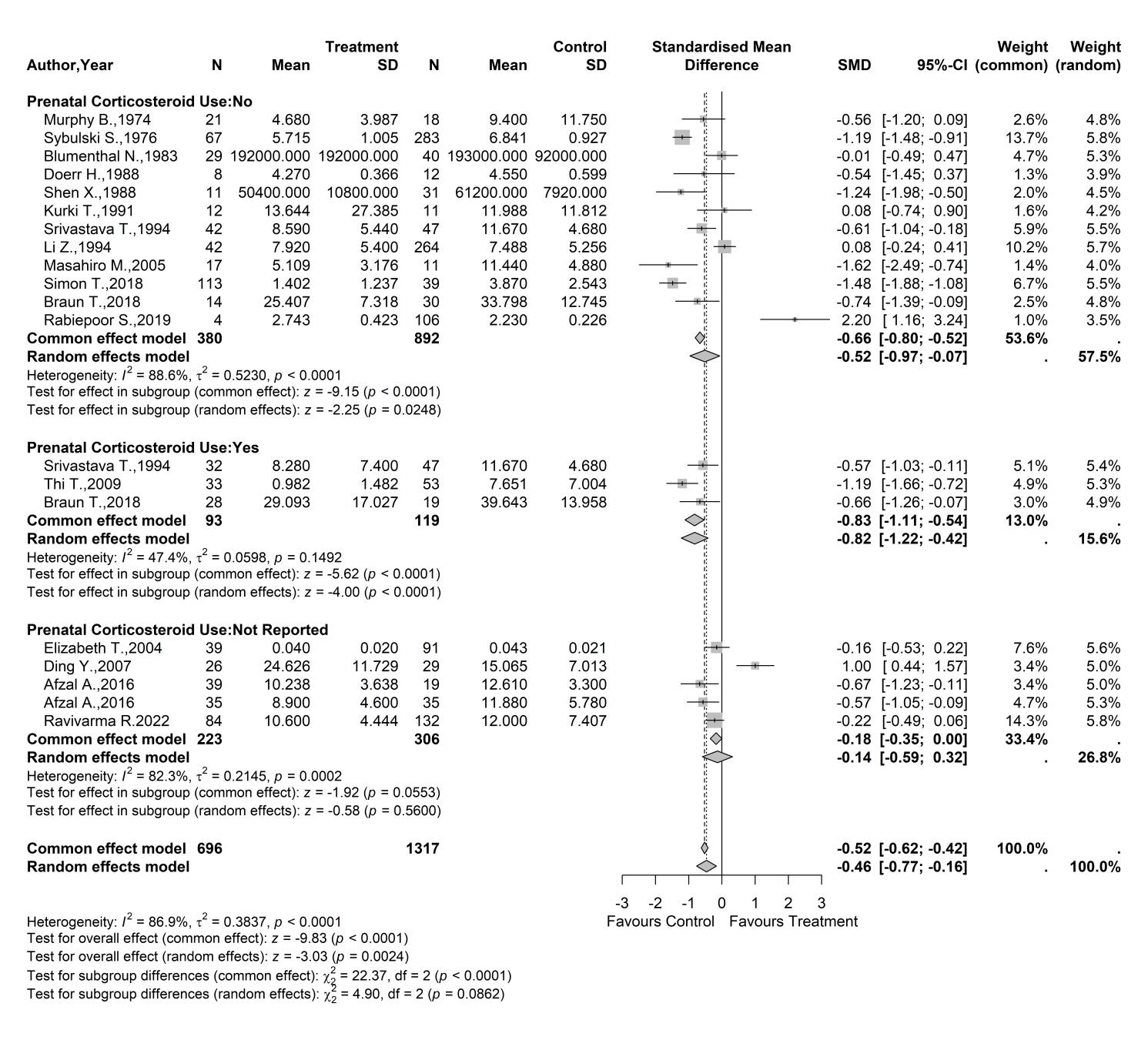


**Supplementary Figure 4.** Subgroup meta-analysis of UCB cortisol levels by the use of corticosteroids prenatally. A total of 696 preterm infants and 1317 term infants were included. Prenatal corticosteroid dont’t use subgroup (n=380): The random-effects model yielded a pooled SMD of -0.52 (95% CI: -0.97 to -0.07, p =0.0248), with heterogeneity I^2^=88.6%, suggesting lower cortisol levels in preterm infants. Prenatal corticosteroid use subgroup (n=93): The common-effects model yielded a pooled SMD of -0.83 (95% CI: -1.11 to -0.54, p < 0.001), with heterogeneity I^2^ =47.4%, indicating that the cortisol level of preterm infants was lower than that of full-term infants.. Prenatal corticosteroid didn’t report subgroup (n=223): The random-effects model yielded a pooled SMD of -0.14 (95% CI: -0.59 to 0.32, p =0.560), with heterogeneity I^2^=82.3%, indicating that there was no difference in cortisol levels between the two groups.


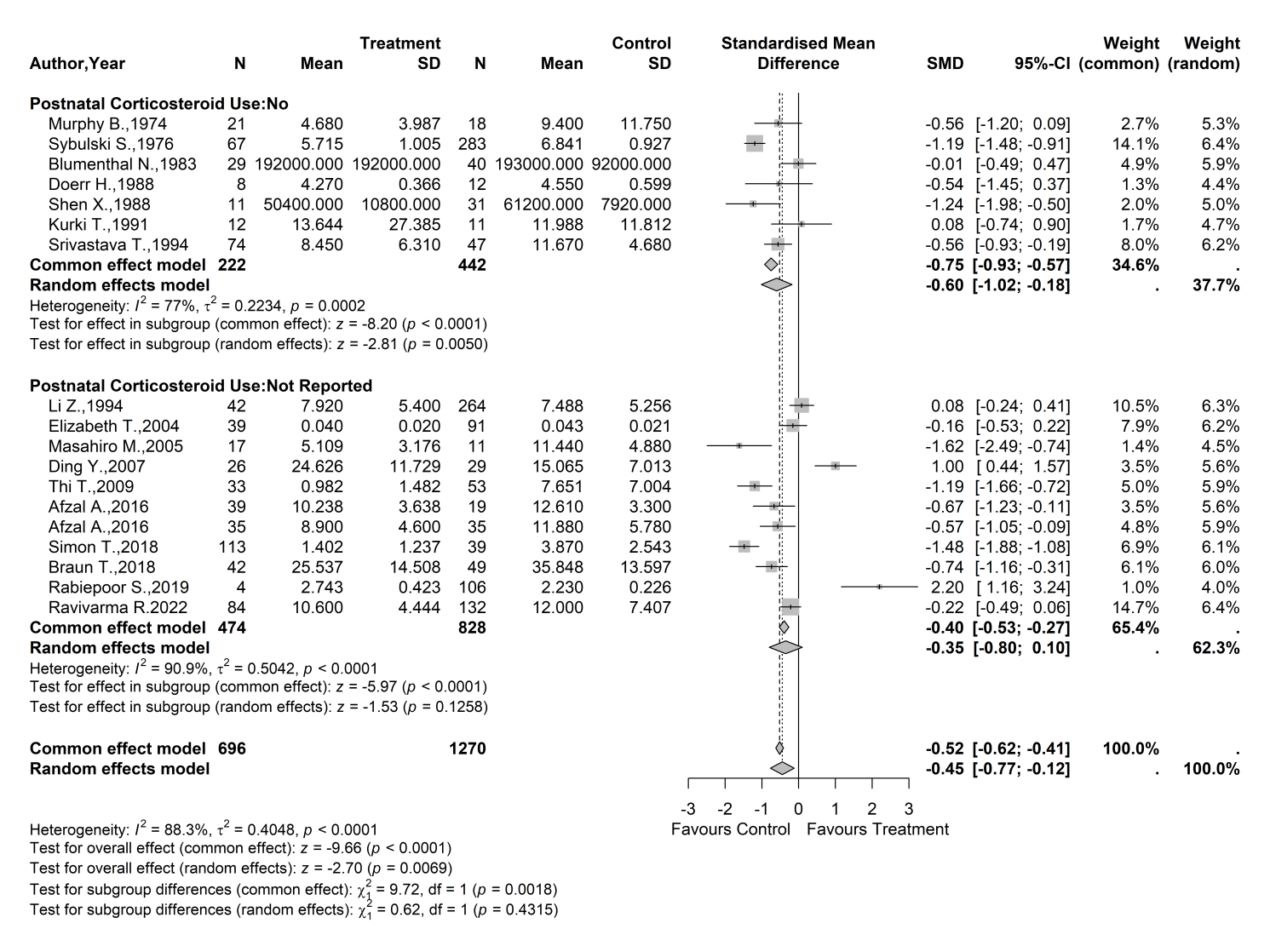


**Supplementary Figure 5.** Subgroup meta-analysis of UCB cortisol levels by the use of corticosteroids postnatally. A total of 696 preterm infants and 1270 term infants were included. Postnatal corticosteroid dont’t use subgroup (n=222): The random-effects model yielded a pooled SMD of -0.60 (95% CI: -1.02 to -0.18, p =0.0050), with heterogeneity I^2^=77.0%, suggesting lower cortisol levels in preterm infants. Postnatal corticosteroid did not report subgroup (n=474): The random-effects model yielded a pooled SMD of -0.35(95% CI: -0.80 to 0.10, p =0.560), with heterogeneity I^2^=90.9%, indicating that there was no difference between two groups.


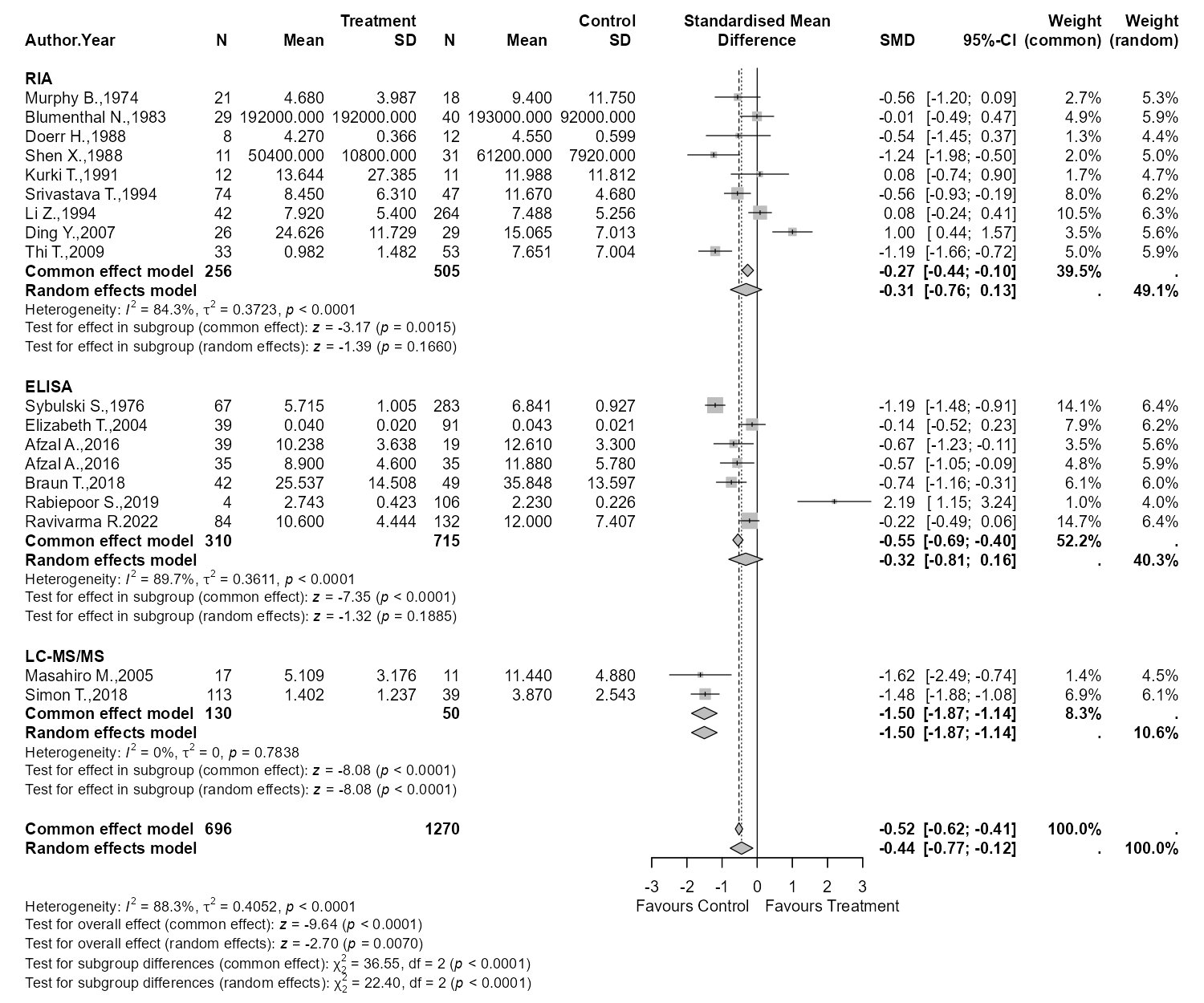


**Supplementary Figure 6.** Subgroup meta-analysis of umbilical cord blood(UCB) cortisol levels by measurement methods. RIA subgroup: The random-effects model yielded a pooled SMD of -0.31 (95% CI: -0.76 to 0.13, p =0.166), with heterogeneity I^2^=84.3%. ELISA subgroup: The random-effects model yielded a pooled SMD of -0.32 (95% CI: -0.81 to 0.16, p =0.188), with heterogeneity I^2^ =89.7%. LC-MS/MS subgroup: The random-effects model yielded a pooled SMD of -0.44 (95% CI: -0.77to -0.12, p < 0.001), with heterogeneity I2 =88.3%. Overall pooled result: The random-effects model showed a pooled SMD of -0.44 (95% CI: -0.77 to -0.12, p =0.000), with heterogeneity I^2^=88.3%.


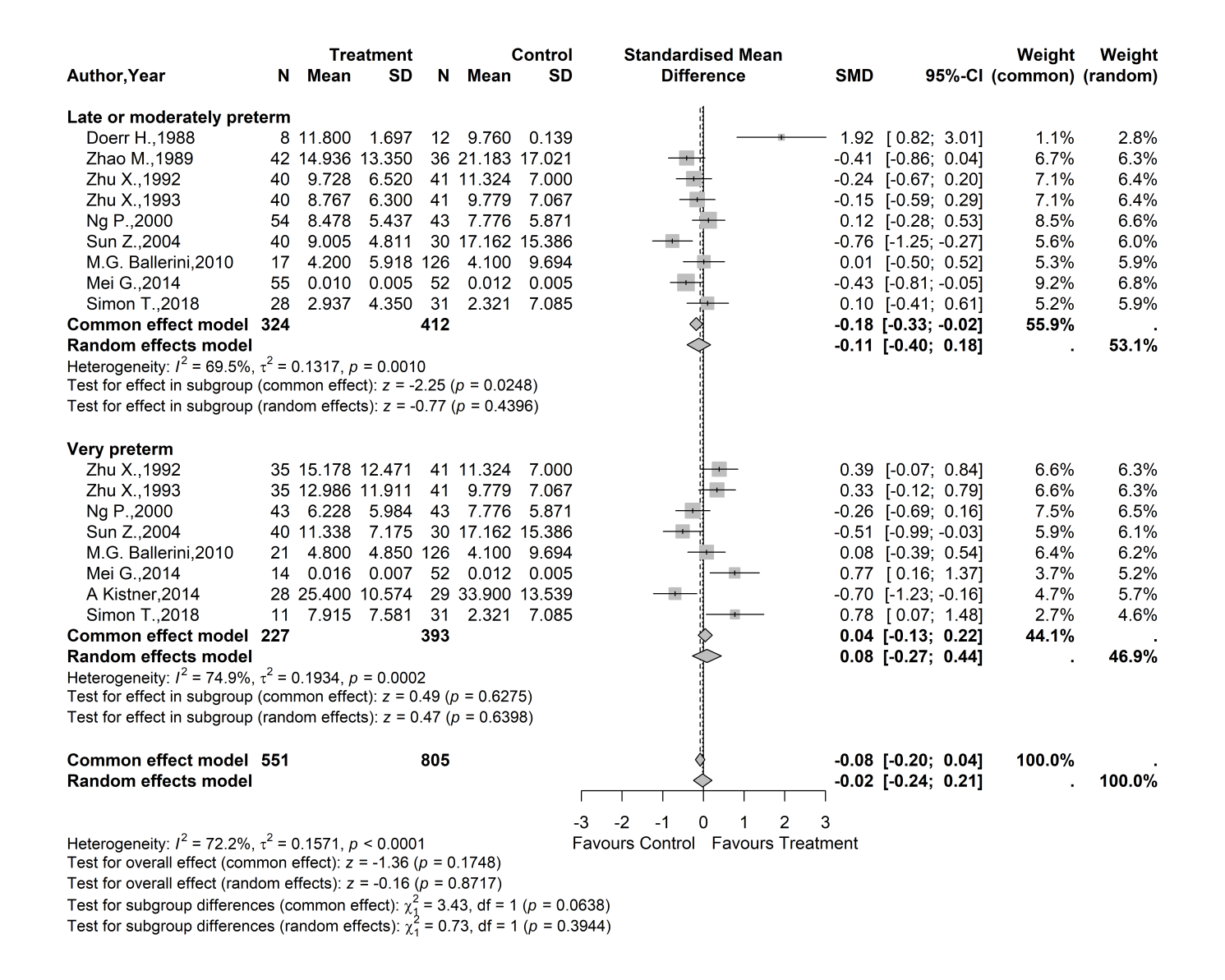


**Supplementary Figure 7.** Subgroup meta-analysis of peripheral blood cortisol levels by GA. A total of 551preterm infants and 805 term infants were included. Late or moderately preterm subgroup (n=100): The random-effects model yielded a pooled SMD of -0.11 (95% CI: -0.40. to 0.18, p =0.439), with heterogeneity I^2^=69.5%. Very preterm subgroup (n=227): The random-effects model yielded a pooled SMD of 0.08 (95% CI: -0.27 to 0.44, p =0.640), with heterogeneity I^2^ =74.9%.


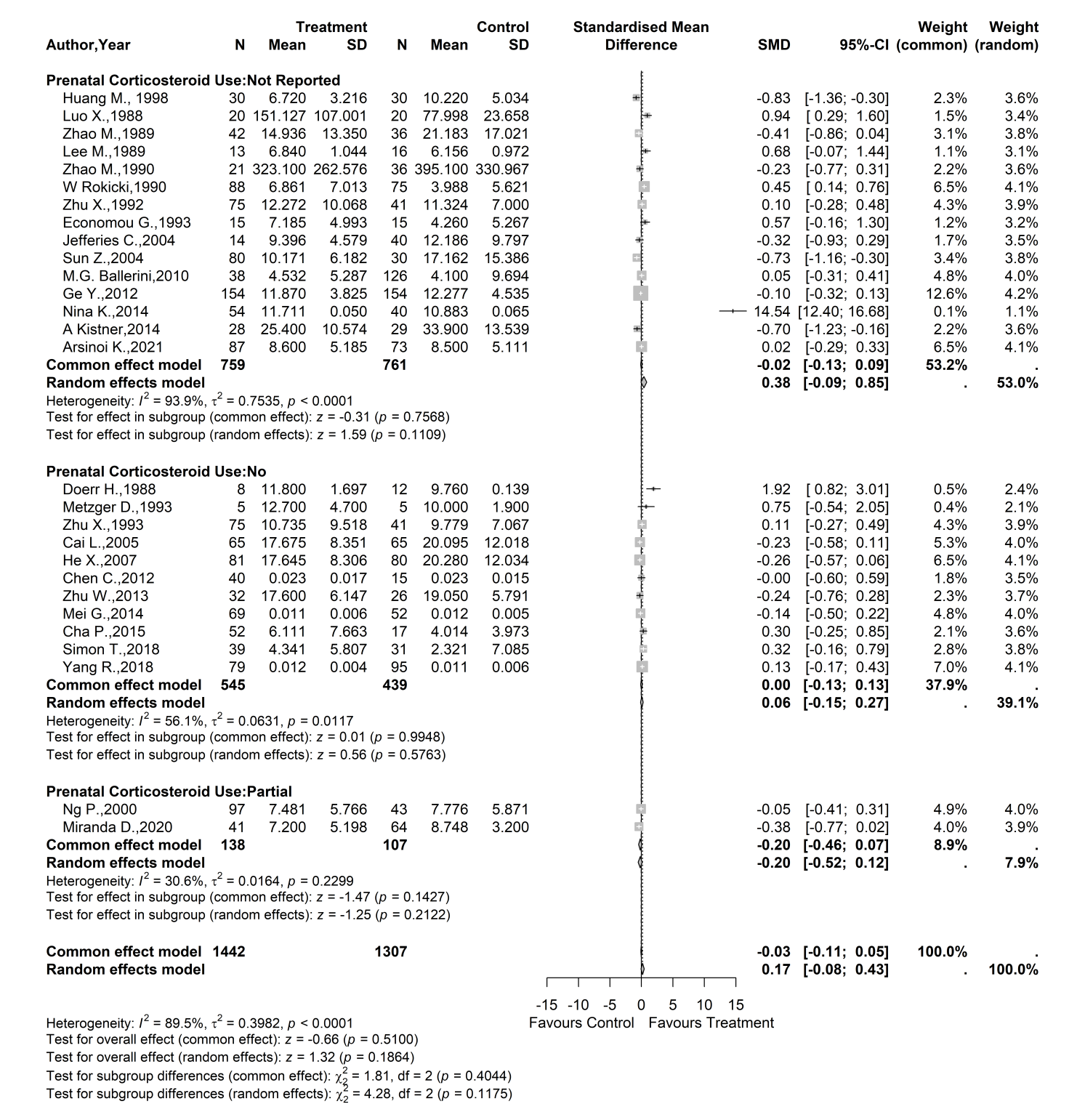


**Supplementary Figure 8.** Subgroup meta-analysis of peripheral blood cortisol levels by the use of corticosteroids prenatally. Prenatal corticosteroid did not report subgroup (n=759): The random-effects model yielded a pooled SMD of 0.83(95% CI: -0.09 to 0.85, p =0.576), with heterogeneity I^2^=56.1%, indicating that there was no difference between the two groups.Prenatal corticosteroid dont’t use subgroup (n=545): The random-effects model yielded a pooled SMD of 0.06 (95% CI: -0.15 to -0.27, p =0.576), with heterogeneity I^2^=56.1%. Prenatal corticosteroid use subgroup (n=138): The common-effects model yielded a pooled SMD of -0.20 (95% CI: -1.46 to 0.07, p = 0.143), with heterogeneity I^2^ =30.6%.


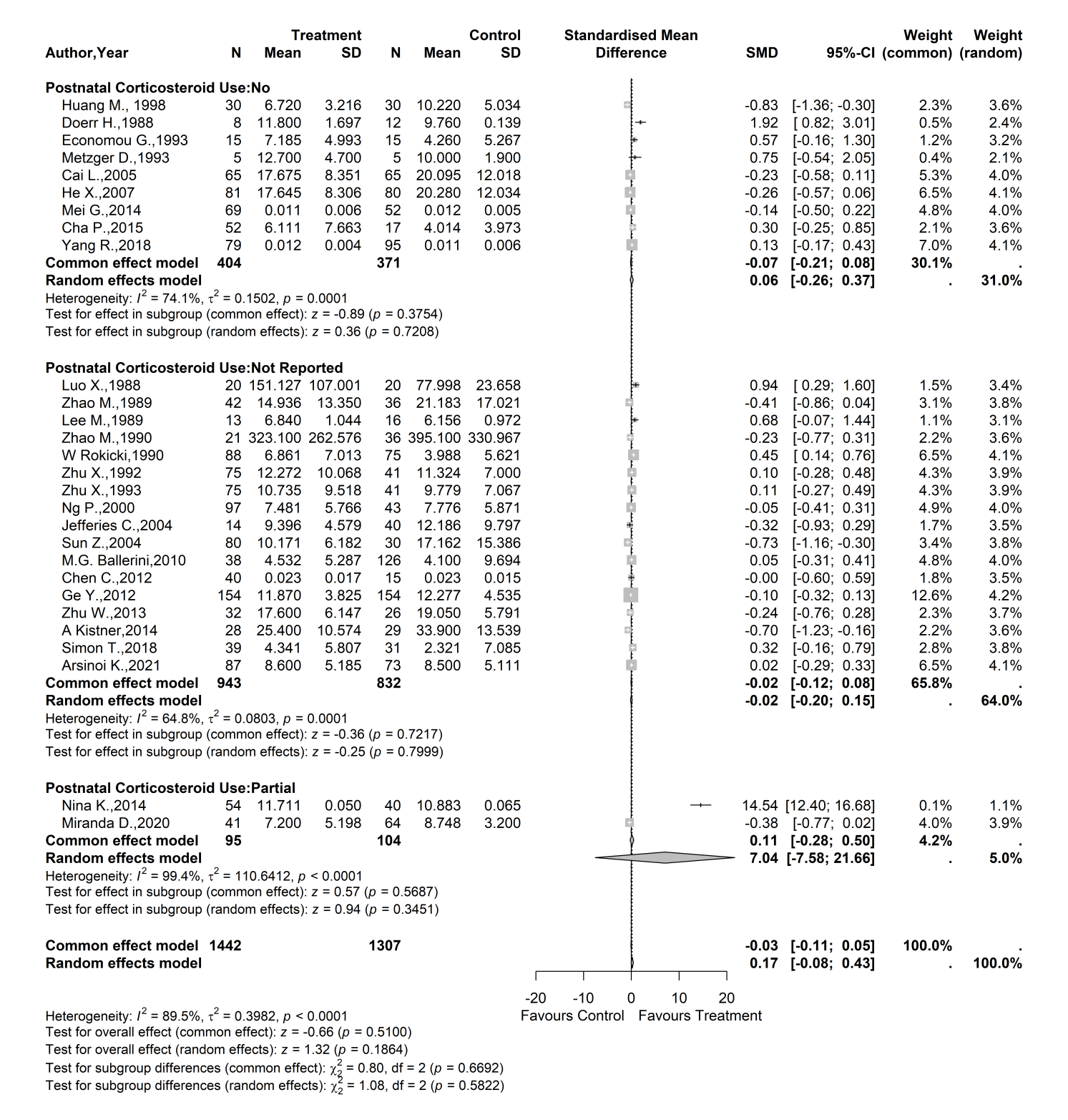


**Supplementary Figure 9.** Subgroup meta-analysis of peripheral blood cortisol levels by the use of corticosteroids postnatally. Postnatal corticosteroid dont’t use subgroup (n=404): The random-effects model yielded a pooled SMD of 0.06 (95% CI: -0.26 to 0.37, p =0.720), with heterogeneity I^2^=74.1%, Postnatal corticosteroid dont’t reported subgroup (n=943): The random-effects model yielded a pooled SMD of -0.02(95% CI: -0.20 to 0.15, p =0.800), with heterogeneity I^2^=64.8%, indicating that there was no difference in cortisol levels between two groups. Postnatal corticosteroid use subgroup (n=95): The random-effects model yielded a pooled SMD of 7.04(95% CI: -7.58 to 21.66, p =0.345), with heterogeneity I^2^=99.4%, indicating that there was no difference in cortisol levels between the two groups.


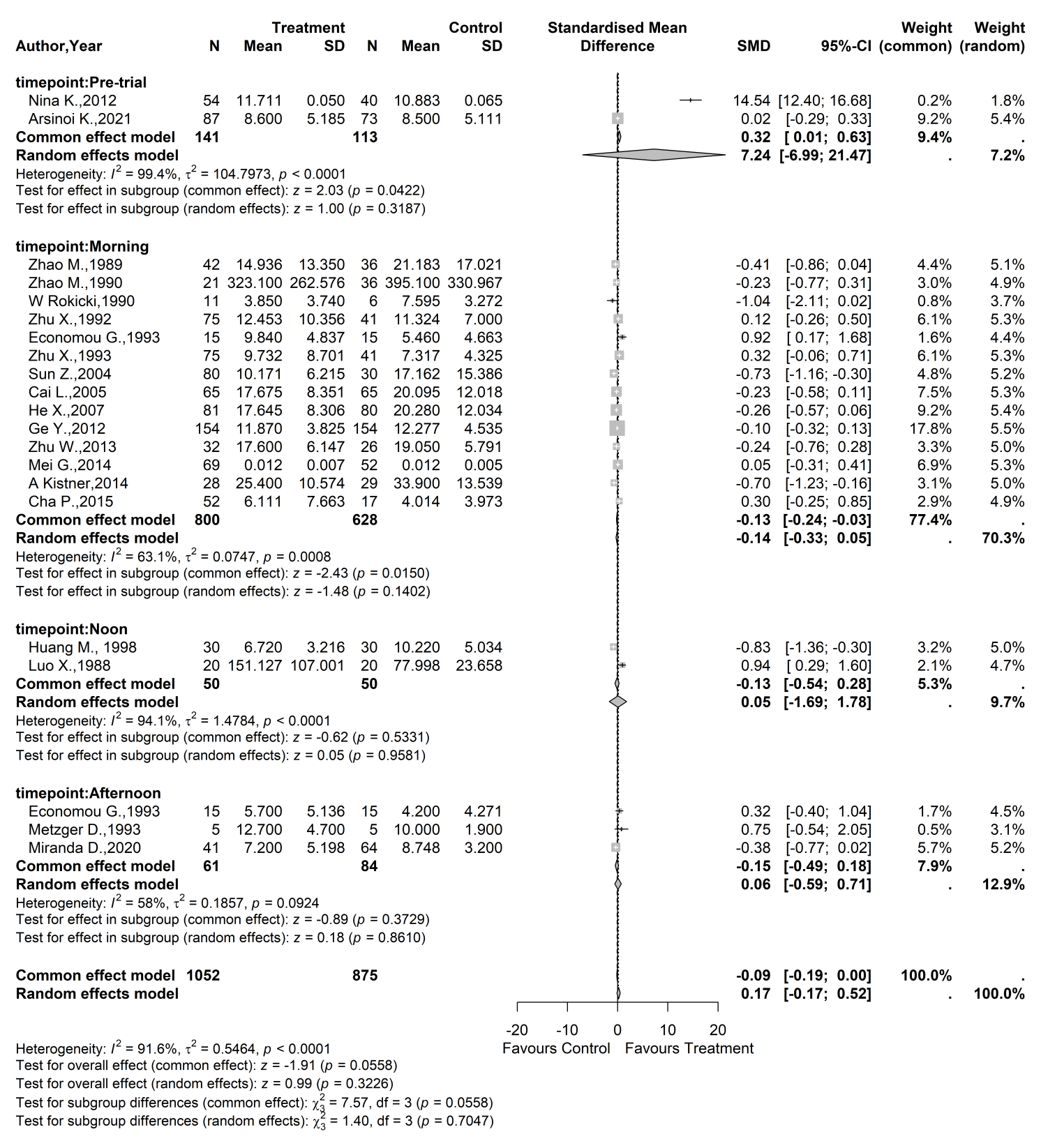


**Supplementary Figure 10.** Subgroup meta-analysis of peripheral blood cortisol levels by measurement time. They were divided into four groups according to the detection time: pre-experiment group (pre-intervention), morning group (before 10:00 a.m.), noon group, and afternoon group (after 2:00 p.m.). No significant difference was observed in cortisol levels between preterm infants and term infants at different measurement time points.


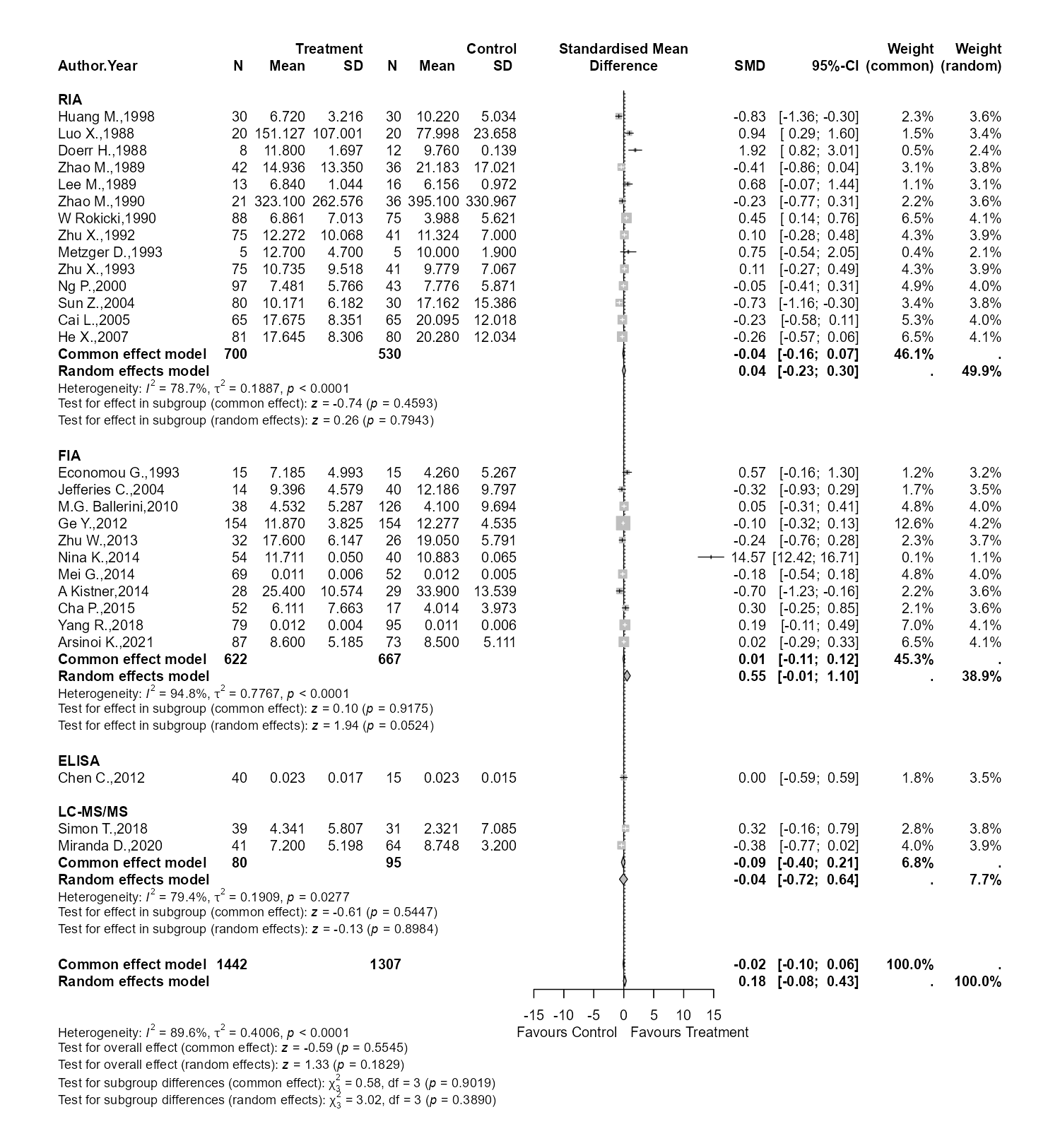


**Supplementary Figure 11.** Subgroup meta-analysis of peripheral blood cortisol levels by measurement methods. RIA subgroup: The random-effects model yielded a pooled SMD of 0.04 (95% CI: -0.23 to 0.30, p =0.794), with heterogeneity I^2^=78.7%. FIA subgroup: The random-effects model yielded a pooled SMD of 0.55 (95% CI: -0.01 to 1.10, p =0.052), with heterogeneity I^2^=94.8%. LC-MS/MS subgroup: The random-effects model yielded a pooled SMD of -0.04 (95% CI: -0.72 to 0.64, p = 0.898), with heterogeneity I^2^ =79.4%.


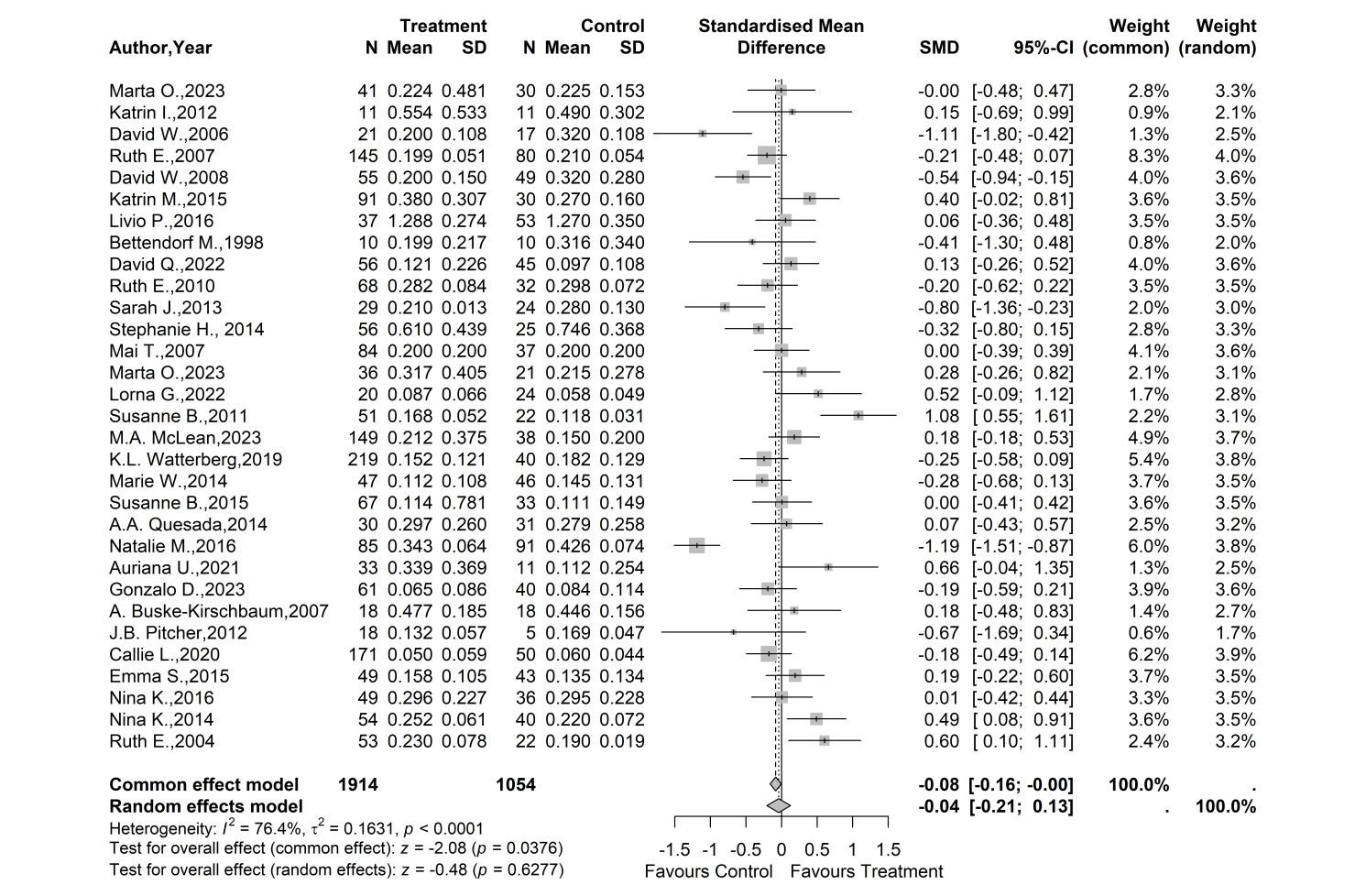


**Supplementary Figure 12.** Forest plot for salivary cortisol levels. Each study’s standardized mean difference (SMD) and 95% confidence interval (CI) are shown, with square size indicating study weight. Heterogeneity was high (I²= 79.4%, p < 0.001). Pooled analysis found no significant difference in cortisol levels between groups (random effects model: SMD = -0.04, 95% CI [-0.21, 0.13], p = 0.6277).


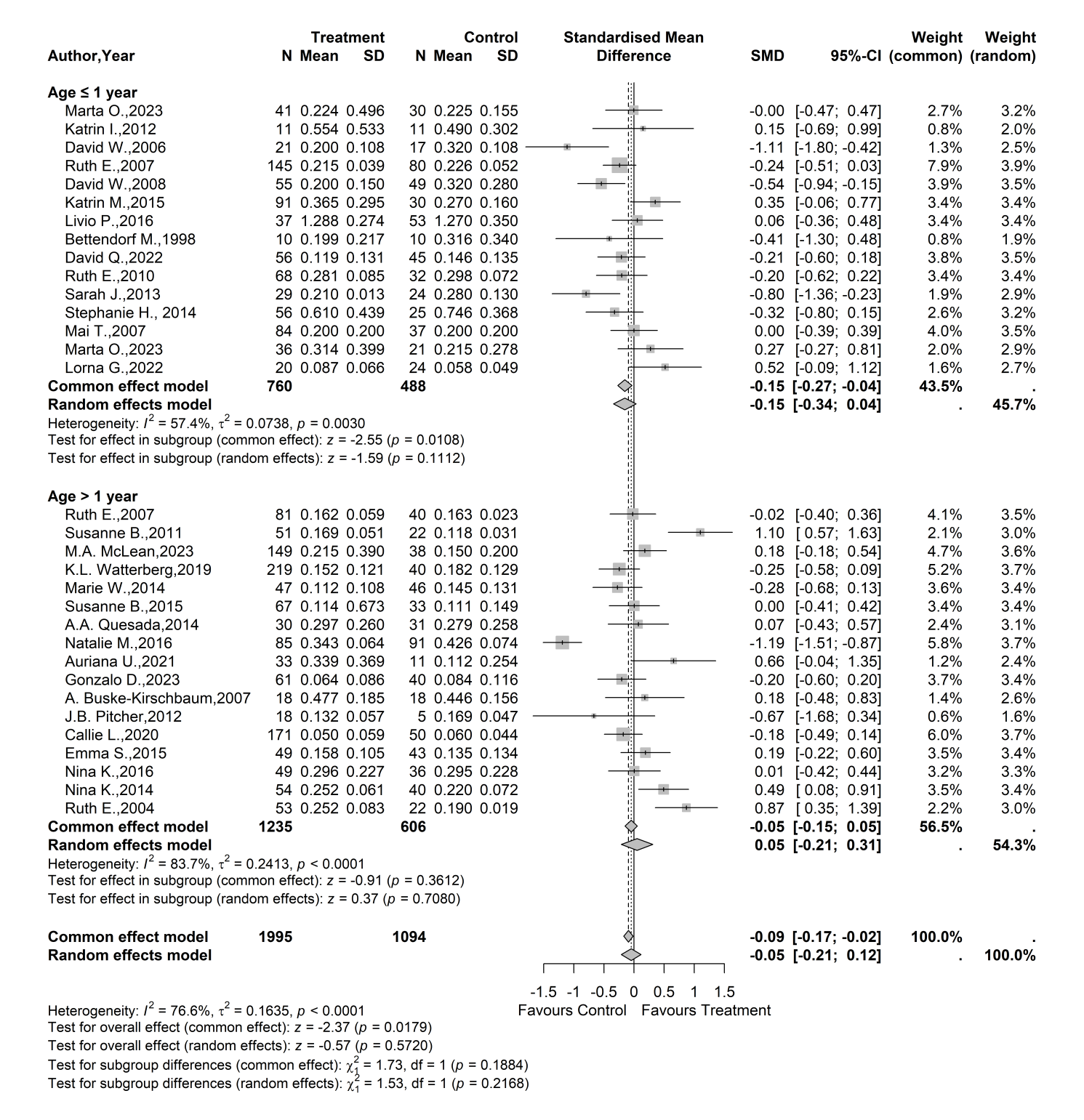


**Supplementary Figure 13.** Forest plot of subgroup meta-analysis of salivary cortisol levels between preterm and term infants, stratified by infant age (≤1 year vs. >1 year). High heterogeneity was observed in both subgroups (≤1 year: I²= 57.4%, p = 0.0030; >1 year: I² = 83.7%, p < 0.0001). Pooled analyses found no significant differences in cortisol levels between preterm and term infants across subgroups or overall (overall random effects: SMD = -0.05, 95% CI [-0.21, 0.12]).
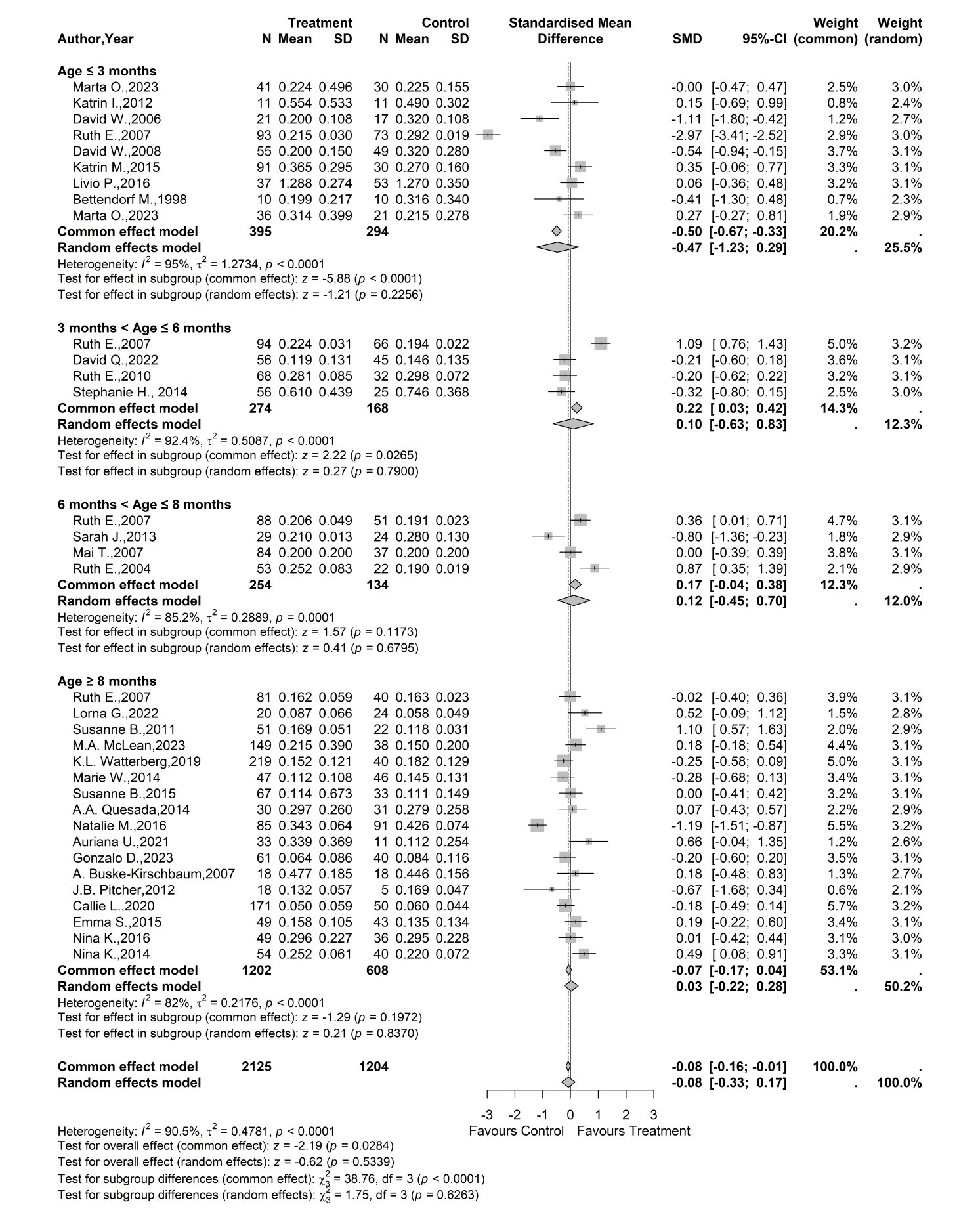


**Supplementary Figure 14.** Subgroup meta-analysis of salivary cortisol levels during different time periods: ≤3 months, 3~6 months, 6~8 months, ≥8 months. High heterogeneity was observed in subgroups (≤3 months: I²= 95.0%; 3~6 months: I²= 92.4%; 6~8 months: I²= 85.2%; ≥8 months:I ²= 83.7%,). Pooled analyses found no significant differences in cortisol levels across subgroups or overall (overall random effects: SMD = -0.08, 95% CI [-0.33, 0.17])


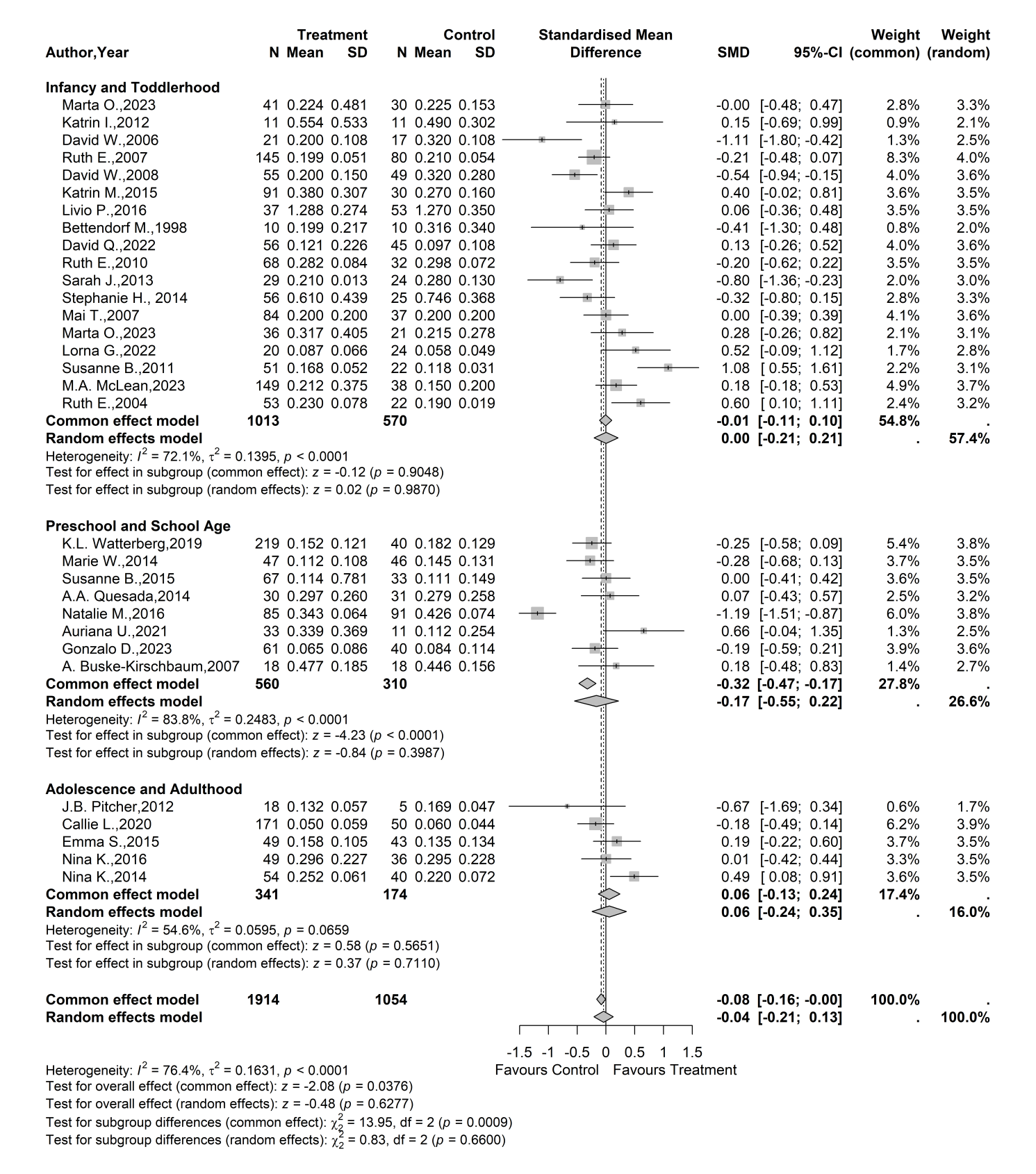


**Supplementary Figure 15.** Subgroup meta-analysis of salivary cortisol levels during different time periods: infancy and toddlerhood, preschool and school age, adolescence and adulthood. Pooled analyses found no significant differences in cortisol levels across subgroups or overall.


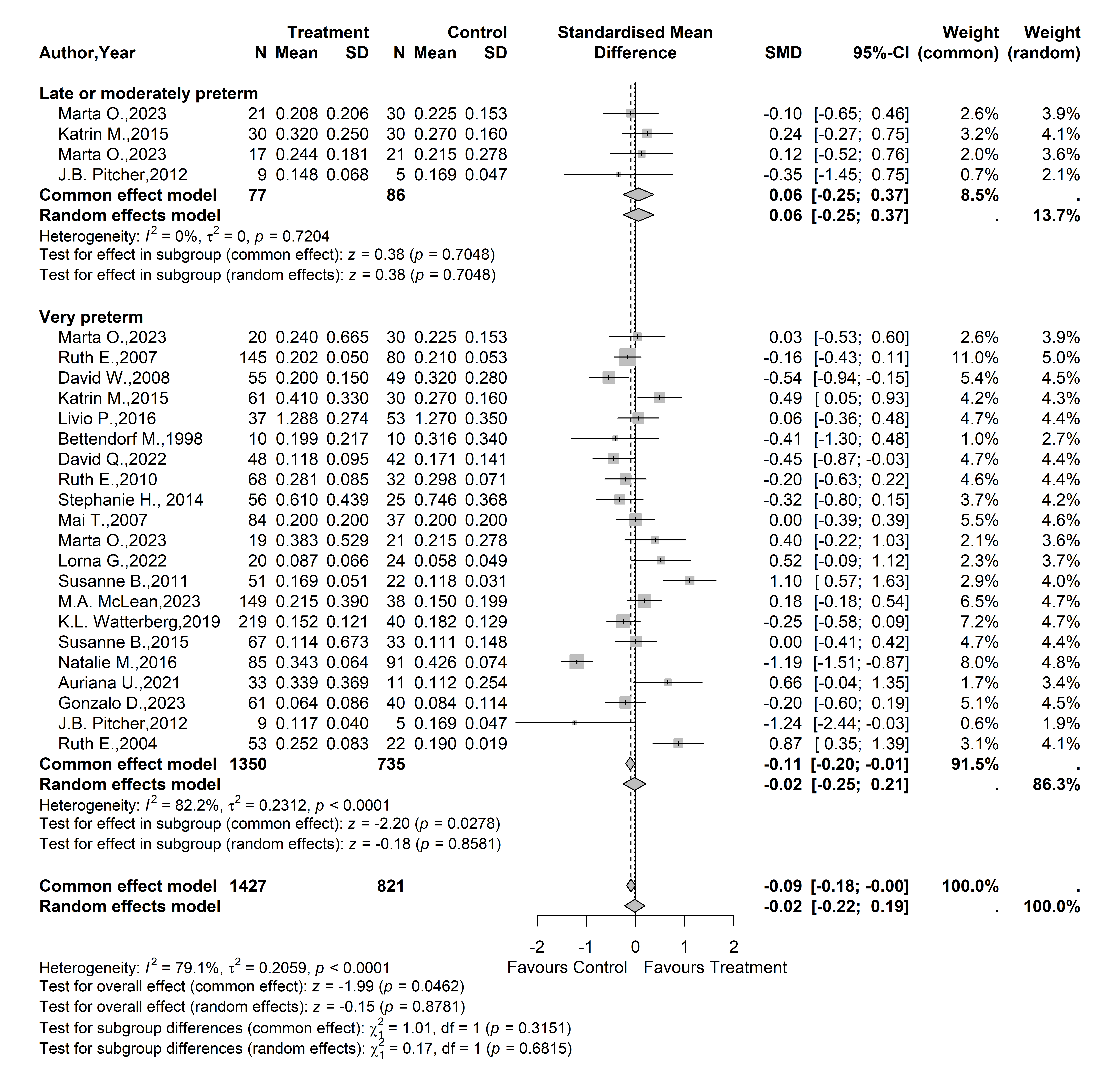


**Supplementary Figure 16.** Subgroup meta-analysis of salivary cortisol levels by GA. Pooled analyses found no significant differences in cortisol levels across subgroups or overall.


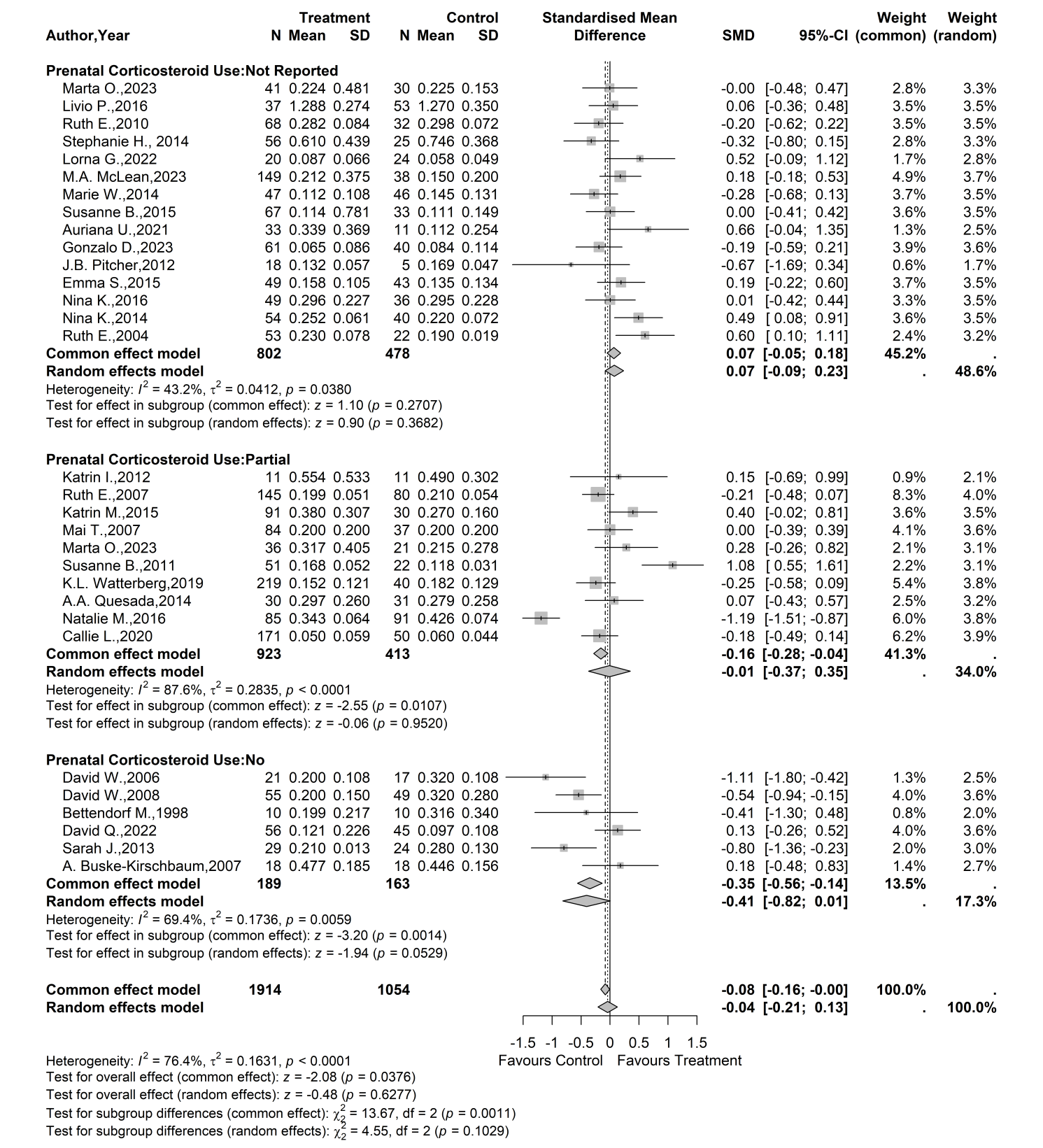


**Supplementary Figure 17.** Subgroup meta-analysis of salivary cortisol by the use of corticosteroids prenatally. Pooled analyses found no significant differences in cortisol levels across subgroups or overall.


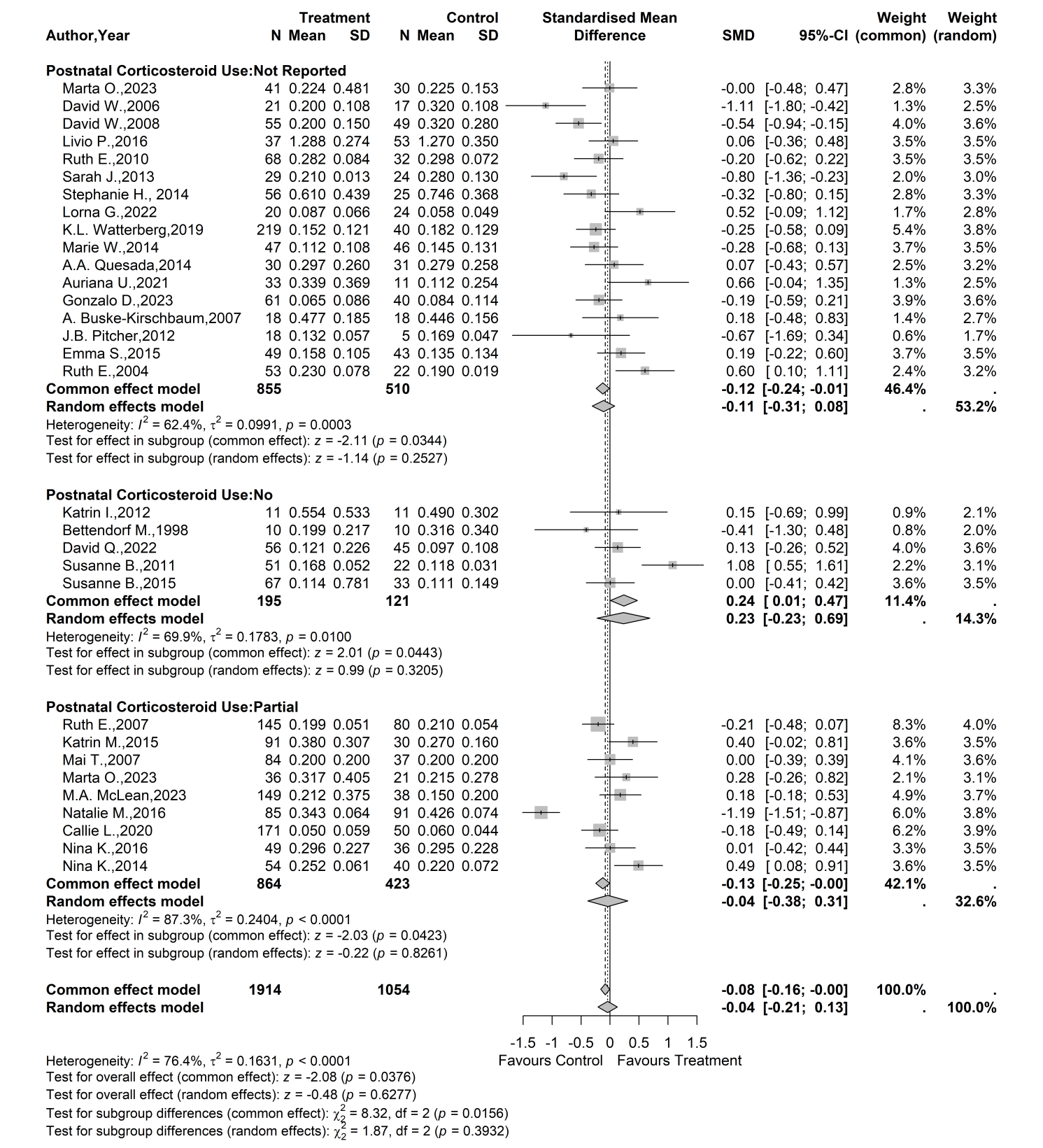


**Supplementary Figure 18.** Subgroup meta-analysis of salivary cortisol by the use of corticosteroids postnatally. Pooled analyses found no significant differences in cortisol levels across subgroups or overall.


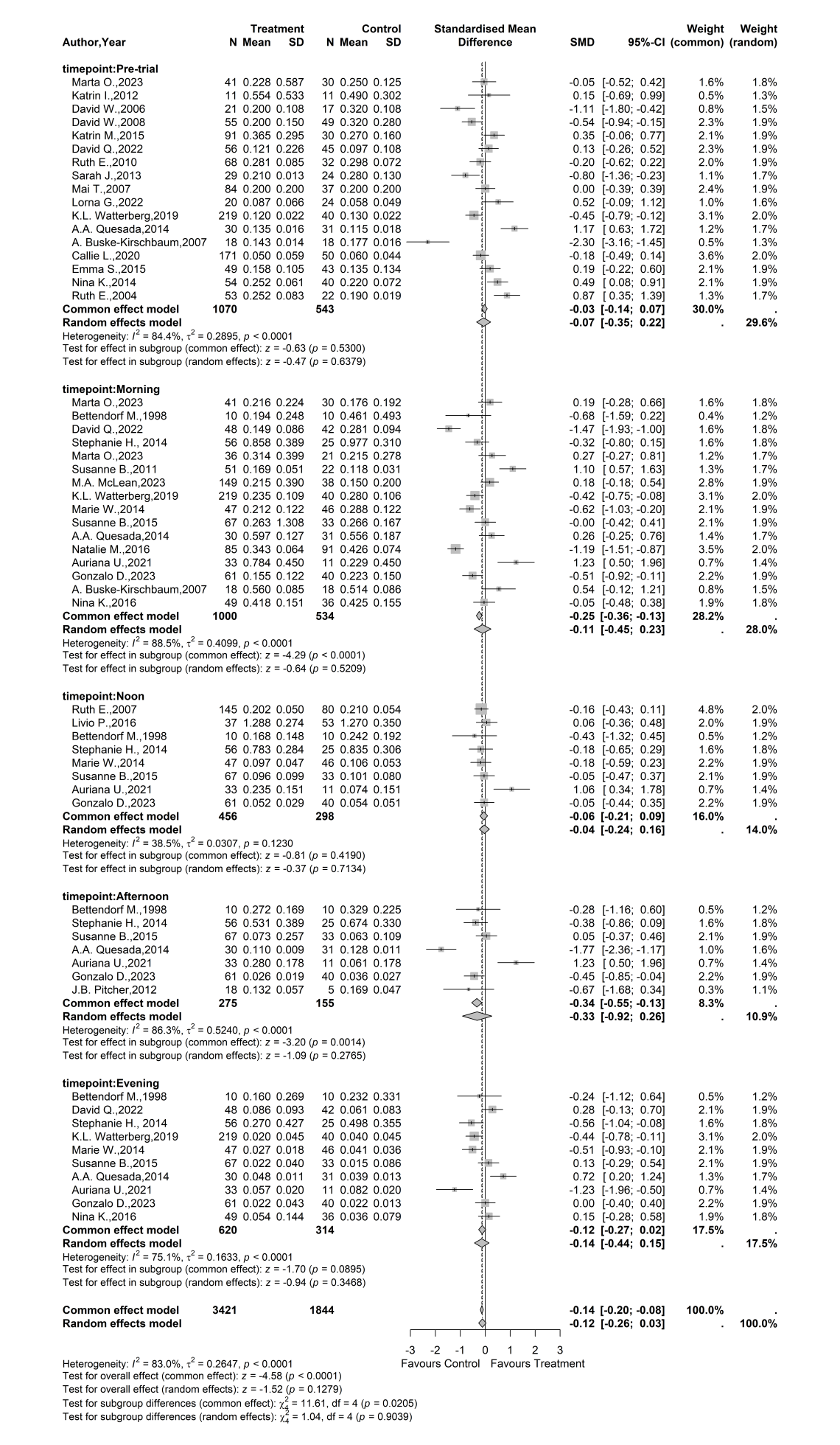


**Supplementary Figure 19.** Subgroup meta-analysis of salivary cortisol levels by measurement time. Pooled analyses found no significant differences in cortisol levels across subgroups or overall.


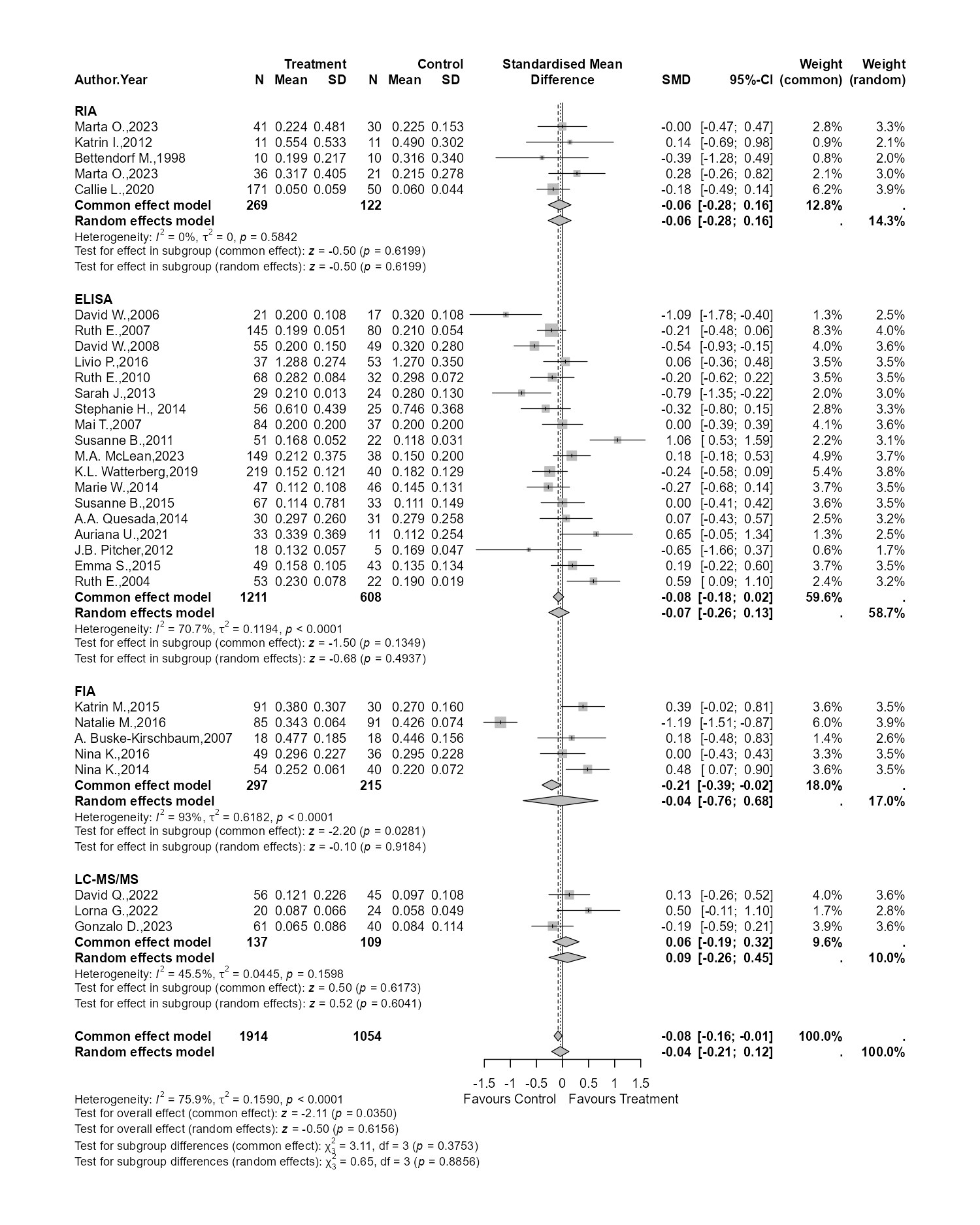


**Supplementary Figure 20.** Subgroup meta-analysis of salivary cortisol levels by measurement methods. RIA subgroup: The common-effects model yielded a pooled SMD of -0.06 (95% CI: -0.28 to 0.16, p =0.794), with heterogeneity I^2^=0%. FIA subgroup: The random-effects model yielded a pooled SMD of -0.04 (95% CI: -0.76 to 0.68, p =0.9184), with heterogeneity I^2^=93%. LC-MS/MS subgroup: The random-effects model yielded a pooled SMD of 0.09 (95% CI: -0.26 to 0.45, p = 0.604), with heterogeneity I^2^ =45.5%.


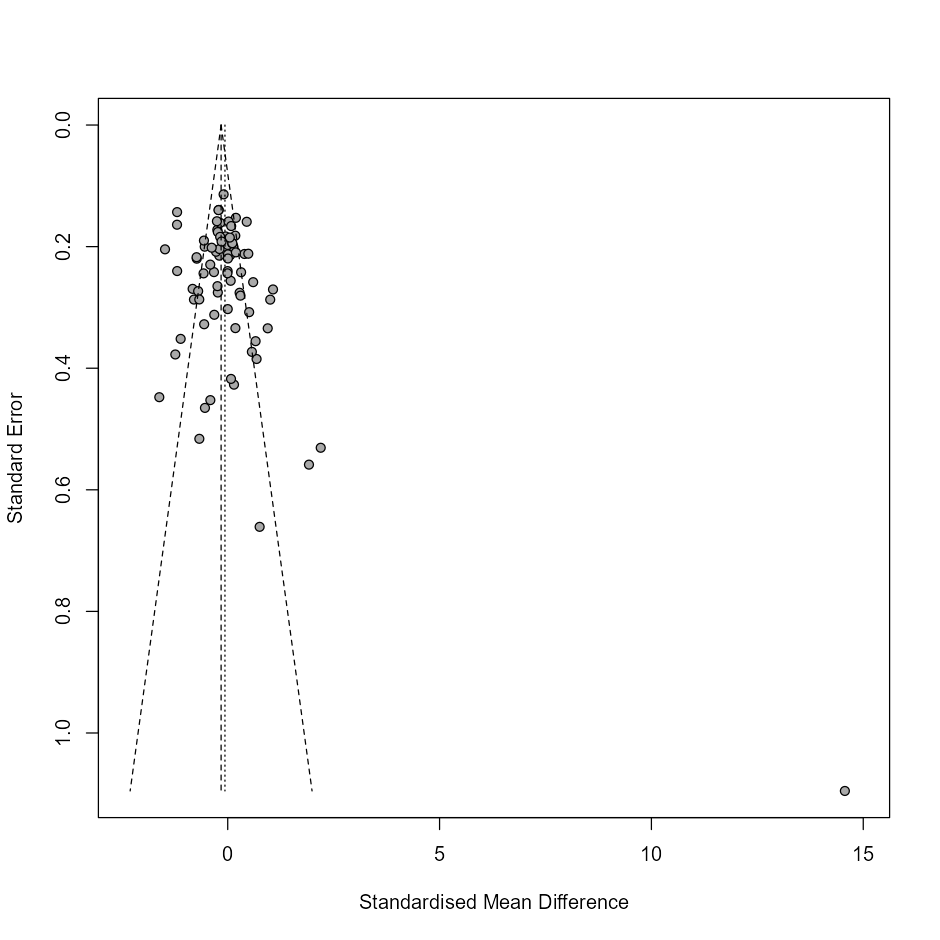


**Supplementary Figure 21.** Funnel plot for 74 studies' publication bias assessment.This funnel plot displays the standard error of the standardized mean difference (SMD) against the SMD for each included study. The vertical dotted line represents the pooled overall effect size, while the diagonal dashed lines indicate the 95% confidence interval boundaries for funnel plot asymmetry.

**
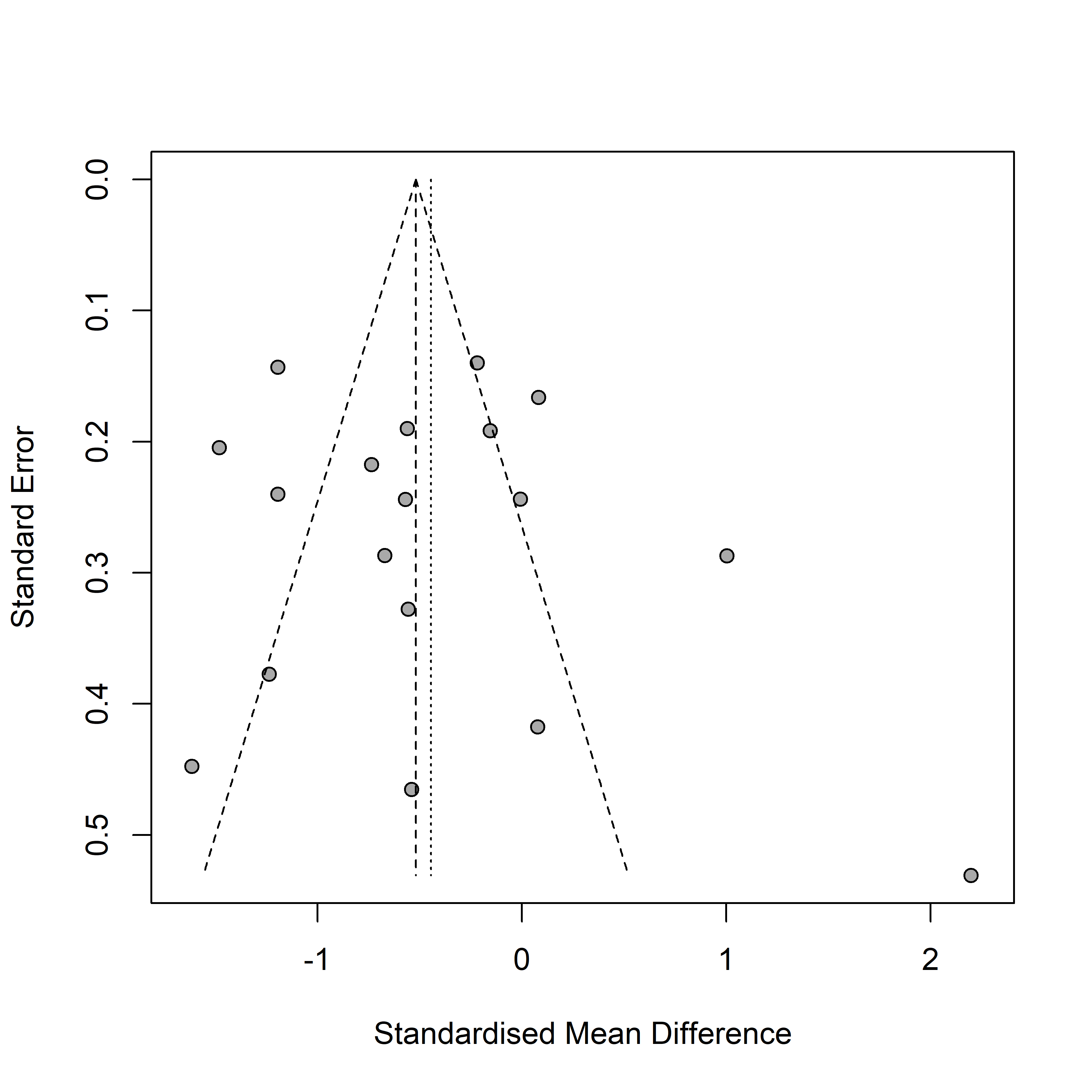
**

**Supplementary Figure 22.** Funnel plot for UCB studies' publication bias assessment.


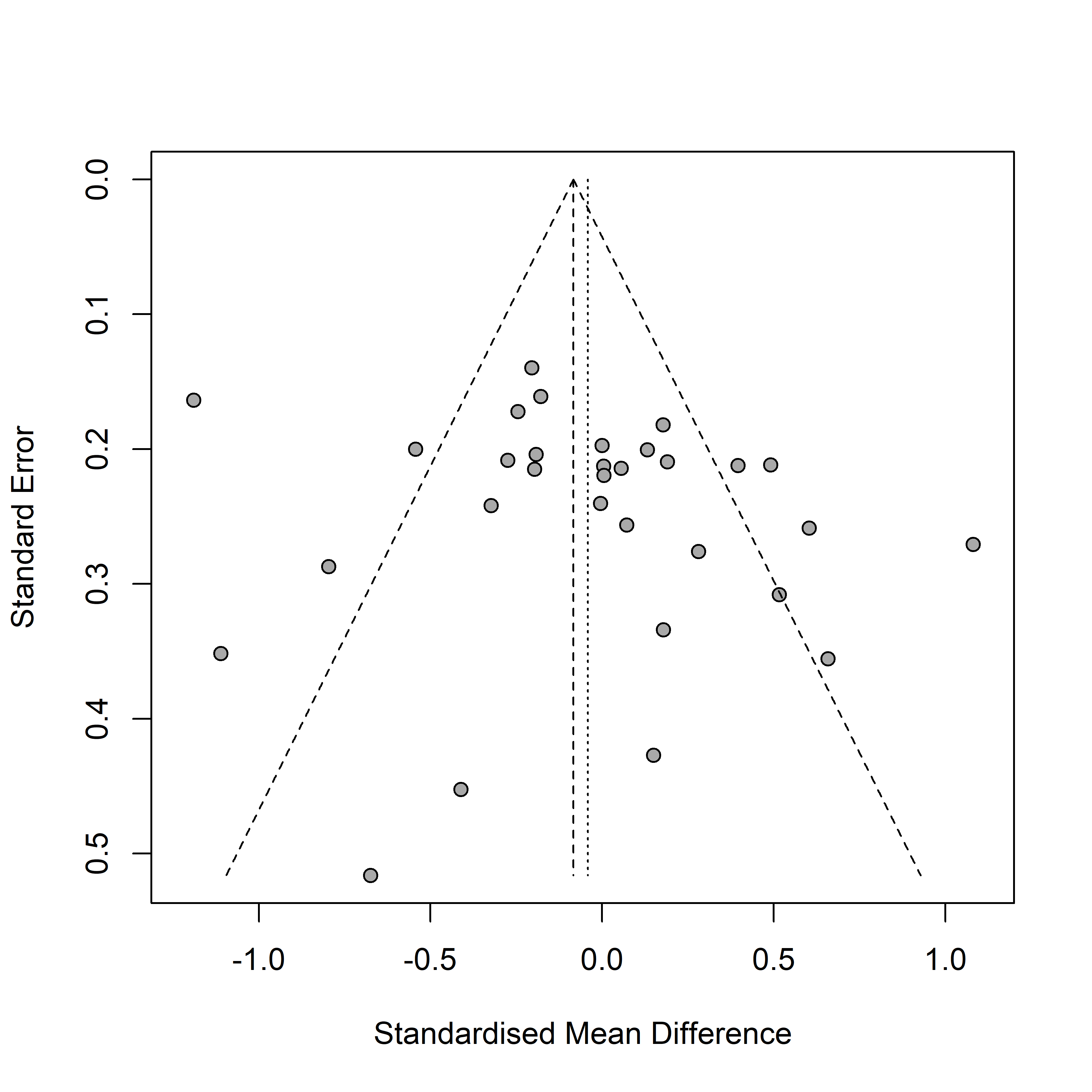


**Supplementary Figure 23.** Funnel plot for salivary studies' publication bias assessment..


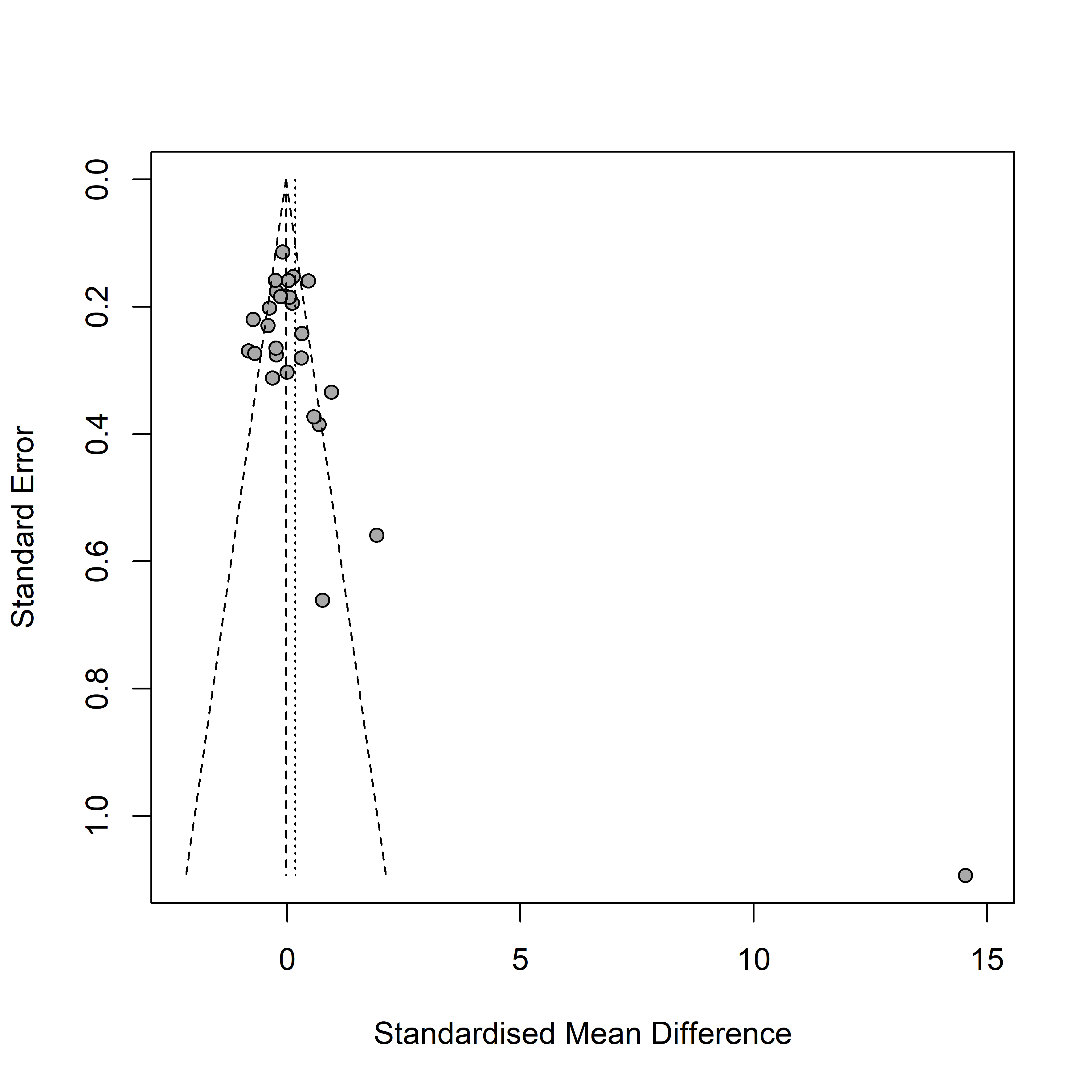


**Supplementary Figure 24.** Funnel plot for the peripheral blood studies' publication bias assessment.


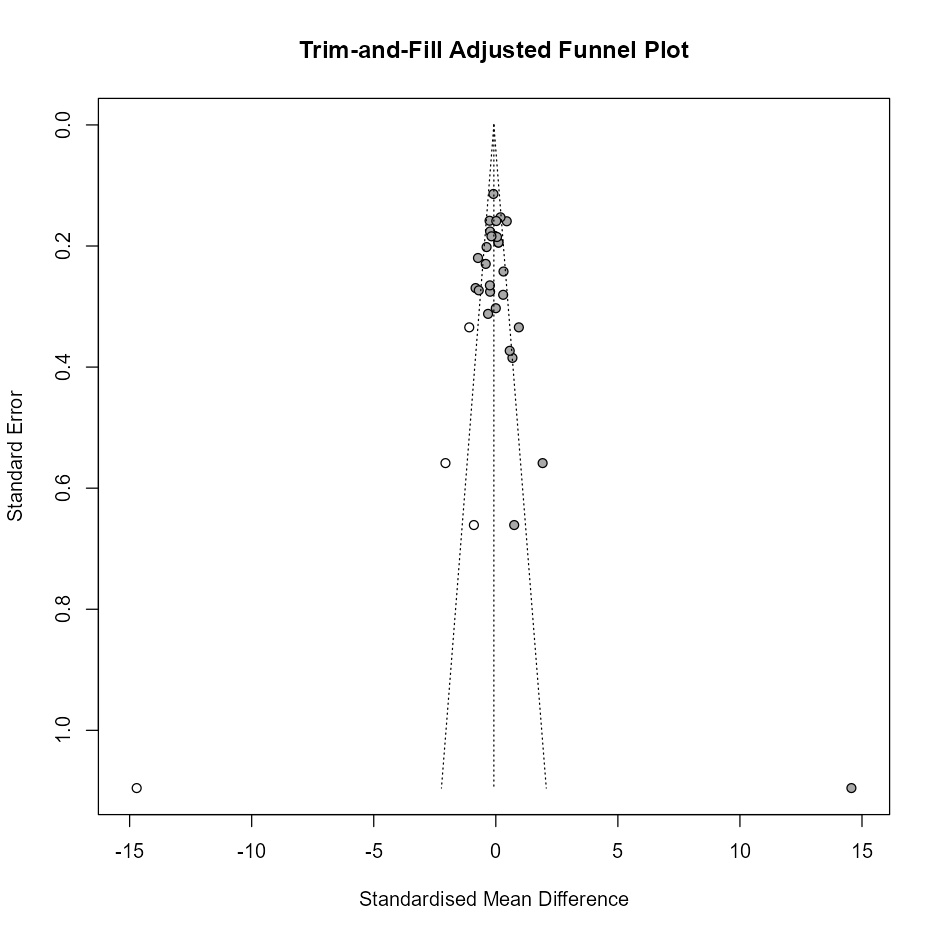


**Supplementary Figure 25.** Trim-and-fill adjusted funnel plot for the peripheral blood studies' publication bias assessment.


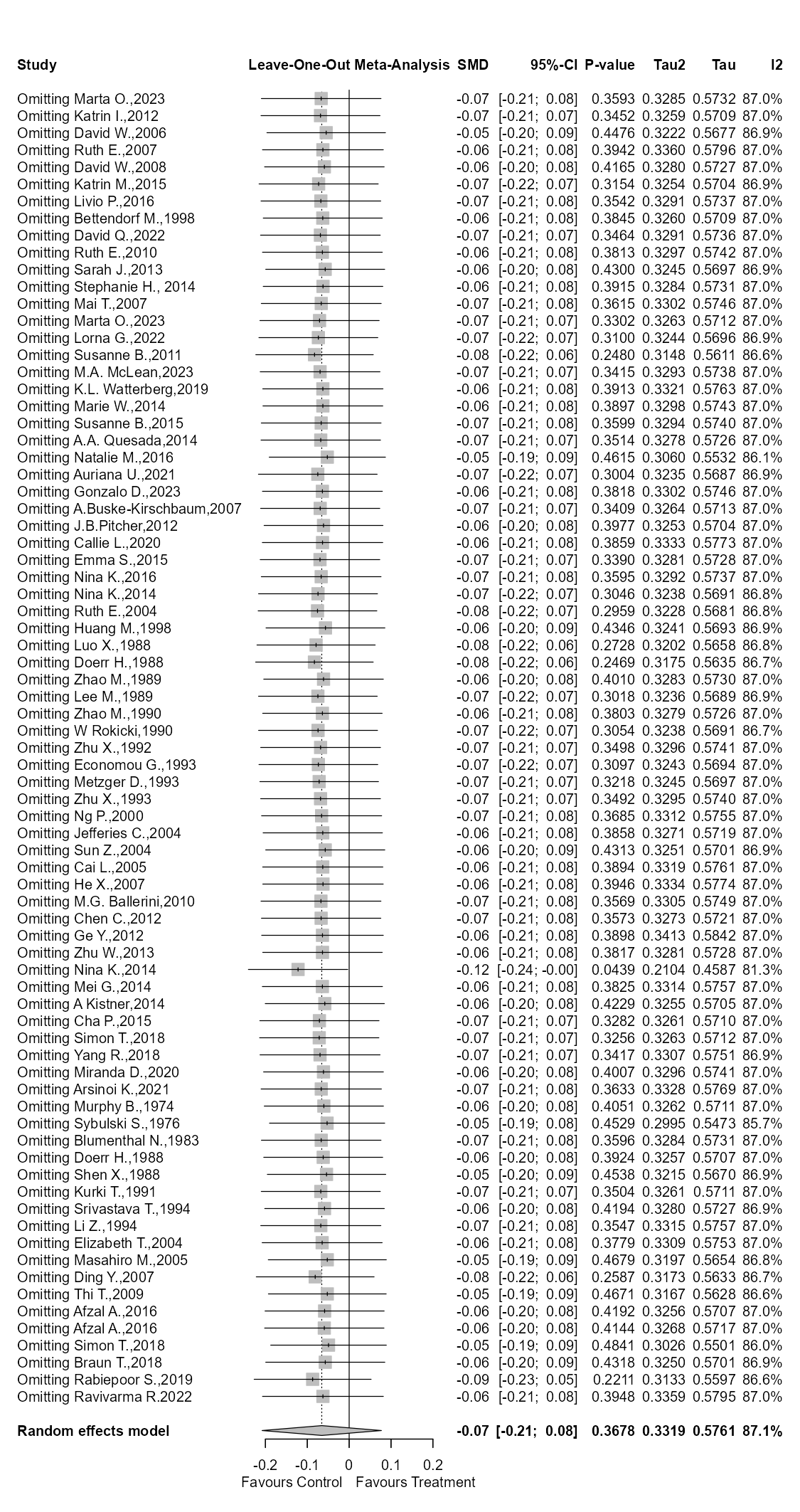


**Supplementary Figure 26.** Leave-one-out sensitivity analysis of the standardized mean difference (SMD) in cortisol levels between preterm and term infants.


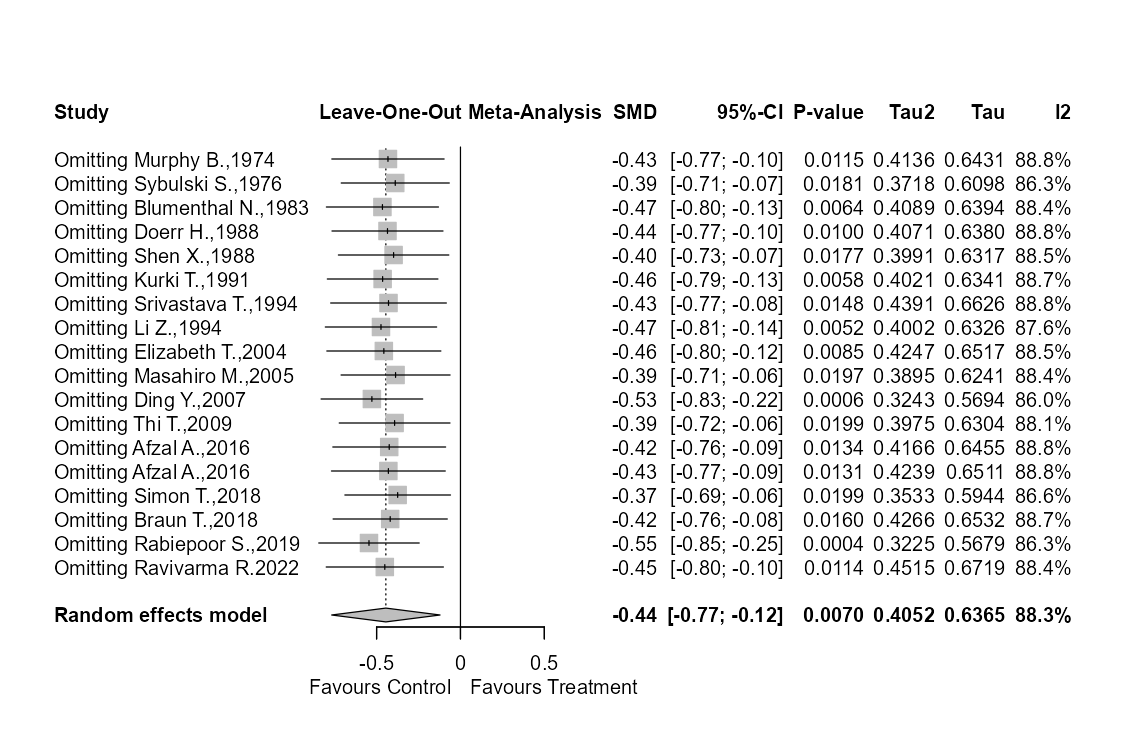


**Supplementary Figure 27.** Leave-one-out sensitivity analysis of the standardized mean difference (SMD) in umbilical cord blood cortisol levels between preterm and term infants.


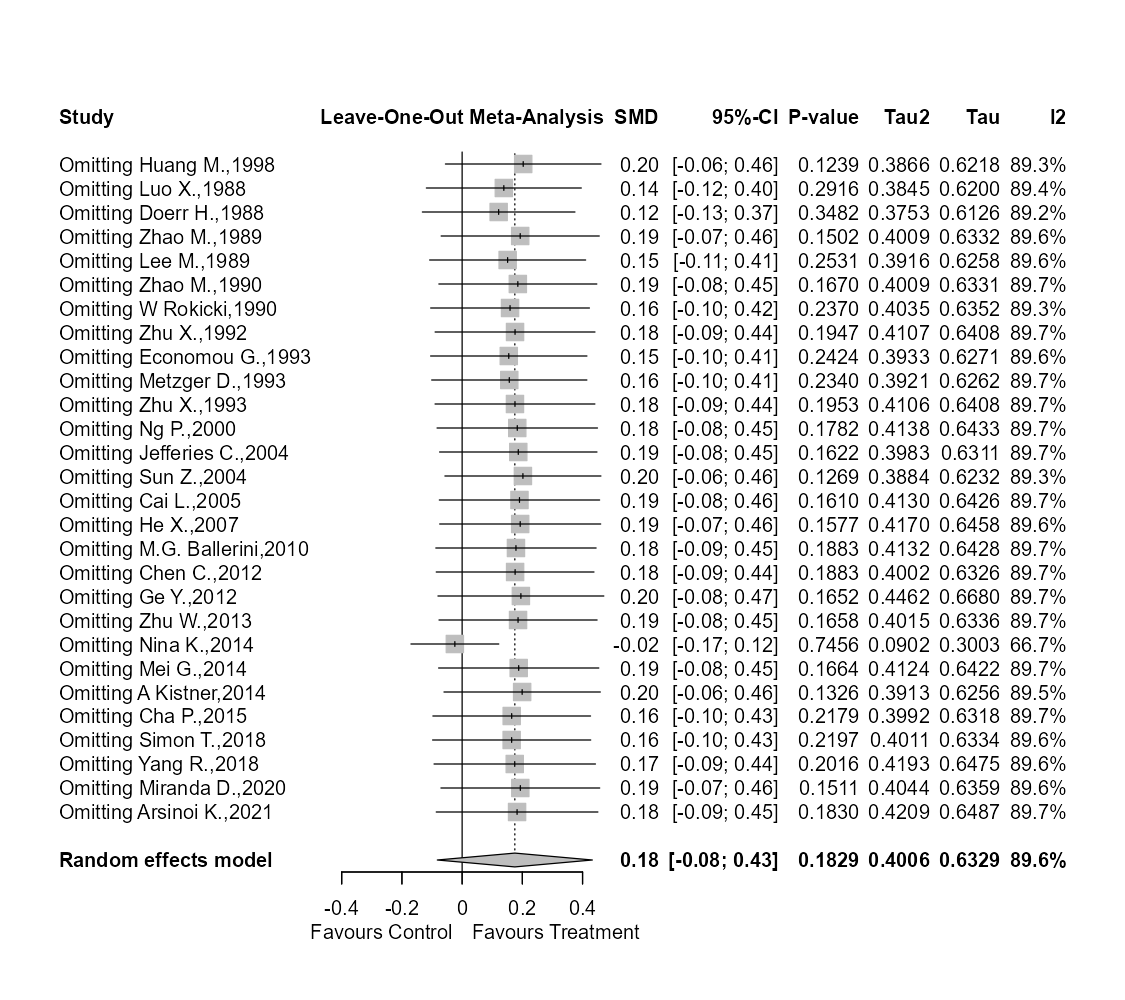


**Supplementary Figure 28.** Leave-one-out sensitivity analysis of the standardized mean difference (SMD) in peripheral blood cortisol levels between preterm and term infants.


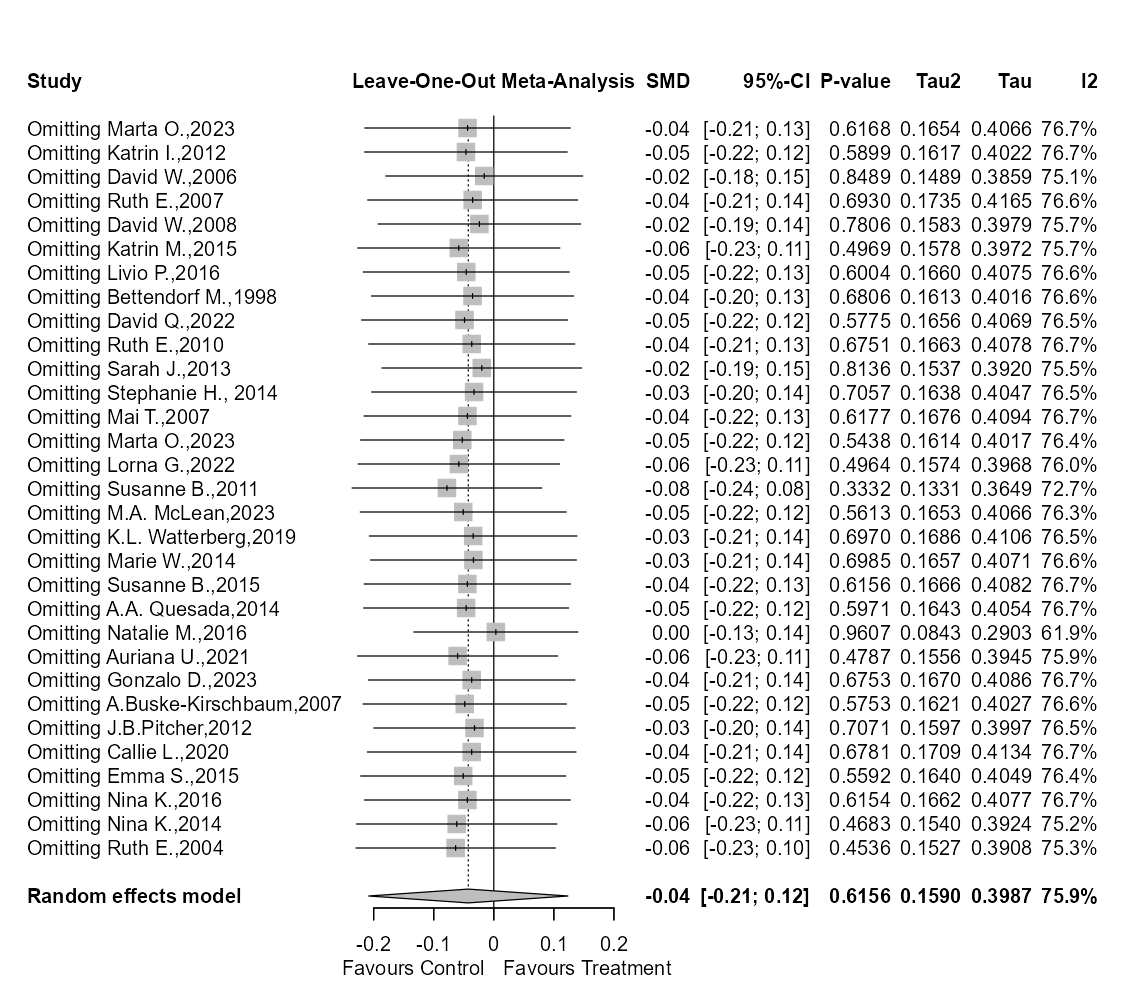


**Supplementary Figure 29.** Leave-one-out sensitivity analysis of the standardized mean difference (SMD) in salivary cortisol levels between preterm and term infants.


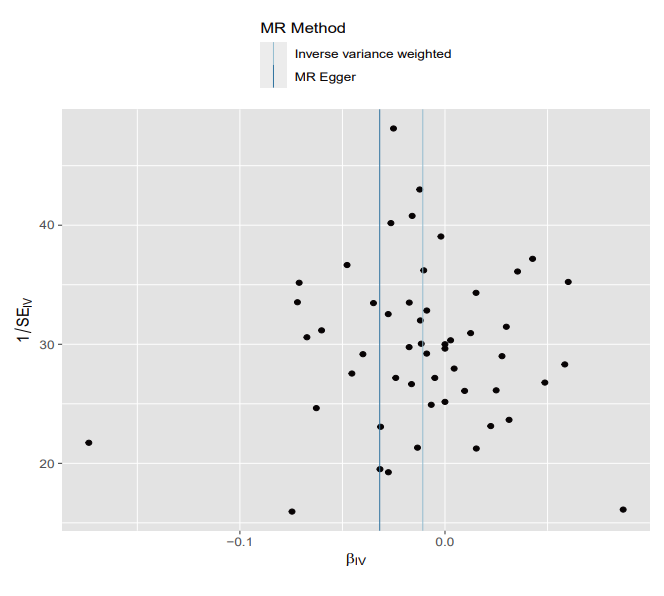


**Supplementary Figure 30.** The presence of heterogeneity in Mendelian randomization analyses. The plot shows 1/SE_IV_ againstβ_IV_for each genetic variant. The solid vertical line denotes the inverse variance weighted (IVW) pooled effect, and the dashed line shows the MR Egger result.


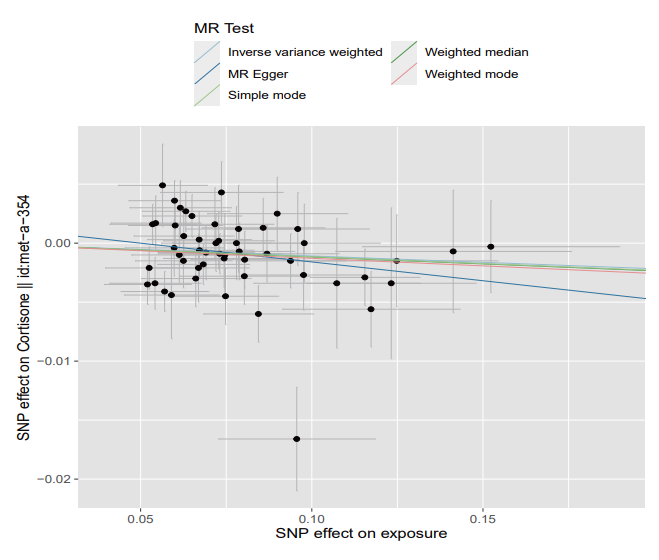


**Supplementary Figure 31.** The presence of pleiotropic effects in Mendelian randomization analyses.


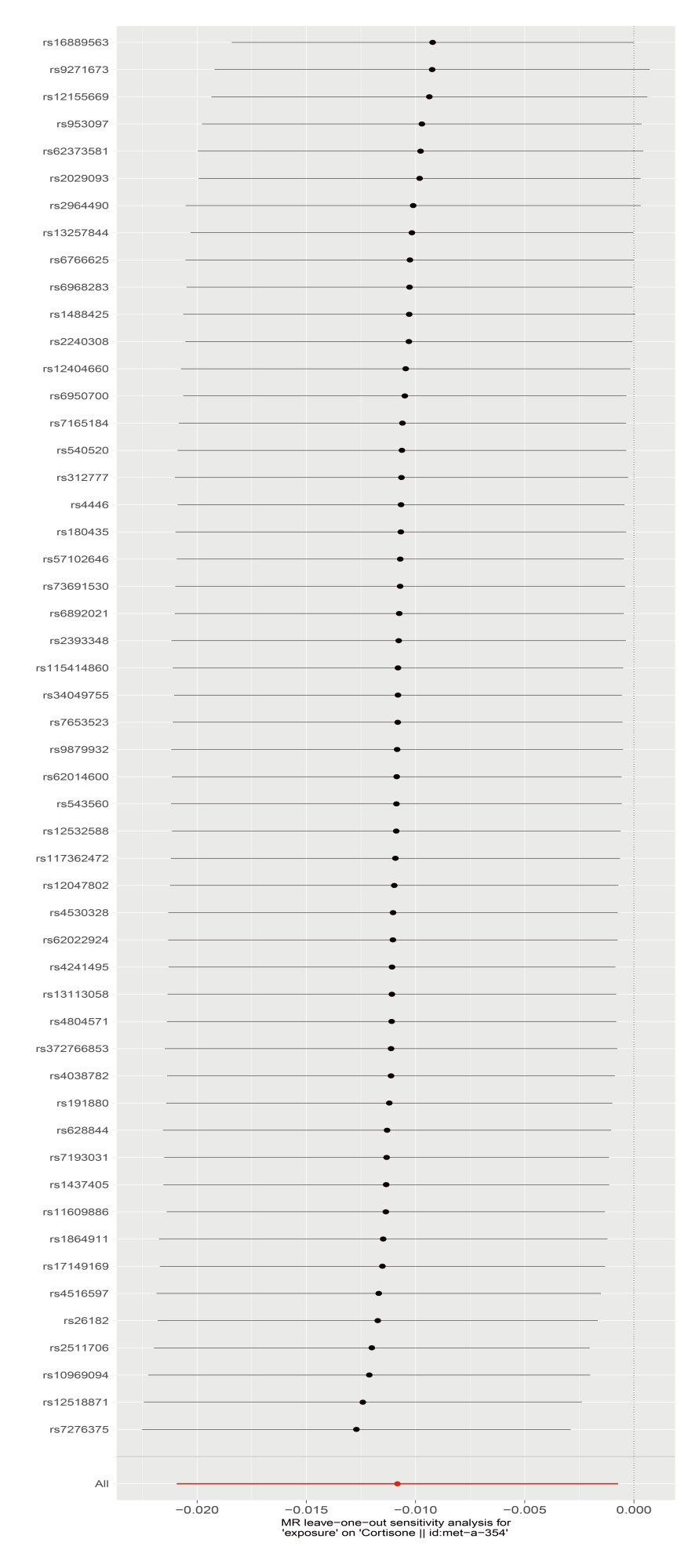


**Supplementary Figure 32.** The results of the leave-one-out in Mendelian randomization analyses.


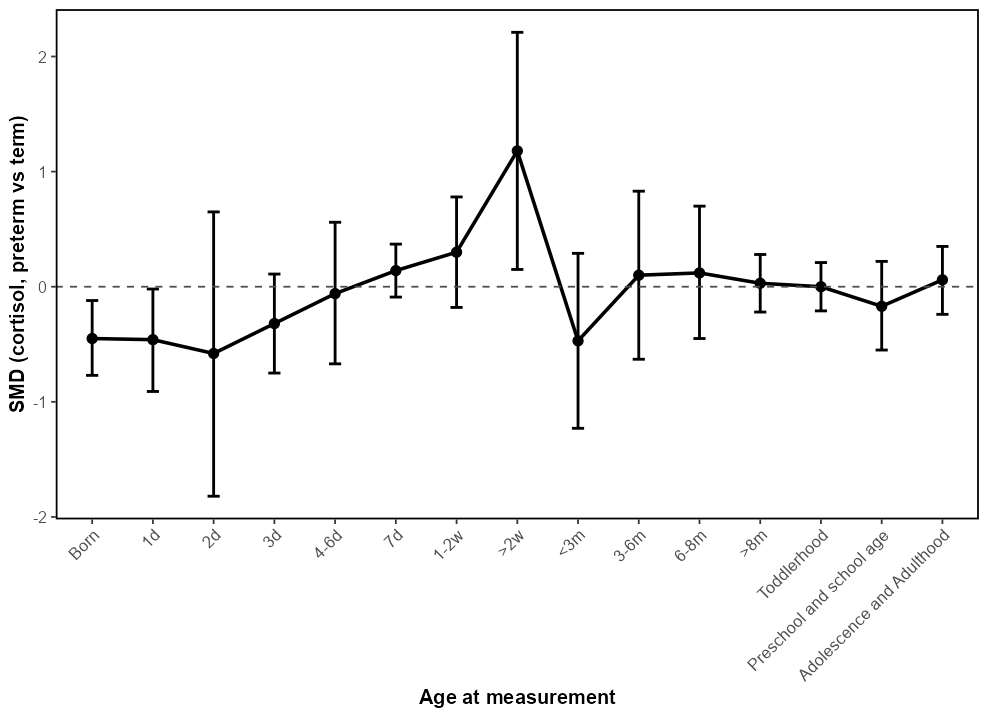


**Supplementary Figure 33.** Changes in the difference of cortisol levels between preterm and term infants measured by standardized mean difference (SMD). SMD < 0 indicates lower cortisol levels in preterm infants compared with term infants, while SMD > 0 indicates higher cortisol levels in preterm infants. The error bars represent the 95% confidence intervals (CI) for the SMD at each time point. The dashed line at y=0 serves as the reference line for no difference. A statistically significant difference is indicated when the 95% CI does not intersect wiSupplementary Tables

**2 Supplementary Tables**

| Database | Search strategy | |
| --- | --- | --- |
| Pubmed | #1 | ((((((((((Hydrocortisone[MeSH Terms]) OR (Pregn-4-ene-3,20-dione, 11,17,21-trihydroxy-, (11beta)-[Title/Abstract])) OR (Cortisol[Title/Abstract])) OR (Cortifair[Title/Abstract])) OR (Cortril[Title/Abstract])) OR (Hydrocortisone, (9 beta,10 alpha,11 alpha)-Isomer[Title/Abstract])) OR (Hydrocortisone, (11 alpha)-Isomer[Title/Abstract])) OR (Epicortisol[Title/Abstract])) OR (11-Epicortisol[Title/Abstract])) OR (11 Epicortisol[Title/Abstract])) OR (Cortef[Title/Abstract]) |
|  | #2 | (((((((Premature Birth[MeSH Terms]) OR (Birth, Premature[Title/Abstract])) OR (Births, Premature[Title/Abstract])) OR (Premature Births[Title/Abstract])) OR (Preterm Birth[Title/Abstract])) OR (Birth, Preterm[Title/Abstract])) OR (Birth, Preterm[Title/Abstract])) OR (Preterm Births[Title/Abstract]) |
|  | #3 | (#1) AND (#2) |
|  | #4 | (animals[MeSH Terms]) NOT (humans[MeSH Terms]) |
|  | #5 | (#3) NOT (#4) |
| EMBASE | #1 | (exp prematurity/ OR ("birth premature".ti,ab OR "infant, premature".ti,ab OR "infant, premature, diseases".ti,ab OR "neonate, premature".ti,ab OR "pre-mature birth".ti,ab OR "pre-mature infant".ti,ab OR "pre-maturity".ti,ab OR "pre-term babies".ti,ab OR "pre-term baby".ti,ab OR "pre-term birth".ti,ab OR "pre-term child".ti,ab OR "pre-term infant".ti,ab OR "pre-term infants".ti,ab OR "pre-term neonate".ti,ab OR "pre-term neonates".ti,ab OR "pre-term newborn".ti,ab OR "pre-term newborns".ti,ab OR "premature".ti,ab OR "premature babies".ti,ab OR "premature baby".ti,ab OR "premature birth".ti,ab OR "premature child".ti,ab OR "premature childbirth".ti,ab OR "premature infant".ti,ab OR "premature infant disease".ti,ab OR "premature infant diseases".ti,ab OR "premature infants".ti,ab OR "premature neonate".ti,ab OR "premature neonates".ti,ab OR "premature newborn".ti,ab OR "premature newborns".ti,ab OR "premature syndrome".ti,ab OR "prematuritas".ti,ab OR "prematurities".ti,ab OR "preterm babies".ti,ab OR "preterm baby".ti,ab OR "preterm birth".ti,ab OR "preterm child".ti,ab OR "preterm infant".ti,ab OR "preterm infants".ti,ab OR "preterm neonate".ti,ab OR "preterm neonates".ti,ab OR "preterm newborn".ti,ab OR "preterm newborns".ti,ab OR "prematurity".ti,ab)) |
|  | #2 | (exp hydrocortisone/ OR "11, 17 dihydroxy 17 (2 hydroxyacetyl) 10, 13 dimethyl 1, 2, 6, 7, 8, 9, 11, 12, 14, 15, 16 undecahydrocyclopenta [a] phenanthren 3 one".ti,ab OR "11, 17 dihydroxy 17 (2 hydroxyacetyl) 10, 13 dimethyl 2, 6, 7, 8, 9, 11, 12, 14, 15, 16 decahydro 1h cyclopenta [a] phenanthren 3 one".ti,ab OR "11beta, 17, 21 trihydroxypregn 4 ene 3, 20 dione".ti,ab OR "11beta, 17alpha, 21 trihydroxypregn 4 ene 3, 20 dione".ti,ab OR "14, 17 dihydroxy 14 (2 hydroxyacetyl) 2, 15 dimethyltetracyclo [8.7.0.0 (2, 7) .0 (11, 15)] heptadec 6 en 5 one".ti,ab OR "17 hydroxycorticosterone".ti,ab OR "4 pregnene 11beta, 17alpha, 21 triol 3, 20 dione".ti,ab OR "4 pregnene 3, 20 dione n beta, 17alpha, 21 triol".ti,ab OR "acticort".ti,ab OR "acticort 100".ti,ab OR "aeroseb hc".ti,ab OR "aeroseb-hc".ti,ab OR "ala-cort".ti,ab OR "ala-scalp".ti,ab OR "ala-scalp hp".ti,ab OR "alfacort".ti,ab OR "algicortis".ti,ab OR "alkindi".ti,ab OR "alkindi sprinkle".ti,ab OR "alpha derm".ti,ab OR "alphaderm".ti,ab OR "anucort-hc".ti,ab OR "anumed-hc".ti,ab OR "anutone-hc".ti,ab OR "aquanil hc".ti,ab OR "atrs 1902".ti,ab OR "atrs1902".ti,ab OR "balneol-hc".ti,ab OR "barseb hc".ti,ab OR "beta-hc".ti,ab OR "biacort".ti,ab OR "cetacort".ti,ab OR "chronocort".ti,ab OR "cobadex".ti,ab OR "colocort".ti,ab OR "compound f".ti,ab OR "cordicare lotion".ti,ab OR "coripen".ti,ab OR "cort dome".ti,ab OR "cort-dome".ti,ab OR "cort-dome high potency".ti,ab OR "cortef".ti,ab OR "cortef cream".ti,ab OR "cortenema".ti,ab OR "cortibel".ti,ab OR "corticorenol".ti,ab OR "cortifan".ti,ab OR "cortiphate".ti,ab OR "cortisol".ti,ab OR "cortisole".ti,ab OR "cortispray".ti,ab OR "cortoderm".ti,ab OR "cortril".ti,ab OR "cotacort".ti,ab OR "covocort".ti,ab OR "cremicort-h".ti,ab OR "cutaderm".ti,ab OR "derm-aid cream".ti,ab OR "dermacrin hc lotion".ti,ab OR "dermaid".ti,ab OR "dermaid soft cream".ti,ab OR "dermocare".ti,ab OR "dermocortal".ti,ab OR "dermolate".ti,ab OR "dioderm".ti,ab OR "eczacort".ti,ab OR "ef cortelan".ti,ab OR "efcortelan".ti,ab OR "efmody".ti,ab OR "egocort".ti,ab OR "egocort cream".ti,ab OR "eksalb".ti,ab OR "eldecort".ti,ab OR "emo-cort".ti,ab OR "epicort".ti,ab OR "ficortril".ti,ab OR "filocot".ti,ab OR "flexicort".ti,ab OR "gly-cort".ti,ab OR "glycort".ti,ab OR "gynecort".ti,ab OR "h-cort".ti,ab OR "hc (hydrocortisone)".ti,ab OR "hc no. 1".ti,ab OR "hc no. 4".ti,ab OR "hebcort".ti,ab OR "hebcort v".ti,ab OR "hemorrhoidal hc".ti,ab OR "hemril-30".ti,ab OR "hemril-hc uniserts".ti,ab OR "hi-cor".ti,ab OR "hidrotisona".ti,ab OR "hisone".ti,ab OR "hycor".ti,ab OR "hycort".ti,ab OR "hydracort".ti,ab OR "hydrasson".ti,ab OR "hydro ricortex".ti,ab OR "hydro-rx".ti,ab OR "hydrocort".ti,ab OR "hydrocorticosteroid".ti,ab OR "hydrocortisate".ti,ab OR "hydrocortison".ti,ab OR "hydrocortisone 1% in absorbase".ti,ab OR "hydrocortisone acetonide".ti,ab OR "hydrocortisone astier".ti,ab OR "hydrocortisone in absorbase".ti,ab OR "hydrocortisone ointment".ti,ab OR "hydrocortisone plus saline".ti,ab OR "hydrocortisone steroid".ti,ab OR "hydrocortisone, topical".ti,ab OR "hydrocortisonum".ti,ab OR "hydrocortisyl".ti,ab OR "hydrocortone".ti,ab OR "hydrogalen".ti,ab OR "hydrokort".ti,ab OR "hydrokortison".ti,ab OR "hydrotopic".ti,ab OR "hydventia".ti,ab OR "hysone".ti,ab OR "hytisone".ti,ab OR "hytone".ti,ab OR "hytone lotion".ti,ab OR "incortin h".ti,ab OR "infacort".ti,ab OR "instacort 10".ti,ab OR "kyypakkaus".ti,ab OR "lacticare hc".ti,ab OR "lacticare-hc".ti,ab OR "lemnis fatty cream hc".ti,ab OR "lenirit".ti,ab OR "medihaler cort".ti,ab OR "medihaler duo".ti,ab OR "medrocil".ti,ab OR "mildison".ti,ab OR "mildison fet krem".ti,ab OR "mildison lipocream".ti,ab OR "mildison-fatty".ti,ab OR "mitocortyl demangeaisons".ti,ab OR "munitren".ti,ab OR "nogenic hc".ti,ab OR "novohydrocort".ti,ab OR "nsc 10483".ti,ab OR "nsc 741".ti,ab OR "nsc10483".ti,ab OR "nutracort".ti,ab OR "optef".ti,ab OR "otosone f".ti,ab OR "penecort".ti,ab OR "plenadren".ti,ab OR "prepcort".ti,ab OR "prevex hc".ti,ab OR "procto-kit 1%".ti,ab OR "procto-kit 2.5%".ti,ab OR "proctocort".ti,ab OR "proctosert hc".ti,ab OR "proctosol-hc".ti,ab OR "proctosone".ti,ab OR "proctozone hc".ti,ab OR "procutan".ti,ab OR "rectasol-hc".ti,ab OR "rectocort".ti,ab OR "rederm".ti,ab OR "sanatison".ti,ab OR "scalp-aid".ti,ab OR "schericur".ti,ab OR "schericur 0.25%".ti,ab OR "scherosone f".ti,ab OR "sistral hydrocort".ti,ab OR "skincalm".ti,ab OR "stie-cort".ti,ab OR "substance m".ti,ab OR "synacort".ti,ab OR "texacort".ti,ab OR "triburon-hc".ti,ab OR "unicort".ti,ab OR "vasocort".ti,ab OR "hydrocortisone".ti,ab) |
|  | #3 | #1AND #2 |
| Cochrane Library | #1 | (Hydrocortisone):ti,ab,kw OR (Cortril):ti,ab,kw OR (Pregn-4-ene-3,20-dione, 11,17,21-trihydroxy-, (11beta)-):ti,ab,kw OR (Cortisol):ti,ab,kw OR (Hydrocortisone, (9 beta,10 alpha,11 alpha)-Isomer):ti,ab,kw OR (Cortifair):ti,ab,kw OR (Hydrocortisone, (11 alpha)-Isomer):ti,ab,kw OR (11 Epicortisol):ti,ab,kw OR (11-Epicortisol):ti,ab,kw OR (Epicortisol):ti,ab,kw OR (Cortef):ti,ab,kw |
|  | #2 | MeSH descriptor: [Hydrocortisone] explode all trees |
|  | #3 | #1 OR #2 |
|  | #4 | (Births, Premature):ti,ab,kw OR (Birth, Preterm):ti,ab,kw OR (Premature Births):ti,ab,kw OR (Births, Preterm):ti,ab,kw OR (Birth, Premature):ti,ab,kw OR (Preterm Birth):ti,ab,kw OR (Preterm Births):ti,ab,kw |
|  | #5 | MeSH descriptor: [Premature Birth] explode all trees |
|  | #6 | #4 OR #5 |
|  | #7 | #3 AND #6 |
| Web of Science | #1 | ((((((((AB=(parturition)) OR AB=(childbirth)) OR AB=(birth)) OR AB=(delivery)) OR AB=(deliver)) OR AB=(labor)) OR AB=(labour)) OR AB=(birth-giving)) OR AB=(partus) |
|  | #2 | ((((((((((AB=(Hydrocortisone)) OR AB=(Pregn-4-ene-3,20-dione, 11,17,21-trihydroxy-, (11beta)-)) OR AB=(Cortisol)) OR AB=(Cortifair)) OR AB=(Cortril)) OR AB=(Hydrocortisone, (9 beta,10 alpha,11 alpha)-Isomer)) OR AB=(Hydrocortisone, (11 alpha)-Isomer)) OR AB=(Epicortisol)) OR AB=(11-Epicortisol)) OR AB=(11 Epicortisol)) OR AB=(Cortef) |
|  | #3 | #1AND #2 |
| Scopus | #1 | ( ( ( TITLE-ABS-KEY ( premature AND birth ) OR TITLE-ABS-KEY ( birth, AND premature ) OR TITLE-ABS-KEY ( births, AND premature ) OR TITLE-ABS-KEY ( premature AND births ) OR TITLE-ABS-KEY ( preterm AND birth ) OR TITLE-ABS-KEY ( birth, AND preterm ) OR TITLE-ABS-KEY ( births, AND preterm ) OR TITLE-ABS-KEY ( preterm AND births ) ) ) AND ( ( TITLE-ABS-KEY ( hydrocortisone ) OR TITLE-ABS-KEY ( pregn4ene3,20dione, AND 11,17,21trihydroxy, AND 11beta ) OR TITLE-ABS-KEY ( cortisol ) OR TITLE-ABS-KEY ( cortifair ) OR TITLE-ABS-KEY ( cortril ) OR TITLE-ABS-KEY ( hydrocortisone, 9 beta,10 AND alpha,11 AND alpha AND isomer ) OR TITLE-ABS-KEY ( hydrocortisone, 11 alpha AND isomer ) OR TITLE-ABS-KEY ( epicortisol ) OR TITLE-ABS-KEY ( 11-epicortisol ) OR TITLE-ABS-KEY ( 11 epicortisol ) OR TITLE-ABS-KEY ( cortef ) ) ) ) AND NOT ( ( TITLE-ABS-KEY ( animal AND model ) OR TITLE-ABS-KEY ( animal AND models ) OR TITLE-ABS-KEY ( animal AND welfare ) ) ) |
| ScienceDirect | #1 | (Premature Birth OR Birth, Premature OR Births, Premature OR Premature Births OR Preterm Birth OR Birth, Preterm OR Births, Preterm OR Preterm Births) AND (Hydrocortisone OR Pregn-4-ene-3,20-dione, 11,17,21-trihydroxy-, (11beta)- OR Cortisol OR Cortifair OR Cortril OR Hydrocortisone, (9 beta,10 alpha,11 alpha)-Isomer OR Hydrocortisone, (11 alpha)-Isomer OR Epicortisol OR 11-Epicortisol OR 11 Epicortisol OR Cortef) |
| China National Knowledge Infrastructure (CNKI) | #1 | (主题: 早产儿) OR (主题: 未成熟儿) OR (主题: 早产) OR (主题: premature infant) OR (主题: premature infants) OR (主题: preterm infant) AND (主题: 氢化可的松) OR (主题: 皮质醇) OR (主题: 11-表皮质醇) OR (主题: 表皮质醇) OR (主题: Cortifair) OR (主题: Cortril) OR (主题: cortisol) OR (主题: hydrocortisone) |
| Chinese Biomedical Literature Database (CBM) | #1 | "早产儿"[常用字段:智能] OR "未成熟儿"[常用字段:智能] OR "早产"[常用字段:智能] OR "premature"[常用字段:智能] AND "infant"[常用字段:智能] OR "premature"[常用字段:智能] AND "infants"[常用字段:智能] OR "preterm"[常用字段:智能] AND "infant"[常用字段:智能] |
|  | #2 | "皮质醇"[常用字段:智能] OR "氢化可的松"[常用字段:智能] OR "cortisol"[常用字段:智能] OR "hydrocortisone"[常用字段:智能] |
|  | #3 | (#2) AND (#1) |
| Wan Fang database | #1 | (主题:(早产儿) or 题名或关键词:(早产儿 or 未成熟儿 or 早产 or premature infant or premature infants or preterm infant)) and (主题:(氢化可的松) or 题名或关键词:(氢化可的松 or 皮质醇 or 11-表皮质醇 or 表皮质醇 or Cortifair or Cortril or cortisol or hydrocortisone)) |
| Weipu (VIP) database | #1 | 题名或关键词=氢化可的松+cortisol+hydrocortisone+皮质醇+11-表皮质醇+表皮质醇+Cortifair+Cortril AND 题名或关键词=早产儿+premature infant+premature infants+preterm infant+未成熟儿+未成熟儿+早产 |

**Supplementary Table 1.** Search strategies for all database

| Domain | Item | Assessment criteria |
| --- | --- | --- |
| Selection | 1. Adequacy of case definition | a) yes, with independent validation  b) yes, e.g., record linkage or self-reports  c) no description |
|  | 2. Representativeness of cases | a) consecutive or obviously representative series  b) potential selection bias or not stated |
|  | 3. Selection of controls | a) community controls  b) hospital controls  c) no description |
|  | 4. Definition of controls | a) no history of disease (endpoint)  b) no description of source |
| Comparability | 1. Comparability of cases and controls | a) study controls for the most important factor  b) study controls for any additional important factor |
| Exposure | 1. Ascertainment of exposure | a) secure record  b) structured interview blind to case/control status  c) interview not blinded  d) written self-report or medical record only  e) no description |
|  | 2. Same ascertainment method for cases and controls | a) yes  b) no |
|  | 3. Non-response rate | a) same rate for both groups  b) non-respondents described  c) different rate with no designation |

**Supplementary Table 2.** Newcastle-Ottawa Scale (NOS) for Case-Control Studies. A study can be awarded a maximum of one star for each numbered item within the Selection and Outcome categories. A maximum of two stars can be given for Comparability.

| Domain | Item | Assessment criteria |
| --- | --- | --- |
| Selection | 1. Representativeness of exposed cohort | a) truly representative of the average community  b) somewhat representative  c) selected group (e.g., nurses, volunteers)  d) no description |
|  | 2. Selection of non-exposed cohort | a) drawn from the same community  b) drawn from a different source  c) no description |
|  | 3. Ascertainment of exposure | a) secure record  b) structured interview  c) written self-report  d) no description |
|  | 4. Outcome not present at start of study | a) yes  b) no |
| Comparability | 1. Comparability of cohorts | a) study controls for the most important factor  b) study controls for any additional important factor |
| Outcome | 1. Assessment of outcome | a) independent blind assessment  b) record linkage  c) self-report  d) no description |
|  | 2. Sufficient follow-up duration | a) yes  b) no |
|  | 3. Adequacy of follow-up | a) complete follow-up  b) low loss to follow-up, low bias  c) high loss to follow-up, no description  d) no statement |

**Supplementary Table 3.**Newcastle-Ottawa Scale (NOS) for Cohort Studies.A study can be awarded a maximum of one star for each numbered item within the Selection and Outcome categories. A maximum of two stars can be given for Comparability

| GWAS ID | Year | Trait | Consortium | Sample size | Number of SNPs |
| --- | --- | --- | --- | --- | --- |
| met-a-354 | 2014 | Cortisone | NA | 7575 | 2545383 |

**Supplementary Table 4:** Summary of the GWAS data used in the MR analyses

| **Author[Ref.]** | **Publish  Year** | **Couontry** | **EL** | **Q** | **Specimen** | **Case numbers** | | **Cortisol levels(ug/dl)  [mean(or median)± SD]** | | **GA** | **Measurement  age** | **Measurement  time** | **Prenatal corticosteroid use** | **Postnatal corticosteroid use** | **Measurement  method** |
| --- | --- | --- | --- | --- | --- | --- | --- | --- | --- | --- | --- | --- | --- | --- | --- |
|  |  |  |  |  |  | **Pre**  **term** | **Term** | **Preterm** | **Term** |  |  |  |  |  |  |
| Murphy B.et al | 1974.8 | Canada | 4 | 7 | UCB | 21 | 18 | 4.680±3.987 | 9.400±11.750 | 27-37w | at birth | - | No | No | FIA |
| Sybulski S.et al | 1976.5 | Canada | 4 | 7 | UCB | 67 | 283 | 5.715±1.005 | 6.841±0.927 | 26-37w | at birth | - | No | No | ELISA |
| Blumenthal N.et al | 1983.4 | South African | 4 | 7 | UCB | 29 | 40 | 192000±192000 | 193000±92000 | 31.5±1.7w | at birth | - | No | No | RIA |
| Shen X.et al | 1988.7 | China | 4 | 7 | UCB | 11 | 31 | 50400±10800 | 61200±7920 | - | at birth | - | No | No | RIA |
| Simon T.et al | 2018.3 | France | 3 | 7 | PB | 11 | 31 | 7.915±7.581 | 2.321±7.085 | ＜32w | 3d | - | No | Not reported | LC-MS/MS |
|  |  |  |  |  | PB | 28 | 31 | 2.937±4.350 | 2.321±7.085 | 33-36w | 3d | - | No | Not reported |  |
|  |  |  |  |  | UCB | 46 | 39 | 1.009±0.803 | 3.870±2.543 | ＜32w | at birth | - | No | Not reported |  |
|  |  |  |  |  | UCB | 67 | 39 | 1.671±1.405 | 3.870±2.543 | 33-36w | at birth | - | No | Not reported |  |
| Kurki T.et al | 1991.7 | Finland | 4 | 8 | UCB | 12 | 11 | 13.644±27.385 | 11.988±11.812 | - | at birth | - | No | No | RIA |
| Srivastava T.et al | 1994.8 | India | 4 | 8 | UCB | 42 | 47 | 8.590±5.440 | 11.670±4.680 | ＜34w | at birth | - | No | No | RIA |
|  |  |  |  |  | UCB | 32 | 47 | 8.280±7.400 | 11.670±4.680 | ＜34w | at birth | - | Yes | No |  |
| Li Z.et al | 1994.9 | China | 4 | 8 | UCB | 42 | 264 | 7.920±5.400 | 7.488±5.256 | - | at birth | - | No | Not reported | RIA |
| Elizabeth T.et al | 2004.8 | USA | 4 | 8 | UCB | 39 | 91 | 0.040±0.020 | 0.043±0.021 | - | at birth | - | Not reported | Not reported | ELISA |
| Masahiro M.et al | 2005.11 | Japan | 4 | 8 | UCB | 4 | 11 | 8.022±4.192 | 11.440±4.880 | ＜28w | at birth | - | No | Not reported | LC-MS/MS |
|  |  |  |  |  | UCB | 8 | 11 | 3.820±2.580 | 11.440±4.880 | 29-32w | at birth | - | No | Not reported |  |
|  |  |  |  |  | UCB | 5 | 11 | 4.840±1.900 | 11.440±4.880 | 33-36w | at birth | - | No | Not reported |  |
| Ding Y.et al | 2007.4 | China | 4 | 7 | UCB | 26 | 29 | 24.626±11.729 | 15.065±7.013 | 33.20±1.40w | at birth | - | Not reported | Not reported | RIA |
| Thi T.et al | 2009 | Australia | 4 | 8 | UCB | 33 | 53 | 0.982±1.482 | 7.651±7.004 | 24-36w | at birth | - | Yes | Not reported | RIA |
| Afzal A.et al | 2016.5 | India | 4 | 8 | UCB | 19 | 19 | 8.100±3.230 | 12.610±3.300 | ＜32w | at birth | - | Not reported | Not reported | ELISA |
|  |  |  |  |  | UCB | 20 | 19 | 12.270±2.770 | 12.610±3.300 | 33-36w | at birth | - | Not reported | Not reported |  |
| Afzal A.et al | 2016.11 | India | 4 | 7 | UCB | 35 | 35 | 8.900±4.600 | 11.880±5.780 | 26.37±2.25 | at birth | - | Not reported | Not reported | ELISA |
| Braun T.et al | 2018.11 | Germany | 4 | 8 | UCB | 14 | 30 | 25.407±7.318 | 33.798±12.745 | 24-37w | at birth | - | No | Not reported | ELISA |
|  |  |  |  |  | UCB | 28 | 19 | 29.093±17.027 | 39.643±13.958 | 24-37w | at birth | - | Yes | Not reported |  |
| Rabiepoor R.et al | 2019.12 | Iran | 4 | 7 | UCB | 4 | 106 | 2.743±0.423 | 2.230±0.226 | - | at birth | - | No | Not reported | ELISA |
| Ravivarma R.et al | 2022.12 | India | 4 | 7 | UCB | 84 | 132 | 10.600±4.444 | 12.000±7.407 | 24-36w | at birth | - | Not reported | Not reported | ELISA |
| Huang M.et al | 1998.4 | China | 4 | 5 | PB | 30 | 30 | 10.500±1.500 | 18.100±1.500 | - | 1d | noon | Not reported | No | RIA |
|  |  |  |  |  | PB | 30 | 30 | 10.000±1.100 | 13.400±1.200 | - | 2d | noon | Not reported | No |  |
|  |  |  |  |  | PB | 30 | 30 | 5.100±0.500 | 8.200±2.100 | - | 3d | noon | Not reported | No |  |
|  |  |  |  |  | PB | 30 | 30 | 4.000±1.800 | 5.800±0.500 | - | 4d | noon | Not reported | No |  |
|  |  |  |  |  | PB | 30 | 30 | 4.000±1.500 | 5.600±0.800 | - | 5d | noon | Not reported | No |  |
| Luo X.et al | 1988.4 | China | 4 | 5 | PB | 20 | 20 | 151.127±107.001 | 77.998±23.658 | - | 1-3d | noon | Not reported | Not reported | RIA |
| Doerr H.et al | 1988.5 | Germany | 3 | 6 | PB | 8 | 12 | 11.800±1.697 | 9.760±0.139 | 33-36w | 1d | - | No | No | RIA |
|  |  |  |  |  | UCB | 8 | 12 | 4.270±0.366 | 4.550±0.599 | 33-36w | at birth | - | No | No |  |
| Zhao M.et al | 1989.8 | China | 4 | 6 | PB | 42 | 36 | 27.371±15.419 | 37.149±17.882 | 33-36w | 24hours after birth | moring | Not reported | Not reported | RIA |
|  |  |  |  |  | PB | 35 | 30 | 10.587±6.772 | 18.500±9.814 | 33-36w | 72hours after birth | moring | Not reported | Not reported |  |
|  |  |  |  |  | PB | 31 | 22 | 6.850±3.873 | 7.898±4.296 | 33-36w | 120hours after birth | moring | Not reported | Not reported |  |
| Lee M.et al | 1989.12 | USA | 4 | 6 | PB | 13 | 16 | 6.840±1.044 | 6.156±0.972 | 31-35w | 2-5d | - | Not reported | Not reported | RIA |
| Zhao M.et al | 1990.8 | China | 4 | 5 | PB | 21 | 36 | 608.400±313.452 | 766.800±388.800 | - | 1d | moring | Not reported | Not reported | RIA |
|  |  |  |  |  | PB | 13 | 30 | 345.600±181.728 | 417.600±217.080 | - | 3d | moring | Not reported | Not reported |  |
|  |  |  |  |  | PB | 13 | 21 | 176.400±77.868 | 219.600±132.120 | - | 5d | moring | Not reported | Not reported |  |
|  |  |  |  |  | PB | 10 | 10 | 162.000±113.760 | 176.400±91.080 | - | 7d | moring | Not reported | Not reported |  |
| W Rokicki.et al | 1990 | Poland | 4 | 6 | PB | 6 | 5 | 14.420±2.900 | 12.200±6.500 | - | 0-6hours after birth | - | Not reported | Not reported | RIA |
|  |  |  |  |  | PB | 3 | 7 | 25.090±18.600 | 4.100±1.000 | - | 7-12hours after birth | - | Not reported | Not reported |  |
|  |  |  |  |  | PB | 4 | 6 | 11.600±7.000 | 2.580±0.800 | - | 13-24hours after birth | - | Not reported | Not reported |  |
|  |  |  |  |  | PB | 5 | 16 | 2.930±1.900 | 1.870±9.900 | - | 2d | - | Not reported | Not reported |  |
|  |  |  |  |  | PB | 15 | 19 | 9.150±7.100 | 2.290±0.800 | - | 3-5d | - | Not reported | Not reported |  |
|  |  |  |  |  | PB | 23 | 10 | 6.210±4.700 | 4.870±1.900 | - | 6-12d | - | Not reported | Not reported |  |
|  |  |  |  |  | PB | 21 | 6 | 3.070±2.060 | 4.370±2.500 | - | 13-30d | - | Not reported | Not reported |  |
|  |  |  |  |  | PB | 8 | 3 | 2.650±1.100 | 5.560±2.000 | - | 2 months | moring | Not reported | Not reported |  |
|  |  |  |  |  | PB | 3 | 3 | 5.050±5.000 | 9.630±3.100 | - | 3 months | moring | Not reported | Not reported |  |
| Zhu X. et al | 1992.6 | China | 3 | 6 | PB | 40 | 41 | 12.352±6.847 | 18.281±7.927 | 34-36w | 1d | moring | Not reported | Not reported | RIA |
|  |  |  |  |  | PB | 40 | 41 | 11.984±7.175 | 13.640±3.229 | 34-36w | 2d | moring | Not reported | Not reported |  |
|  |  |  |  |  | PB | 40 | 41 | 8.197±4.669 | 8.345±3.704 | 34-36w | 3-4d | moring | Not reported | Not reported |  |
|  |  |  |  |  | PB | 40 | 41 | 6.379±5.195 | 5.029±2.801 | 34-36w | 5-7d | moring | Not reported | Not reported |  |
|  |  |  |  |  | PB | 35 | 41 | 18.216±14.875 | 18.281±7.927 | 28-33w | 1d | moring | Not reported | Not reported |  |
|  |  |  |  |  | PB | 36 | 42 | 19.534±11.974 | 13.640±3.229 | 28-33w | 2d | moring | Not reported | Not reported |  |
|  |  |  |  |  | PB | 37 | 43 | 12.690±12.928 | 8.345±3.704 | 28-33w | 3-4d | moring | Not reported | Not reported |  |
|  |  |  |  |  | PB | 38 | 44 | 10.274±6.628 | 5.029±2.801 | 28-33w | 5-7d | moring | Not reported | Not reported |  |
| Economou G. et al | 1993.5 | Greece | 3 | 8 | PB | 15 | 15 | 8.640±4.176 | 6.840±4.176 | 33.5±1.5w | 1d | moring | Not reported | No | FIA |
|  |  |  |  |  | PB | 15 | 15 | 9.720±4.176 | 7.200±4.176 | 33.5±1.5w | 1d | evening | Not reported | No |  |
|  |  |  |  |  | PB | 15 | 15 | 8.640±5.580 | 5.400±4.176 | 33.5±1.5w | 1d | afternoon | Not reported | No |  |
|  |  |  |  |  | PB | 15 | 15 | 8.280±2.772 | 6.840±5.580 | 33.5±1.5w | 1d | early morning | Not reported | No |  |
|  |  |  |  |  | PB | 15 | 15 | 6.840±2.772 | 7.560±2.772 | 33.5±1.5w | 3d | moring | Not reported | No |  |
|  |  |  |  |  | PB | 15 | 15 | 6.480±2.772 | 3.600±1.404 | 33.5±1.5w | 3d | evening | Not reported | No |  |
|  |  |  |  |  | PB | 15 | 15 | 7.200±6.984 | 4.680±4.176 | 33.5±1.5w | 3d | afternoon | Not reported | No |  |
|  |  |  |  |  | PB | 15 | 15 | 3.240±2.772 | 4.680±5.580 | 33.5±1.5w | 3d | early morning | Not reported | No |  |
|  |  |  |  |  | PB | 15 | 15 | 11.880±5.580 | 6.120±5.580 | 33.5±1.5w | 5d | moring | Not reported | No |  |
|  |  |  |  |  | PB | 15 | 15 | 8.280±2.772 | 3.600±2.772 | 33.5±1.5w | 5d | evening | Not reported | No |  |
|  |  |  |  |  | PB | 15 | 15 | 6.480±4.176 | 5.400±4.176 | 33.5±1.5w | 5d | afternoon | Not reported | No |  |
|  |  |  |  |  | PB | 15 | 15 | 3.600±2.772 | 4.320±16.74 | 33.5±1.5w | 5d | early morning | Not reported | No |  |
|  |  |  |  |  | PB | 15 | 15 | 11.880±4.176 | 4.320±5.580 | 33.5±1.5w | 10d | moring | Not reported | No |  |
|  |  |  |  |  | PB | 15 | 15 | 7.560±6.840 | 2.520±1.404 | 33.5±1.5w | 10d | evening | Not reported | No |  |
|  |  |  |  |  | PB | 15 | 15 | 6.120±5.580 | 3.960±5.580 | 33.5±1.5w | 10d | afternoon | Not reported | No |  |
|  |  |  |  |  | PB | 15 | 15 | 5.760±4.176 | 3.240±2.772 | 33.5±1.5w | 10d | early morning | Not reported | No |  |
|  |  |  |  |  | PB | 15 | 15 | 12.960±4.176 | 2.880±4.176 | 33.5±1.5w | 15d | moring | Not reported | No |  |
|  |  |  |  |  | PB | 15 | 15 | 8.640±6.984 | 2.880±1.404 | 33.5±1.5w | 15d | evening | Not reported | No |  |
|  |  |  |  |  | PB | 15 | 15 | 2.880±1.404 | 2.520±2.772 | 33.5±1.5w | 15d | afternoon | Not reported | No |  |
|  |  |  |  |  | PB | 15 | 15 | 5.400±4.176 | 1.080±1.404 | 33.5±1.5w | 15d | early morning | Not reported | No |  |
|  |  |  |  |  | PB | 15 | 15 | 6.840±4.176 | 5.040±4.176 | 33.5±1.5w | 1 months | moring | Not reported | No |  |
|  |  |  |  |  | PB | 15 | 15 | 5.400±2.772 | 2.880±4.176 | 33.5±1.5w | 1 months | evening | Not reported | No |  |
|  |  |  |  |  | PB | 15 | 15 | 2.880±2.772 | 3.240±4.176 | 33.5±1.5w | 1 months | afternoon | Not reported | No |  |
|  |  |  |  |  | PB | 15 | 15 | 6.840±4.176 | 1.440±1.404 | 33.5±1.5w | 1 months | early morning | Not reported | No |  |
| Metzger D.et al | 1993.8 | USA | 4 | 8 | PB | 5 | 5 | 12.700±4.700 | 10.000±1.900 | 30±2w | 2 months | afternoon | No | No | RIA |
| Zhu X. et al | 1993.10 | China | 3 | 6 | PB | 40 | 41 | 13.570±7.150 | 20.880±9.290 | 34-36w | 2hours after birth | - | No | Not reported | RIA |
|  |  |  |  |  | PB | 40 | 41 | 11.280±6.600 | 15.910±5.580 | 34-36w | 12hours after birth | - | No | Not reported |  |
|  |  |  |  |  | PB | 40 | 41 | 12.060±7.220 | 13.730±3.250 | 34-36w | 2d | moring | No | Not reported |  |
|  |  |  |  |  | PB | 40 | 41 | 8.690±4.400 | 8.480±4.560 | 34-36w | 3d | moring | No | Not reported |  |
|  |  |  |  |  | PB | 40 | 41 | 7.810±4.820 | 8.330±2.730 | 34-36w | 4d | moring | No | Not reported |  |
|  |  |  |  |  | PB | 40 | 41 | 7.520±7.390 | 4.420±2.990 | 34-36w | 5d | moring | No | Not reported |  |
|  |  |  |  |  | PB | 40 | 41 | 6.420±4.270 | 5.310±2.370 | 34-36w | 6d | moring | No | Not reported |  |
|  |  |  |  |  | PB | 40 | 41 | 5.420±3.550 | 5.280±3.330 | 34-36w | 7d | moring | No | Not reported |  |
|  |  |  |  |  | PB | 40 | 41 | 6.130±4.740 | 5.670±2.220 | 34-36w | ＞7d | moring | No | Not reported |  |
|  |  |  |  |  | PB | 35 | 41 | 13.350±8.650 | 20.880±9.290 | 28-33w | 2hours after birth | - | No | Not reported |  |
|  |  |  |  |  | PB | 35 | 41 | 21.320±19.170 | 15.910±5.580 | 28-33w | 12hours after birth | - | No | Not reported |  |
|  |  |  |  |  | PB | 35 | 41 | 19.660±12.050 | 13.730±3.250 | 28-33w | 2d | moring | No | Not reported |  |
|  |  |  |  |  | PB | 35 | 41 | 12.570±18.470 | 8.480±4.560 | 28-33w | 3d | moring | No | Not reported |  |
|  |  |  |  |  | PB | 35 | 41 | 12.980±6.780 | 8.330±2.730 | 28-33w | 4d | moring | No | Not reported |  |
|  |  |  |  |  | PB | 35 | 41 | 8.000±3.350 | 4.420±2.990 | 28-33w | 5d | moring | No | Not reported |  |
|  |  |  |  |  | PB | 35 | 41 | 11.270±5.300 | 5.310±2.370 | 28-33w | 6d | moring | No | Not reported |  |
|  |  |  |  |  | PB | 35 | 41 | 11.340±9.320 | 5.280±3.330 | 28-33w | 7d | moring | No | Not reported |  |
|  |  |  |  |  | PB | 35 | 41 | 6.380±3.920 | 5.670±2.220 | 28-33w | ＞7d | moring | No | Not reported |  |
| Ng P.et al | 2000.11 | China | 3 | 8 | PB | 43 | 43 | 5.220±5.918 | 10.728±5.602 | 29.60±1.78w | 1d | - | Partial | Not reported | RIA |
|  |  |  |  |  | PB | 54 | 43 | 8.208±5.652 | 10.728±5.602 | 34.20±1.48w | 1d | - | Partial | Not reported |  |
|  |  |  |  |  | PB | 43 | 43 | 7.236±5.947 | 4.824±4.532 | 29.60±1.78w | 4-5d | - | Partial | Not reported |  |
|  |  |  |  |  | PB | 54 | 43 | 8.748±5.252 | 4.824±4.532 | 34.20±1.48w | 4-5d | - | Partial | Not reported |  |
| Jefferies C.et al | 2004.3 | New Zealand | 4 | 6 | PB | 14 | 40 | 9.396±4.579 | 12.186±9.797 | 28.40±0.6w | 7d | - | Not reported | Not reported | FIA |
| Sun Z.et al | 2004.6 | China | 4 | 6 | PB | 40 | 30 | 19.508±6.440 | 37.303±9.532 | ＜34w | 1d | moring | Not reported | Not reported | FIA |
|  |  |  |  |  | PB | 40 | 30 | 8.226±2.094 | 7.043±1.768 | ＜34w | 7d | moring | Not reported | Not reported |  |
|  |  |  |  |  | PB | 40 | 30 | 6.280±2.566 | 7.141±1.753 | ＜34w | 14d | moring | Not reported | Not reported |  |
|  |  |  |  |  | PB | 40 | 30 | 13.767±5.221 | 37.303±9.532 | ≥34w | 1d | moring | Not reported | Not reported |  |
|  |  |  |  |  | PB | 40 | 30 | 6.726±2.169 | 7.043±1.768 | ≥34w | 7d | moring | Not reported | Not reported |  |
|  |  |  |  |  | PB | 40 | 30 | 6.522±1.937 | 7.141±1.753 | ≥34w | 14d | moring | Not reported | Not reported |  |
| Cai L.et al | 2005.5 | China | 4 | 8 | PB | 65 | 65 | 18.370±7.770 | 22.840±10.820 | - | day1 in hospitalization | moring | No | No | RIA |
|  |  |  |  |  | PB | 65 | 65 | 16.980±8.900 | 17.350±12.600 | - | the recovery period | moring | No | No |  |
| He X.et al | 2007.2 | China | 4 | 8 | PB | 81 | 80 | 18.410±7.680 | 22.910±10.860 | - | day1 in hospitalization | moring | No | No | RIA |
|  |  |  |  |  | PB | 81 | 80 | 16.880±8.870 | 17.650±12.630 | - | the recovery period | moring | No | No |  |
| M.G.Ballerini.et al | 2010.1 | Argentina | 4 | 5 | PB | 21 | 126 | 4.800±4.850 | 4.100±9.694 | ≤32w | 1 months | - | Not reported | Not reported | FIA |
|  |  |  |  |  | PB | 17 | 126 | 4.200±5.918 | 4.100±9.694 | ＞32w | 1 months | - | Not reported | Not reported |  |
| Chen C.et al | 2012.3 | China | 4 | 8 | PB | 40 | 15 | 0.023±0.017 | 0.023±0.015 | - | 30min after birth | - | No | Not reported | ELISA |
| Ge Y.et al | 2012.11 | China | 4 | 7 | PB | 154 | 154 | 13.240±4.150 | 15.820±5.524 | - | 1d | moring | Not reported | Not reported | FIA |
|  |  |  |  |  | PB | 154 | 154 | 10.729±3.354 | 12.932±3.229 | - | 3d | moring | Not reported | Not reported |  |
|  |  |  |  |  | PB | 154 | 154 | 12.057±3.668 | 10.529±3.020 | - | 7d | moring | Not reported | Not reported |  |
|  |  |  |  |  | PB | 154 | 154 | 11.453±3.670 | 9.828±3.227 | - | 14d | moring | Not reported | Not reported |  |
| Zhu W.et al | 2013.6 | China | 4 | 7 | PB | 32 | 26 | 18.800±6.600 | 19.500±6.400 | 32.00±1.3w | 1d | moring | No | Not reported | FIA |
|  |  |  |  |  | PB | 32 | 26 | 16.400±5.500 | 18.600±5.200 | 32.00±1.3w | 7d | moring | No | Not reported |  |
| Nina K.et al | 2014.1 | Finland | 3 | 7 | Saliva | 54 | 40 | 0.252±0.061 | 0.220±0.072 | 29.4±2.3w | 23 years old | pre-trial | Not reported | Partial | FIA |
|  |  |  |  |  | PB | 54 | 40 | 11.711±0.050 | 10.883±0.065 | 29.4±2.3w | 23 years old | pre-trial | Not reported | Partial |  |
| Mei G.et al | 2014.6 | China | 4 | 7 | PB | 14 | 52 | 0.021±0.008 | 0.013±0.006 | ＜32w | 1d | moring | No | No | FIA |
|  |  |  |  |  | PB | 14 | 52 | 0.016±0.007 | 0.012±0.004 | ＜32w | 3d | moring | No | No |  |
|  |  |  |  |  | PB | 14 | 52 | 0.012±0.005 | 0.011±0.004 | ＜32w | 7d | moring | No | No |  |
|  |  |  |  |  | PB | 21 | 52 | 0.008±0.004 | 0.013±0.006 | 34-36w | 1d | moring | No | No |  |
|  |  |  |  |  | PB | 21 | 52 | 0.010±0.005 | 0.012±0.004 | 34-36w | 3d | moring | No | No |  |
|  |  |  |  |  | PB | 21 | 52 | 0.011±0.005 | 0.011±0.004 | 34-36w | 7d | moring | No | No |  |
|  |  |  |  |  | PB | 34 | 52 | 0.009±0.005 | 0.013±0.006 | 34-36w | 1d | moring | No | No |  |
|  |  |  |  |  | PB | 34 | 52 | 0.011±0.005 | 0.012±0.004 | 34-36w | 3d | moring | No | No |  |
|  |  |  |  |  | PB | 34 | 52 | 0.012±0.004 | 0.011±0.004 | 34-36w | 7d | moring | No | No |  |
| A Kistner.et al | 2014.11 | Sweden | 4 | 7 | PB | 28 | 29 | 25.400±10.574 | 33.900±13.539 | ＜32w | 9 years old | moring | Not reported | Not reported | FIA |
| Cha P.et al | 2015.18 | China | 4 | 8 | PB | 52 | 17 | 7.182±9.065 | 4.320±3.924 | - | 3d | moring | No | No | FIA |
|  |  |  |  |  | PB | 52 | 17 | 5.040±5.839 | 3.708±4.118 | - | 7d | moring | No | No |  |
| Yang R.et al | 2018.8 | China | 4 | 7 | PB | 79 | 95 | 0.012±0.004 | 0.011±0.006 | - | 1d | - | No | No | FIA |
| Miranda D.et al | 2020.3 | Netherlands | 4 | 7 | PB | 41 | 64 | 7.200±5.198 | 8.748±3.200 | 27.90±1.3w | 2 years old | afternoon | Partial | Partial | LC-MS/MS |
| Arsinoi K.et al | 2021.12 | Greece | 4 | 7 | PB | 87 | 73 | 8.600±5.185 | 8.500±5.111 | 31.30±3.2w | 10 years old | pre-trial | Not reported | Not reported | FIA |
| Marta O.et al | 2023.5 | Poland | 3 | 8 | Saliva | 21 | 30 | 0.220±0.276 | 0.176±0.192 | 33-35w | 16d±8d | moring | Not reported | Not reported | RIA |
|  |  |  |  |  | Saliva | 20 | 30 | 0.213±0.158 | 0.176±0.192 | 27-30w | 57±22d | moring | Not reported | Not reported |  |
|  |  |  |  |  | Saliva | 21 | 30 | 0.190±0.200 | 0.220±0.109 | 33-35w | 16d±8d | pre-trial 1 | Not reported | Not reported |  |
|  |  |  |  |  | Saliva | 20 | 30 | 0.188±0.197 | 0.220±0.109 | 27-30w | 57±22d | pre-trial 1 | Not reported | Not reported |  |
|  |  |  |  |  | Saliva | 21 | 30 | 0.216±0.121 | 0.280±0.134 | 33-35w | 16d±8d | pre-trial 2 | Not reported | Not reported |  |
|  |  |  |  |  | Saliva | 20 | 30 | 0.319±1.140 | 0.280±0.134 | 27-30w | 57±22d | pre-trial 2 | Not reported | Not reported |  |
| Katrin I.et al | 2012.3 | Sweden | 4 | 7 | Saliva | 11 | 11 | 0.554±0.533 | 0.490±0.302 | 27-32w | aroud 28d | pre-trial | Partial | No | RIA |
| David W.et al | 2006.1 | Canada | 4 | 7 | Saliva | 21 | 17 | 0.200±0.108 | 0.320±0.108 | 28±2.89w | 3 months | pre-trial | No | Not reported | ELISA |
| Ruth E.et al | 2007.2 | Canada | 3 | 8 | Saliva | 34 | 73 | 0.199±0.029 | 0.292±0.019 | 26.5±1.5w | 3 months | noon | Partial | Partial | ELISA |
|  |  |  |  |  | Saliva | 59 | 73 | 0.231±0.022 | 0.292±0.019 | 31.1±1.3w | 3 months | noon | Partial | Partial |  |
|  |  |  |  |  | Saliva | 29 | 66 | 0.231±0.034 | 0.194±0.022 | 26.5±1.5w | 6 months | noon | Partial | Partial |  |
|  |  |  |  |  | Saliva | 65 | 66 | 0.218±0.027 | 0.194±0.022 | 31.1±1.3w | 6 months | noon | Partial | Partial |  |
|  |  |  |  |  | Saliva | 40 | 51 | 0.248±0.025 | 0.191±0.023 | 26.5±1.5w | 8 months | noon | Partial | Partial |  |
|  |  |  |  |  | Saliva | 48 | 51 | 0.164±0.025 | 0.191±0.023 | 31.1±1.3w | 8 months | noon | Partial | Partial |  |
|  |  |  |  |  | Saliva | 43 | 40 | 0.215±0.024 | 0.163±0.023 | 26.5±1.5w | 18 months | noon | Partial | Partial |  |
|  |  |  |  |  | Saliva | 38 | 40 | 0.109±0.025 | 0.163±0.023 | 31.1±1.3w | 18 months | noon | Partial | Partial |  |
| David W.et al | 2008.11 | UK | 4 | 7 | Saliva | 55 | 49 | 0.200±0.150 | 0.320±0.280 | 29.17±2.51w | 3 months | pre-trial | No | Not reported | ELISA |
| Katrin I.et al | 2015.3 | Germany | 3 | 7 | Saliva | 61 | 30 | 0.410±0.330 | 0.270±0.160 | 27.60±2.80w | 3 months | pre-trial | Partial | Partial | RIA |
|  |  |  |  |  | Saliva | 30 | 30 | 0.320±0.250 | 0.270±0.160 | 34.20±1.40w | 3 months | pre-trial | Partial | Partial |  |
| Livio P.et al | 2016.10 | Italy | 4 | 7 | Saliva | 37 | 53 | 1.288±0.274 | 1.270±0.350 | ≤32w | 3 months | noon | Not reported | Not reported | ELISA |
| Bettendorf M.et al | 1998.4 | Germany | 3 | 8 | Saliva | 10 | 10 | 0.184±0.141 | 0.799±0.533 | 26-31w | 15~39d | D1 moring | No | No | RIA |
|  |  |  |  |  | Saliva | 10 | 10 | 0.198±0.080 | 0.396±0.401 | 26-31w | 15~39d | D2 moring | No | No |  |
|  |  |  |  |  | Saliva | 10 | 10 | 0.202±0.414 | 0.187±0.352 | 26-31w | 15~39d | D3 moring | No | No |  |
|  |  |  |  |  | Saliva | 10 | 10 | 0.155±0.176 | 0.212±0.228 | 26-31w | 15~39d | D1 noon | No | No |  |
|  |  |  |  |  | Saliva | 10 | 10 | 0.140±0.081 | 0.328±0.109 | 26-31w | 15~39d | D2 noon | No | No |  |
|  |  |  |  |  | Saliva | 10 | 10 | 0.209±0.174 | 0.187±0.208 | 26-31w | 15~39d | D3 noon | No | No |  |
|  |  |  |  |  | Saliva | 10 | 10 | 0.342±0.218 | 0.511±0.245 | 26-31w | 15~39d | D1 afternoon | No | No |  |
|  |  |  |  |  | Saliva | 10 | 10 | 0.252±0.151 | 0.223±0.141 | 26-31w | 15~39d | D2 afternoon | No | No |  |
|  |  |  |  |  | Saliva | 10 | 10 | 0.223±0.116 | 0.252±0.167 | 26-31w | 15~39d | D3 afternoon | No | No |  |
|  |  |  |  |  | Saliva | 10 | 10 | 0.119±0.459 | 0.364±0.491 | 26-31w | 15~39d | D1 evening | No | No |  |
|  |  |  |  |  | Saliva | 10 | 10 | 0.180±0.113 | 0.158±0.221 | 26-31w | 15~39d | D2 evening | No | No |  |
|  |  |  |  |  | Saliva | 10 | 10 | 0.180±0.084 | 0.173±0.185 | 26-31w | 15~39d | D3 evening | No | No |  |
| David Q.et al | 2022.11 | UK | 3 | 8 | Saliva | 56 | 45 | 0.121±0.226 | 0.097±0.108 | 29.5–31.3w | 4 months | pre-trial | No | No | LC-MS/MS |
|  |  |  |  |  | Saliva | 14 | 42 | 0.155±0.086 | 0.281±0.094 | ＜28w | 4 months | moring | No | No |  |
|  |  |  |  |  | Saliva | 34 | 42 | 0.144±0.086 | 0.281±0.094 | 28~32w | 4 months | moring | No | No |  |
|  |  |  |  |  | Saliva | 14 | 42 | 0.126±0.086 | 0.061±0.083 | ＜28w | 4 months | evening | No | No |  |
|  |  |  |  |  | Saliva | 34 | 42 | 0.047±0.083 | 0.061±0.083 | 28-32w | 4 months | evening | No | No |  |
| Ruth E.et al | 2010.1 | Canada | 4 | 8 | Saliva | 29 | 32 | 0.277±0.088 | 0.298±0.072 | 26.7±1.7w | 4 months | pre-trial | Not reported | Not reported | ELISA |
|  |  |  |  |  | Saliva | 39 | 32 | 0.286±0.082 | 0.298±0.072 | 31.3±1.1w | 4 months | pre-trial | Not reported | Not reported |  |
| Sarah J.et al | 2013.6 | USA | 4 | 8 | Saliva | 29 | 24 | 0.210±0.013 | 0.280±0.130 | 27.93±1.89w | 6~8 months | pre-trial | No | Not reported | ELISA |
| Stephanie H.et al | 2014.12 | Switzerland | 3 | 8 | Saliva | 56 | 25 | 0.858±0.389 | 0.977±0.310 | 30.14±2.01w | 6 months | moring | Not reported | Not reported | ELISA |
|  |  |  |  |  | Saliva | 56 | 25 | 0.783±0.284 | 0.835±0.306 | 30.14±2.01w | 6 months | noon | Not reported | Not reported |  |
|  |  |  |  |  | Saliva | 56 | 25 | 0.531±0.389 | 0.674±0.330 | 30.14±2.01w | 6 months | afternoon | Not reported | Not reported |  |
|  |  |  |  |  | Saliva | 56 | 25 | 0.270±0.427 | 0.498±0.355 | 30.14±2.01w | 6 months | evening | Not reported | Not reported |  |
| Mai T.et al | 2007.3 | Canada | 4 | 8 | Saliva | 84 | 37 | 0.200±0.200 | 0.200±0.200 | 29.30±2.7w | 8 months | moring | Partial | Partial | ELISA |
| Marta O.et al | 2023 | Poland | 4 | 8 | Saliva | 17 | 21 | 0.244±0.181 | 0.215±0.278 | 33-35w | 18±7 d | moring | Partial | Partial | RIA |
|  |  |  |  |  | Saliva | 19 | 21 | 0.383±0.529 | 0.215±0.278 | 26-30w | 55±21 d | moring | Partial | Partial |  |
| Lorna G.et al | 2022.7 | UK | 4 | 8 | Saliva | 20 | 24 | 0.087±0.066 | 0.058±0.049 | 28-32w | 9 months | pre-trial | Not reported | Not reported | LC-MS/MS |
| Susanne B.et al | 2011.3 | Canada | 3 | 8 | Saliva | 25 | 22 | 0.212±0.029 | 0.118±0.031 | 26.1±1.4w | 18 months | moring | Partial | No | ELISA |
|  |  |  |  |  | Saliva | 26 | 22 | 0.126±0.027 | 0.118±0.031 | 31.2±1.2w | 18 months | moring | Partial | No |  |
| M.A. McLean.et al | 2023.5 | Canada | 4 | 7 | Saliva | 91 | 38 | 0.200±0.320 | 0.150±0.200 | 26.6±1.4w | 18 months | moring | Not reported | Partial | ELISA |
|  |  |  |  |  | Saliva | 58 | 38 | 0.230±0.450 | 0.150±0.200 | 30.6±0.9w | 18 months | moring | Not reported | Partial |  |
| K.L. Watterberg.et al | 2019.11 | USA | 3 | 8 | Saliva | 219 | 40 | 0.210±0.134 | 0.270±0.134 | 26.40±1.03w | 6.9±0.43yers old | moring | Partial | Not reported | ELISA |
|  |  |  |  |  | Saliva | 219 | 40 | 0.260±0.067 | 0.290±0.067 | 26.40±1.03w | 6.9±0.43yers old | moring | Partial | Not reported |  |
|  |  |  |  |  | Saliva | 219 | 40 | 0.020±0.045 | 0.040±0.045 | 26.40±1.03w | 6.9±0.43yers old | evening | Partial | Not reported |  |
|  |  |  |  |  | Saliva | 219 | 40 | 0.120±0.022 | 0.130±0.022 | 26.40±1.03w | 6.9±0.43yers old | pre-trial | Partial | Not reported |  |
| Marie W.et al | 2014.11 | Sweden | 3 | 8 | Saliva | 42 | 46 | 0.212±0.101 | 0.320±0.133 | 25-34w | 7 years old | moring | Not reported | Not reported | ELISA |
|  |  |  |  |  | Saliva | 42 | 46 | 0.097±0.053 | 0.115±0.048 | 25-34w | 7 years old | noon | Not reported | Not reported |  |
|  |  |  |  |  | Saliva | 42 | 46 | 0.018±0.011 | 0.022±0.013 | 25-34w | 7 years old | evening | Not reported | Not reported |  |
|  |  |  |  |  | Saliva | 47 | 43 | 0.212±0.141 | 0.256±0.101 | 25-34w | 9 years old | moring | Not reported | Not reported |  |
|  |  |  |  |  | Saliva | 47 | 43 | 0.097±0.040 | 0.097±0.056 | 25-34w | 9 years old | noon | Not reported | Not reported |  |
|  |  |  |  |  | Saliva | 47 | 43 | 0.036±0.019 | 0.061±0.040 | 25-34w | 9 years old | evening | Not reported | Not reported |  |
| Susanne B.et al | 2015.1 | Canada | 3 | 8 | Saliva | 20 | 33 | 0.257±0.143 | 0.266±0.167 | 31.3±1.2w | 7 years old | moring | Not reported | No | ELISA |
|  |  |  |  |  | Saliva | 20 | 33 | 0.099±0.031 | 0.101±0.080 | 31.3±1.2w | 7 years old | noon | Not reported | No |  |
|  |  |  |  |  | Saliva | 20 | 33 | 0.074±0.358 | 0.063±0.109 | 31.3±1.2w | 7 years old | afternoon | Not reported | No |  |
|  |  |  |  |  | Saliva | 20 | 33 | 0.021±0.040 | 0.015±0.086 | 31.3±1.2w | 7 years old | evening | Not reported | No |  |
|  |  |  |  |  | Saliva | 47 | 33 | 0.270±1.851 | 0.266±0.167 | 31.3±1.2w | 7 years old | moring | Not reported | No |  |
|  |  |  |  |  | Saliva | 47 | 33 | 0.094±0.137 | 0.101±0.080 | 31.3±1.2w | 7 years old | noon | Not reported | No |  |
|  |  |  |  |  | Saliva | 47 | 33 | 0.072±0.069 | 0.063±0.109 | 31.3±1.2w | 7 years old | afternoon | Not reported | No |  |
|  |  |  |  |  | Saliva | 47 | 33 | 0.024±0.041 | 0.015±0.086 | 31.3±1.2w | 7 years old | evening | Not reported | No |  |
| A.A.Quesada.et al | 2014.11 | Germany | 3 | 7 | Saliva | 30 | 31 | 0.478±0.024 | 0.374±0.022 | 32.23±3.03w | 8 years old | moring | Partial | Not reported | ELISA |
|  |  |  |  |  | Saliva | 30 | 31 | 0.716±0.056 | 0.738±0.052 | 32.23±3.03w | 8 years old | moring | Partial | Not reported |  |
|  |  |  |  |  | Saliva | 30 | 31 | 0.110±0.009 | 0.128±0.011 | 32.23±3.03w | 8 years old | afternoon | Partial | Not reported |  |
|  |  |  |  |  | Saliva | 30 | 31 | 0.048±0.011 | 0.039±0.013 | 32.23±3.03w | 8 years old | evening | Partial | Not reported |  |
|  |  |  |  |  | Saliva | 30 | 31 | 0.135±0.016 | 0.115±0.018 | 32.23±3.03w | 8 years old | pre-trial | Partial | Not reported |  |
| Natalie M.et al | 2016.1 | Switzerland | 3 | 8 | Saliva | 85 | 91 | 0.252±0.017 | 0.323±0.014 | 29.7±2w | 9.5±1.4 years old | awake | Partial | Partial | FIA |
|  |  |  |  |  | Saliva | 85 | 91 | 0.325±0.019 | 0.395±0.018 | 29.7±2w | 9.5±1.4 years old | 10min after awake | Partial | Partial |  |
|  |  |  |  |  | Saliva | 85 | 91 | 0.400±0.018 | 0.497±0.022 | 29.7±2w | 9.5±1.4 years old | 20min after awake | Partial | Partial |  |
|  |  |  |  |  | Saliva | 85 | 91 | 0.397±0.023 | 0.487±0.022 | 29.7±2w | 9.5±1.4 years old | 30min after awake | Partial | Partial |  |
| Auriana U.et al | 2021.12 | Switzerland | 3 | 8 | Saliva | 33 | 11 | 0.784±0.450 | 0.229±0.450 | 30.11±1.84w | 9 years old | moring | Not reported | Not reported | ELISA |
|  |  |  |  |  | Saliva | 33 | 11 | 0.235±0.151 | 0.074±0.151 | 30.11±1.84w | 9 years old | noon | Not reported | Not reported |  |
|  |  |  |  |  | Saliva | 33 | 11 | 0.280±0.178 | 0.061±0.178 | 30.11±1.84w | 9 years old | afternoon | Not reported | Not reported |  |
|  |  |  |  |  | Saliva | 33 | 11 | 0.057±0.020 | 0.082±0.020 | 30.11±1.84w | 9 years old | evening | Not reported | Not reported |  |
| Gonzalo D.et al | 2023.10 | Canada | 3 | 8 | Saliva | 18 | 40 | 0.158±0.102 | 0.223±0.150 | 24-27w | 5.1~8.5 years old | moring | Not reported | Not reported | LC-MS/MS |
|  |  |  |  |  | Saliva | 43 | 40 | 0.151±0.140 | 0.223±0.150 | 28-32w | 5.0~7.9 years old | moring | Not reported | Not reported |  |
|  |  |  |  |  | Saliva | 18 | 40 | 0.040±0.001 | 0.054±0.051 | 24-27w | 5.1~8.5 years old | noon | Not reported | Not reported |  |
|  |  |  |  |  | Saliva | 43 | 40 | 0.065±0.037 | 0.054±0.051 | 28-32w | 5.0~7.9 years old | noon | Not reported | Not reported |  |
|  |  |  |  |  | Saliva | 18 | 40 | 0.023±0.025 | 0.036±0.027 | 24-27w | 5.1~8.5 years old | afternoon | Not reported | Not reported |  |
|  |  |  |  |  | Saliva | 43 | 40 | 0.029±0.010 | 0.036±0.027 | 28-32w | 5.0~7.9 years old | afternoon | Not reported | Not reported |  |
|  |  |  |  |  | Saliva | 18 | 40 | 0.022±0.060 | 0.022±0.013 | 24-27w | 5.1~8.5 years old | evening | Not reported | Not reported |  |
|  |  |  |  |  | Saliva | 43 | 40 | 0.022±0.007 | 0.022±0.013 | 28-32w | 5.0~7.9 years old | evening | Not reported | Not reported |  |
| ABuske-Kirschbaum.et al | 2007.11 | Germany | 3 | 8 | Saliva | 18 | 18 | 0.143±0.014 | 0.177±0.016 | 31±2.9w | 10.53±1.29 years old | pre-trial | No | Not reported | FIA |
|  |  |  |  |  | Saliva | 18 | 18 | 0.591±0.053 | 0.437±0.030 | 31±2.9w | 10.53±1.29 years old | awake | No | Not reported |  |
|  |  |  |  |  | Saliva | 18 | 18 | 0.666±0.050 | 0.631±0.043 | 31±2.9w | 10.53±1.29 years old | 30min after awake | No | Not reported |  |
|  |  |  |  |  | Saliva | 18 | 18 | 0.511±0.010 | 0.542±0.015 | 31±2.9w | 10.53±1.29 years old | 45min after awake | No | Not reported |  |
|  |  |  |  |  | Saliva | 18 | 18 | 0.474±0.040 | 0.446±0.040 | 31±2.9w | 10.53±1.29 years old | 60min after awake | No | Not reported |  |
| J.B. Pitcher.et al | 2012.12 | UK | 4 | 7 | Saliva | 9 | 5 | 0.117±0.040 | 0.169±0.047 | 29.9±2w | 13.8 ± 0.5 years old | afternoon | Not reported | Not reported | ELISA |
|  |  |  |  |  | Saliva | 9 | 5 | 0.148±0.068 | 0.169±0.047 | 29.9±2w | 13.8 ± 0.5 years old | afternoon | Not reported | Not reported |  |
| Callie L.et al | 2020.5 | USA | 3 | 8 | Saliva | 171 | 50 | 0.050±0.059 | 0.060±0.044 | 27.9±2.7w | 14.5±0.3 years old | pre-trial | Partial | Partial |  |
| Emma S.et al | 2015.11 | Sweden | 4 | 7 | Saliva | 49 | 43 | 0.158±0.105 | 0.135±0.134 | - | 12~17 years old | pre-trial | Not reported | Not reported | ELISA |
| Nina K.et al | 2016.11 | Finland | 3 | 8 | Saliva | 49 | 36 | 0.284±0.079 | 0.281±0.061 | 29.3±2.4w | 23.3±2.1years old | awake | Not reported | Partial | FIA |
|  |  |  |  |  | Saliva | 49 | 36 | 0.551±0.061 | 0.569±0.050 | 29.3±2.4w | 23.3±2.1years old | 30min after awake | Not reported | Partial |  |
|  |  |  |  |  | Saliva | 49 | 36 | 0.054±0.144 | 0.036±0.079 | 29.3±2.4w | 23.3±2.1years old | evening | Not reported | Partial |  |
| Ruth E.et al | 2004.7 | Canada | 3 | 7 | Saliva | 19 | 22 | 0.326±0.049 | 0.190±0.019 | 26.6±1.8w | 8 months | pre-trial | Not reported | Not reported | ELISA |
|  |  |  |  |  | Saliva | 34 | 22 | 0.177±0.013 | 0.190±0.019 | 30.9±1.4w | 8 months | pre-trial | Not reported | Not reported |  |

**Supplementary Table5:** Characteristics of the individual studies included in the meta-analysis.Evidence level (EL) of each study was based on Oxford Centre for Evidence-Based Medicine 2011. Quality (Q) of each study was based on the Newcastle-Ottawa Quality. GA：Gestational Age. PB: peripheral blood. UCB: umbilical cord blo. RIA:including radioimmunoassay. ELISA: enzyme-Linked Immunosorbent assay. FIA: fluorescence immunoassay. LC-MS/MS: liquid chromatography-tandem mass spectrometry.

| **Author[Ref.]** | **Publish  Year** | **Couontry** | **Specimen** | **NOS quality accessment** | | | **Total** |
| --- | --- | --- | --- | --- | --- | --- | --- |
|  |  |  |  | **Seclection** | **Comparability** | **Outcomes** |  |
| Murphy B.et al | 1974.8 | Canada | UCB | 3 | 1 | 3 | 7 |
| Sybulski S.et al | 1976.5 | Canada | UCB | 3 | 1 | 3 | 7 |
| Blumenthal N.et al | 1983.4 | South African | UCB | 3 | 1 | 3 | 7 |
| Shen X.et al | 1988.7 | China | UCB | 3 | 1 | 3 | 7 |
| Simon T.et al | 2018.3 | France | PB/UCB | 3 | 1 | 3 | 7 |
| Kurki T.et al | 1991.7 | Finland | UCB | 3 | 2 | 3 | 8 |
| Srivastava T.et al | 1994.8 | India | UCB | 3 | 2 | 3 | 8 |
| Li Z.et al | 1994.9 | China | UCB | 3 | 2 | 3 | 8 |
| Elizabeth T.et al | 2004.8 | USA | UCB | 3 | 2 | 3 | 8 |
| Masahiro M.et al | 2005.11 | Japan | UCB | 3 | 2 | 3 | 8 |
| Ding Y.et al | 2007.4 | China | UCB | 2 | 2 | 3 | 7 |
| Thi T.et al | 2009 | Australia | UCB | 3 | 2 | 3 | 8 |
| Afzal A.et al | 2016.5 | India | UCB | 3 | 2 | 3 | 8 |
| Afzal A.et al | 2016.11 | India | UCB | 3 | 1 | 3 | 7 |
| Braun T.et al | 2018.11 | Germany | UCB | 3 | 2 | 3 | 8 |
| Rabiepoor R.et al | 2019.12 | Iran | UCB | 3 | 1 | 3 | 7 |
| Ravivarma R.et al | 2022.12 | India | UCB | 3 | 1 | 3 | 7 |
| Huang M.et al | 1998.4 | China | PB | 2 | 0 | 3 | 5 |
| Luo X.et al | 1988.4 | China | PB | 2 | 1 | 2 | 5 |
| Doerr H.et al | 1988.5 | Germany | PB/UCB | 3 | 1 | 2 | 6 |
| Zhao M.et al | 1989.8 | China | PB | 3 | 0 | 3 | 6 |
| Lee M.et al | 1989.12 | USA | PB | 3 | 0 | 3 | 6 |
| Zhao M.et al | 1990.8 | China | PB | 2 | 0 | 3 | 5 |
| W Rokicki.et al | 1990 | Poland | PB | 3 | 0 | 3 | 6 |
| Zhu X. et al | 1992.6 | China | PB | 3 | 0 | 3 | 6 |
| Economou G. et al | 1993.5 | Greece | PB | 3 | 2 | 3 | 8 |
| Metzger D.et al | 1993.8 | USA | PB | 3 | 2 | 3 | 8 |
| Zhu X. et al | 1993.10 | China | PB | 3 | 0 | 3 | 6 |
| Ng P.et al | 2000.11 | China | PB | 3 | 2 | 3 | 8 |
| Jefferies C.et al | 2004.3 | New Zealand | PB | 2 | 1 | 3 | 6 |
| Sun Z.et al | 2004.6 | China | PB | 2 | 1 | 3 | 6 |
| Cai L.et al | 2005.5 | China | PB | 3 | 2 | 3 | 8 |
| He X.et al | 2007.2 | China | PB | 3 | 2 | 3 | 8 |
| M.G.Ballerini.et al | 2010.1 | Argentina | PB | 2 | 0 | 3 | 5 |
| Chen C.et al | 2012.3 | China | PB | 3 | 2 | 3 | 8 |
| Ge Y.et al | 2012.11 | China | PB | 3 | 1 | 3 | 7 |
| Zhu W.et al | 2013.6 | China | PB | 3 | 1 | 3 | 7 |
| Nina K.et al | 2014.1 | Finland | Saliva/PB | 3 | 1 | 3 | 7 |
| Mei G.et al | 2014.6 | China | PB | 3 | 1 | 3 | 7 |
| A Kistner.et al | 2014.11 | Sweden | PB | 3 | 1 | 3 | 7 |
| Cha P.et al | 2015.9 | China | PB | 3 | 2 | 3 | 8 |
| Yang R.et al | 2018.8 | China | PB | 3 | 1 | 3 | 7 |
| Miranda D.et al | 2020.3 | Netherlands | PB | 3 | 2 | 2 | 7 |
| Arsinoi K.et al | 2021.12 | Greece | PB | 3 | 2 | 2 | 7 |
| Marta O.et al | 2023.5 | Poland | Saliva | 3 | 2 | 3 | 8 |
| Katrin I.et al | 2012.3 | Sweden | Saliva | 3 | 1 | 3 | 7 |
| David W.et al | 2006.1 | Canada | Saliva | 3 | 1 | 3 | 7 |
| Ruth E.et al | 2007.2 | Canada | Saliva | 3 | 2 | 3 | 8 |
| David W.et al | 2008.11 | UK | Saliva | 3 | 1 | 3 | 7 |
| Katrin I.et al | 2015.3 | Germany | Saliva | 3 | 1 | 2 | 7 |
| Livio P.et al | 2016.10 | Italy | Saliva | 3 | 1 | 3 | 7 |
| Bettendorf M.et al | 1998.4 | Germany | Saliva | 3 | 2 | 3 | 8 |
| David Q.et al | 2022.11 | UK | Saliva | 3 | 2 | 3 | 8 |
| Ruth E.et al | 2010.1 | Canada | Saliva | 3 | 2 | 3 | 8 |
| Sarah J.et al | 2013.6 | USA | Saliva | 3 | 2 | 3 | 8 |
| Stephanie H.et al | 2014.12 | Switzerland | Saliva | 3 | 2 | 3 | 8 |
| Mai T.et al | 2007.3 | Canada | Saliva | 3 | 2 | 3 | 8 |
| Marta O.et al | 2023 | Poland | Saliva | 3 | 2 | 3 | 8 |
| Lorna G.et al | 2022.7 | UK | Saliva | 3 | 2 | 3 | 8 |
| Susanne B.et al | 2011.3 | Canada | Saliva | 3 | 2 | 3 | 8 |
| M.A. McLean.et al | 2023.5 | Canada | Saliva | 3 | 2 | 2 | 7 |
| K.L. Watterberg.et al | 2019.11 | USA | Saliva | 3 | 2 | 3 | 8 |
| Marie W.et al | 2014.11 | Sweden | Saliva | 3 | 2 | 3 | 8 |
| Susanne B.et al | 2015.1 | Canada | Saliva | 3 | 2 | 3 | 8 |
| A.A.Quesada.et al | 2014.11 | Germany | Saliva | 2 | 2 | 3 | 7 |
| Natalie M.et al | 2016.1 | Switzerland | Saliva | 3 | 2 | 3 | 8 |
| Auriana U.et al | 2021.12 | Switzerland | Saliva | 3 | 2 | 3 | 8 |
| Gonzalo D.et al | 2023.10 | Canada | Saliva | 3 | 2 | 3 | 8 |
| ABuske-Kirschbaum.et al | 2007.11 | Germany | Saliva | 3 | 2 | 3 | 8 |
| J.B. Pitcher.et al | 2012.12 | UK | Saliva | 3 | 1 | 3 | 7 |
| Callie L.et al | 2020.5 | USA | Saliva | 3 | 2 | 3 | 8 |
| Emma S.et al | 2015.11 | Sweden | Saliva | 3 | 1 | 3 | 7 |
| Nina K.et al | 2016.11 | Finland | Saliva | 3 | 2 | 2 | 8 |
| Ruth E.et al | 2004.7 | Canada | Saliva | 3 | 1 | 3 | 7 |

**Supplementary Table 6**.Quality assessment of studies using Newcastle Ottawa Scale.

| **Moderator** | **Estimate** | **SE** | **Z value** | **p value** | **95% CI** | **QM (df)** | **QM p** | **QE (df)** | **QE p** | **I² (%)** | **R²(%)** | **τ²** |
| --- | --- | --- | --- | --- | --- | --- | --- | --- | --- | --- | --- | --- |
| **specimen type** (Ref: PB) |  |  |  |  |  | 10.1(2) | **0.0064** | 530.91(74) | <0.0001 | 86.06 | 7.6 | 0.3067 (SE=0.0670) |
| Salivary | -0.1855 | 0.1606 | -1.1544 | 0.2483 | -0.5003 to 0.1294 | - | - | - | - | - | - | - |
| UCB | -0.5948 | 0.1881 | -3.1619 | 0.0016 | -0.9635 to -0.2261 | - | - | - | - | - | - | - |
| **Measurement Method**  (Ref: ELISA) |  |  |  |  |  | 4.5252(3) | 0.2101 | 578.01(73) | <0.0001 | 87.37 | 0 | 0.3449 (SE=0.0750) |
| FIA | 0.3437 | 0.2049 | 1.6778 | 0.0934 | -0.0578 to 0.7452 | - | - | - | - | - | - | - |
| LC-MS/MS | -0.2135 | 0.2734 | -0.7809 | 0.4348 | -0.7494 to 0.3224 | - | - | - | - | - | - | - |
| RIA | 0.0701 | 0.1767 | 0.3967 | 0.6916 | -0.2763 to 0.4165 | - | - | - | - | - | - | - |
| **Prenatal Corticosteroid Use** (Ref: No) |  |  |  |  |  | 8.5168（3） | **0.0365** | 541.22（73) | <0.0001 | 86.51 | 3.75 | 0.3194 (SE=0.0702) |
| Not Reported | 0.3096 | 0.1629 | 1.9009 | 0.0573 | -0.0096 to 0.6288 | - | - | - | - | - | - | - |
| Partial | 0.1643 | 0.215 | 0.7644 | 0.4446 | -0.2570 to 0.5857 | - | - | - | - | - | - | - |
| Yes | -0.6133 | 0.3707 | -1.6543 | 0.0981 | -1.3399 to 0.1133 | - | - | - | - | - | - | - |
| **Postnatal Corticosteroid Use** (Ref: No) |  |  |  |  |  | 3.8424（2） | 0.1464 | 587.62（74） | <0.0001 | 87.41 | 0 | 0.3451 (SE=0.0747) |
| Not Reported | -0.0443 | 0.174 | -0.2547 | 0.799 | -0.3853 to 0.2967 | - | - | - | - | - | - | - |
| Partial | 0.3763 | 0.2421 | 1.5546 | 0.12 | -0.0981 to 0.8508 | - | - | - | - | - | - | - |
| **Age**  **(Ref: ≤1 year)** | 0.2854 | 0.1697 | 1.682 | 0.0926 | -0.0472 to 0.6180 | 2.829（1） | **0.0926** | 587.59（75） | <0.0001 | 87.24 | 0 | 0.3370 (SE=0.0727) |

**Supplementary table 7.** Results of univariate meta-regression analyses exploring potential moderators of heterogeneity. QM, test statistic for moderators; QE, test statistic for residual heterogeneity; SE, standard error; CI, confidence interval; I², percentage of variation attributable to heterogeneity; R², proportion of heterogeneity explained; τ², amount of residual heterogeneity

| **Moderator** | **Estimate** | **SE** | **Z value** | **p value** | **95% CI** | **QM (df)** | **QM p** | **QE (df)** | **QE p** | **I² (%)** | **R²(%)** | **τ²** |
| --- | --- | --- | --- | --- | --- | --- | --- | --- | --- | --- | --- | --- |
| **specimen type (Ref: PB)** |  |  |  |  |  | 14.87(6) | 0.0213 | 507.86(70) | <0.001 | 86.22 | 4.45 | 0.3171 (SE=0.0710) |
| Salivary | -0.2737 | 0.1827 | -1.4982 | 0.1341 | -0.6318 to 0.0844 | - | - | - | - | - | - | - |
| UCB | -0.4469 | 0.2078 | -2.1505 | 0.0315* | -0.8542 to -0.0396 | - | - | - | - | - | - | - |
| **Prenatal Corticosteroid Use (Ref: No)** |  |  |  |  |  | - | - | - | - | - | - | - |
| Not Reported | 0.2138 | 0.1738 | 1.2307 | 0.2184 | -0.1267 to 0.5544 | - | - | - | - | - | - | - |
| Partial | 0.071 | 0.2441 | 0.2908 | 0.7712 | -0.4075 to 0.5495 | - | - | - | - | - | - | - |
| Yes | -0.3791 | 0.3905 | -0.9708 | 0.3316 | -1.1446 to 0.3863 | - | - | - | - | - | - | - |
| **Age**  **(Ref: ≤1 year)** | 0.2089 | 0.1958 | 1.0671 | 0.2859 | -0.1748 to 0.5926 | - | - | - | - | - | - | - |

**Supplementary table 8.** Results of multivariate meta-regression analyses exploring potential moderators of heterogeneity. QM, test statistic for moderators; QE, test statistic for residual heterogeneity; SE, standard error; CI, confidence interval; I², percentage of variation attributable to heterogeneity; R², proportion of heterogeneity explained; τ², amount of residual heterogeneity

| **Exposure** | **Outcome** | **MR** | | | | |
| --- | --- | --- | --- | --- | --- | --- |
|  |  | **Method** | **nSNP** | **β** | **SE** | **p** |
| preterm delivery | cortisone levels | IVW | 52 | -0.010829892 | 0.005154107 | 0.035622021 |
|  |  | MR Egger | 52 | -0.031833856 | 0.021552536 | 0.145937473 |
|  |  | WM | 52 | -0.01171767 | 0.006731564 | 0.081735233 |
|  |  | SM | 52 | -0.011539217 | 0.014676634 | 0.435370495 |
|  |  | Weighted mode | 52 | -0.012852254 | 0.012818615 | 0.320775723 |

# Supplementary Table 9: MR estimates of the effect of mediators on outcomes.

| **Section and Topic** | **Item** | **Checklist item** | **Location where item is reported** |
| --- | --- | --- | --- |
| **TITLE** | | |  |
| Title | 1 | Identify the report as a systematic review. | P1 |
| **ABSTRACT** | | |  |
| Abstract | 2 | See the PRISMA 2020 for Abstracts checklist. | P1 |
| **INTRODUCTION** | | |  |
| Rationale | 3 | Describe the rationale for the review in the context of existing knowledge. | P2 |
| Objectives | 4 | Provide an explicit statement of the objective(s) or question(s) the review addresses. | P2 |
| **METHODS** | | |  |
| Eligibility criteria | 5 | Specify the inclusion and exclusion criteria for the review and how studies were grouped for the syntheses. | P3-4 |
| Information sources | 6 | Specify all databases, registers, websites, organisations, reference lists and other sources searched or consulted to identify studies. Specify the date when each source was last searched or consulted. | P3 |
| Search strategy | 7 | Present the full search strategies for all databases, registers and websites, including any filters and limits used. | P3,and Supplementary Table 1 |
| Selection process | 8 | Specify the methods used to decide whether a study met the inclusion criteria of the review, including how many reviewers screened each record and each report retrieved, whether they worked independently, and if applicable, details of automation tools used in the process. | P4 |
| Data collection process | 9 | Specify the methods used to collect data from reports, including how many reviewers collected data from each report, whether they worked independently, any processes for obtaining or confirming data from study investigators, and if applicable, details of automation tools used in the process. | P3-4 |
| Data items | 10a | List and define all outcomes for which data were sought. Specify whether all results that were compatible with each outcome domain in each study were sought (e.g. for all measures, time points, analyses), and if not, the methods used to decide which results to collect. | P4 |
|  | 10b | List and define all other variables for which data were sought (e.g. participant and intervention characteristics, funding sources). Describe any assumptions made about any missing or unclear information. | P4 |
| Study risk of bias assessment | 11 | Specify the methods used to assess risk of bias in the included studies, including details of the tool(s) used, how many reviewers assessed each study and whether they worked independently, and if applicable, details of automation tools used in the process. | P4-5 |
| Effect measures | 12 | Specify for each outcome the effect measure(s) (e.g. risk ratio, mean difference) used in the synthesis or presentation of results. | P5 |
| Synthesis methods | 13a | Describe the processes used to decide which studies were eligible for each synthesis (e.g. tabulating the study intervention characteristics and comparing against the planned groups for each synthesis (item #5)). | P5-6 |
|  | 13b | Describe any methods required to prepare the data for presentation or synthesis, such as handling of missing summary statistics, or data conversions. | P5 |
|  | 13c | Describe any methods used to tabulate or visually display results of individual studies and syntheses. | P5 |
|  | 13d | Describe any methods used to synthesize results and provide a rationale for the choice(s). If meta-analysis was performed, describe the model(s), method(s) to identify the presence and extent of statistical heterogeneity, and software package(s) used. | P5 |
|  | 13e | Describe any methods used to explore possible causes of heterogeneity among study results (e.g. subgroup analysis, meta-regression). | P5 |
|  | 13f | Describe any sensitivity analyses conducted to assess robustness of the synthesized results. | P5 |
| Reporting bias assessment | 14 | Describe any methods used to assess risk of bias due to missing results in a synthesis (arising from reporting biases). | P5 |
| Certainty assessment | 15 | Describe any methods used to assess certainty (or confidence) in the body of evidence for an outcome. | P5 |
| **RESULTS** | | |  |
| Study selection | 16a | Describe the results of the search and selection process, from the number of records identified in the search to the number of studies included in the review, ideally using a flow diagram. | P5 and figure1 |
|  | 16b | Cite studies that might appear to meet the inclusion criteria, but which were excluded, and explain why they were excluded. | P5 and figure1 |
| Study characteristics | 17 | Cite each included study and present its characteristics. | P5-6, Supplementary Table 5 |
| Risk of bias in studies | 18 | Present assessments of risk of bias for each included study. | P6,Supplementary Table 6 |
| Results of individual studies | 19 | For all outcomes, present, for each study: (a) summary statistics for each group (where appropriate) and (b) an effect estimate and its precision (e.g. confidence/credible interval), ideally using structured tables or plots. | P6-8 |
| Results of syntheses | 20a | For each synthesis, briefly summarise the characteristics and risk of bias among contributing studies. | P6-8 |
|  | 20b | Present results of all statistical syntheses conducted. If meta-analysis was done, present for each the summary estimate and its precision (e.g. confidence/credible interval) and measures of statistical heterogeneity. If comparing groups, describe the direction of the effect. | P6-8 |
|  | 20c | Present results of all investigations of possible causes of heterogeneity among study results. | P6-8, subgroup analysis,meta-regressio |
|  | 20d | Present results of all sensitivity analyses conducted to assess the robustness of the synthesized results. | P8,Supplementary Figure 26-29 |
| Reporting biases | 21 | Present assessments of risk of bias due to missing results (arising from reporting biases) for each synthesis assessed. | P8 |
| Certainty of evidence | 22 | Present assessments of certainty (or confidence) in the body of evidence for each outcome assessed. | P6,Supplementary Table 5 |
| **DISCUSSION** | | |  |
| Discussion | 23a | Provide a general interpretation of the results in the context of other evidence. | P8-11 |
|  | 23b | Discuss any limitations of the evidence included in the review. | P10-11 |
|  | 23c | Discuss any limitations of the review processes used. | P10-11 |
|  | 23d | Discuss implications of the results for practice, policy, and future research. | P11 |
| **OTHER INFORMATION** | | |  |
| Registration and protocol | 24a | Provide registration information for the review, including register name and registration number, or state that the review was not registered. | P4,  PROSPERO:CRD42024606328 |
|  | 24b | Indicate where the review protocol can be accessed, or state that a protocol was not prepared. | PROSPERO:CRD42024606328 |
|  | 24c | Describe and explain any amendments to information provided at registration or in the protocol. | There were no amendments |
| Support | 25 | Describe sources of financial or non-financial support for the review, and the role of the funders or sponsors in the review. | P12 |
| Competing interests | 26 | Declare any competing interests of review authors. | P11 |
| Availability of data, code and other materials | 27 | Report which of the following are publicly available and where they can be found: template data collection forms; data extracted from included studies; data used for all analyses; analytic code; any other materials used in the review. |  |

# Supplementary Table 10: PRISMA 2020 checklist
